# Supplementary material for: A computational account of multiple motives guiding context-dependent prosocial behavior
Source: PLoS Comput Biol. 2025 Apr 21;21(4):e1013032. doi: 10.1371/journal.pcbi.1013032 (PMC12112419; doi:10.1371/journal.pcbi.1013032)
Supplement: S1 Text — (DOCX) [file pcbi.1013032.s001.docx]

**S1 Text. Supplementary Information**

A Computational Account of the Multiple Motives Guiding Context-dependent Prosocial Behavior

Claire Lugrin^1^, Jie Hu^1,2*^, Christian C. Ruff^1,3,4*^

^1^ Zurich Center for Neuroeconomics (ZNE), Department of Economics, University of Zurich, Zurich, Switzerland

^2^ Shanghai Key Laboratory of Mental Health and Psychological Crisis Intervention, School of Psychology and Cognitive Science, East China Normal University, Shanghai, China

^3^ Faculty of Medicine, University of Zurich, Zurich, Switzerland
^4^ URPP Adaptive Brain Circuits in Development and Learning (URPP AdaBD), University of Zurich, Zurich, Switzerland

* jhu@psy.ecnu.edu.cn, [christian.ruff@uzh.ch](mailto:christian.ruff@uzh.ch)

**Supplementary methods**

**Time estimation game**

A time estimation game allowed participants to earn points before they faced social decisions. The participants had to estimate an amount of time and earned points depending on the accuracy of their estimation. On each trial, participants were shown the amount of time they had to estimate, ranging from 1 to 5 seconds, with a one-decimal precision. A black fixation cross in the center of the screen turned red 1.5 to 2.5 seconds after the instructions appeared, indicating that people should start estimating time (starting cue). Participants then had to press the spacebar on their keyboard after they considered the right time had elapsed since the red cross’ appearance.

On each trial, 2 to 10 points were available for the participants to collect. They earned a percentage of these points depending on the accuracy of their estimation. Subjects were informed of this rule but were not told the number of points available for each trial and were not given feedback on their time estimation accuracy. They were informed about the number of points they collected, immediately after each trial. This way, we ensured that participants could not know if they received points because they performed well or because many points were available. This is important to prevent people from deciding based on merit during the action task. The points earned during a trial t (Earnings(t)) could take the values 2, 4, 6, 8, and 10 and were computed using the following formula:

$$Earnings(t)=[MaxDiff-abs(TimeToEstimate(t)-TimeEstimated(t))]\times[MaxOutcome(t)-2]+2$$

Where $\mathrm{TimeToEstimate}$ is the time participants were asked to estimate on a given trial and $\mathrm{TimeEstimated}$ the estimation they provided. MaxOutcome is the number of points available on trial t, ranging from 2 to 12. We used the time estimation data from Experiment 1 to predetermine the frequency of each maximum outcome during the experiment (S1b Fig represents this distribution). The maximum number of points of 12 was included to ensure that some participants would get 10 points on some trials, even if they performed poorly. MaxDiff represents the time estimation error tolerance (the maximum difference between the time to estimate and the time estimated that would allow people to get more than the minimum number of points). This tolerance was set to 1s at the beginning of the experiment and was then adjusted for each participant every 25 trials. For this adjustment, we compared the distribution of points collected by the participant with 100 randomly generated sets of points drawn from a uniform distribution (each random set having the same number of samples as the participant’s distribution). If the difference in frequency between the most and the least frequent number of points collected by the participant was higher than this difference for any randomly generated distribution, MaxDiff was modified for the next trials. It was lowered to 0.8s for participants that had collected more often 8 and 10 points than 2 and 4 points and increased to 1.2s in the opposite case.

The earnings were rounded to the nearest point (2, 4, 6, 8, or 10). Each trial yielded a minimum of 2 points (and a maximum of 10). We excluded outcomes of 0 points as these had a different effect on participant behavior compared to non-zero outcomes in a previous pilot study.

The manipulations described above were used to ensure that all participants faced a roughly equal number of trials for each of the 5 possible earnings while avoiding deception. This way, the more accurate the participants were, the more points they collected, but they still faced enough trials of each type (See S1 Fig).

The time to estimate was drawn from the same distribution for all participants (Players A and Players B) ensuring that all participants faced trials of equal difficulty.

**Two versions of the Action task**

Two versions of the action task differed in the way the trials were arranged and presented to Players A, and in details of the instructions. In the first version, Players A had to decide whether to Get or Don’t get the bonus, whereas in version 2 the response options were Help, Don’t help, or Erase, Don’t erase depending on the context. The display of the response options was different for the two versions (see S1 Fig for details of the two versions). The points of Player B and the context were made more salient in version 2 compared to version 1. To increase the salience of the context in version 2, the trials were grouped in blocks of 10 trials of the same frame, and one background color (pink or purple) was attributed to each frame. A lottery (that could erase the points of Players B) was computing the frame every 10 trials. In version 1 of the task, the lottery was randomly computing the frame on each trial, and the background remained neutral (grey). In both versions of the task, the participants were instructed about the difference between frames and the implications of the lottery and their decisions for themselves and Players B. The Helping versus Destroying frames were emphasized in the instructions. Subjects were also extensively tested on their understanding of the consequences of their actions before starting the task.

The two versions also differed in the amount of information Player B received after the experiment. In version 1 of the task, Players B were not informed about what happened during the trials selected for their payoff. They received an email stating their payment, without any details about the trials generating this outcome. In version 2, however, Players B were mailed a letter detailing what happened during each trial that was randomly selected for their final payment. They were informed about the number of points they had collected, a number “identifying” which Player A they were paired with (Players A remained anonymous, and only referred to by their number), the bonus Player A was offered, the decision frame, and the decision of Player A. Before the experiment, Players A were shown an example of the letter sent to Player B and tested on their understanding of the information Players B would receive.

**Two versions of the Judgment task**

A first, in-person version of the judgment task matched version 1 of the action task. Subjects were shown a table representing the points earned by players A and B during the time-estimation task, the lottery outcome, and the decision of Player A. This decision was framed as “Took the bonus” and “Rejected the bonus”. Subjects had to rate the appropriateness of this action, for the 3 possible bonus values (2, 4, and 6) consecutively (S1e Fig). Subjects viewed all possible combinations of p_A_, p_B_, context, and choice of A. All these variables were randomly changed on each trial. In version 2 of this task, participants participated online and viewed the decision screen as presented to Players A (S1f Fig). The lottery was computed every 10 trials, as for version 2 of the Action task, and randomly determined the context. Subjects viewed the Actions of A framed as “Erase/Not erase” and “Help/not help”, on a red or blue background respectively (counterbalanced across participants). Subjects viewed all possible combinations of p_A_, p_B_, bonus, context, and action of A. These variables (except for the frame) were randomly changed on each trial. The choices associated with the 3 possible bonus values for each situation were therefore separated. In both versions the participants answered the questions “Please indicate how socially appropriate you believe Person A’s choice is”, by pressing the keys 1 to 6 on their keyboard, corresponding to “very socially inappropriate” (1), “socially inappropriate” (2), “somewhat socially inappropriate” (3), “somewhat socially appropriate” (4), “socially appropriate” (5), “very socially appropriate” (6).

**Design of the normative environments and feedback structure**

We chose to use the real choices of participants of Experiment 3 to provide feedback to participants of Experiment 4 in the exposure phase, rather than artificially constructed extreme behaviors. This allowed us to study the effects of realistic judgments and actions and increase the credibility of our manipulations. For each normative environment, we selected 11 participants with the most extreme behavior in the action or judgment task, resulting in feedback with different deviations from the average behavior and variability across situations (S9c-e Fig). Extreme selfish participants (A- environment: + 39% of selfish choices compared to the average of all participants of Experiment 3) picked the selfish option on almost all trials, while participants providing the most lenient judgments (J- environment + 16% in appropriateness judgments compared to the average of Experiment 3), had a more varied behavior and were influenced by the payoff structure (S9d Fig). This difference may affect the outcome of witnessing these behaviors, as confirmed by the learning performance of participants of Experiment 4 (S9e Fig): The A- pattern was easier to learn than the J- pattern (72.6% vs 47.8% correct). These effects may in part explain the high influence of frequent selfish actions on behavior compared to the minimal influence of lenient judgments. However, they represent realistic distributions of actions and judgments, and therefore are closer to behaviors participants could witness outside of the lab. Subjects of Experiment 4 observing extreme prosocial participants (A+ environment, - 26% in selfish actions compared to the average of all participants of Experiment 3) or participants providing the strictest judgments (J+ environment, - 11% in appropriateness ratings) on the other hand had a similar learning accuracy (A+: 71.4%, J+: 70.6%), despite a strong difference in the deviation of the feedback from the average behavior, showing that the strength of such deviation may not strongly impact on prosocial action rate changes.

**Experimental sessions**

***Experiment 1: Judgment task***

All in-person participants of the judgment task participated in the same session lasting 2 hours. Online participants took part in 2-hour sessions from their homes. Both the in-person and online sessions had the same structure: During the first 15 minutes, participants played 25 trials of the time-estimation task. They then performed 300 trials of the judgment task, corresponding to all possible combinations of points of A, points of B, bonus, normative context, and action of A (comply/violate). They completed the demographic questionnaire at the end of the session. All participants received a fixed payoff of 30 CHF (15 CHF per hour) plus the money they collected on 2 randomly selected time-estimation trials (0 to 10 CHF per trial) and 5 CHF for each of 3 randomly selected judgment trials if their rating corresponded to the mode ratings of other participants in the room/online session.

***Experiment 2: Action task***

**Player A**

Players A from both versions participated in 2h sessions. During the first hour, the participants played the time estimation task and collected points. During the second hour, they participated in the action task. They played on average 243 trials of the task (min 181, max 300); this number depended on how fast they completed the tasks. They then completed the demographic questionnaire. At the end of the session, participants received a fixed payoff of 30 CHF (15 CHF per hour) plus the money they collected on 3 randomly selected trials.

**Player B**

Players B from Experiment 2 participated in a 1h session (1 session per group) in which they played 250 to 300 trials of the time estimation task (depending on their speed). They received a fixed payoff of 15 CHF and were additionally mailed the money resulting from 3 randomly selected trials after Players A’s choices were implemented. Players A were informed that Players B earned the same participation fee as themselves (per hour) and would be mailed the additional money they collected right after the experiment.

***Experiment 3: Action and judgments within-subjects***

All participants of Experiment 3 were invited to two sessions. During the first session, lasting 1h, all participants played 300 trials of the time estimation task. At the end of the session, all participants were briefly informed of the action task and attributed the role of Player A or B. Players B were then uninvited from the second session.

**Player A**

Players A took part in the second session, lasting 1h20. They participated in both tasks (judgment and action), in a counterbalanced order. They played 150 trials of the action task (all possible combinations of p_A_, p_B_, and bonus; with some repeated trials depending on their time estimation performance) and 150 trials of the judgment task (all possible combinations of p_A_, p_B_, bonus, and action of A). They then completed the demographic and IRI questionnaires. At the end of the experiment, they received a fixed payoff of 35 CHF (15 CHF per hour), the money they collected on 2 randomly selected action trials, and 5 CHF for each of 2 randomly selected judgment trials if their rating corresponded to the mode ratings of other participants in the online session.

**Player B**

Players B received a fixed payoff of 15 CHF and the money remaining on 2 randomly selected trials after Players A’s choices were implemented.

***Experiment 4: Exposure to different normative environments***

Subjects of Experiment 4 were invited to two sessions. The first session was identical to Experiment 3, and randomly selected Players B were subsequently uninvited from session 2. Players B were paid in the same way as in Experiment 3.

**Player A**

Players A took part in the second session, lasting 1h40. They played 100 trials of the action task (all combinations of p_A_, p_B,_ and bonus with some repetitions depending on their time estimation performance) and 75 trials of the judgment task (all possible combinations of p_A_, p_B_, bonus). The order of the tasks was counterbalanced. After these pre-exposure measures, they played 75 trials of the exposure task. Each participant faced one type of environment only. They then played again the 100 action and 75 judgment trials and finally completed the demographic and sub-clinical trait evaluations. Subjects received a fixed payoff of 40 CHF (15 CHF per hour), the money they collected on one randomly selected action trial per phase (pre and post-exposure), 5 CHF for a randomly selected judgment trial per phase if their rating corresponded to the mode ratings of other participants in the online session, and 5 CHF for one randomly selected exposure trial, if they correctly guessed the behavior of previous participants.

***Modeling choices***

To model the choices of participants of the action task, we computed the utility difference between prosocial and selfish actions $\Delta U$ (S5 Table), and used a sigmoid (*SoftMax)* function to compute the probability that the participant chose one option over the other on trial $t$ (Here the probability of choosing the prosocial action on a given trial t $P\left( P,t \right)$).

$P\left( P,t \right) = \frac{1}{\begin{aligned} 1+e^{-\rho\Delta U\left( t \right)} \\ \end{aligned}}$

Where $\rho$ is the inverse temperature, representing the steepness of the sigmoid slope
 ($\rho= 0$) indicates that participants chose at random, increasing values of $\rho$ reflect increasingly model-consistent choices).

***Modeling judgments***

To evaluate whether the same decision processes were employed in deciding what constitutes an appropriate behavior versus acting upon it, we used the same utility models to analyze the appropriateness judgments. The difference in utility between prosocial and selfish actions $\Delta U$ was computed as described in the previous section and used to predict the ratings of participants of the judgment task. Instead of computing the probability of a binary choice (as in the action model), we computed the probability of choosing each of the 6 ratings of our Likert scale using an ordered-probit model [1]. The probability of choosing rating $k$on trial $t$ for participant $s$ was computed as follows:

$P\left( k,t,s \right) = \Phi\left( \frac{T_{s}\left[ k \right]-\Delta U\left( t \right)}{\sigma_{s}} \right)- \Phi\left( \frac{T_{s}\left[ k-1 \right]-\Delta U\left( t \right)}{\sigma_{s}} \right)$

Where $\Phi()$ is the cumulative normal function, $T_{s}$ the subject-specific thresholds between the 6 possible ratings, and $\sigma$ the subject-specific standard deviation. We fixed $T_{s}\left( 1 \right)$ ­– which separates ratings 1 (*very socially inappropriate*) and 2 (*socially inappropriate) –* to 1.5 and $T_{s}\left( 5 \right)$ – which separates rating 5 (*socially appropriate*) and 6 (*very socially inappropriate*) – to 5.5. We allowed the remaining thresholds to vary for each participant and estimated these thresholds during our model fitting procedure, to consider possible differences in ordinal scale representations by different participants.

***Hierarchical model fitting***

We fitted all models using a hierarchical structure consisting of two levels: a group-level parameter, and individual-level parameters (for each participant). We used uniform priors for the group-level parameters and normal distributions (with parameters group-level mean and standard deviation) for individual-level priors. The parameters of all participants of one experiment were fitted together using this hierarchical structure. Models were fitted separately for each normative environment of Experiment 4, as exposure to the different environments has different effects on the parameter changes.

In the action models the trial-by-trial likelihood of the observed choice$y\left( t \right)$ given the probability of prosocial action $P\left( P,t \right)$ on trial $t$ was based on a Bernoulli process:

$y\left( t \right)\sim Bernoulli\left( P\left( P,t \right) \right)$

In the judgment models, the trial-by-trial likelihood of the observed ratings$y\left( t \right)$ on trial $t$ given the probabilities $P\left( k,t \right)$ of the different ratings $k=1$ to $k=6$ was based on a categorical process:

$y\left( t \right)\sim Categorical\left( {P\left( k,t \right)}_{k = 1,2\ldots6} \right)$

We used the Gibbs sampler using the Markov Chain Monte Carlo technique provided by JAGS to estimate the posterior distribution of each parameter (at the group and individual level). We estimated 3 chains for each model and drew 5000 samples per chain after 25000 burn-in samples were drawn to ensure convergence was reached. We applied a thinning of 5 samples. We verified that all our models converged (prsf < 1.05). We concatenated the 3 chains for each parameter to constitute the posterior distribution. We used the mode of these posterior distributions as the parameter estimate for each participant.

***Model comparison***

We used a method approximating leave one out cross validation (LOO [2]) for model comparison, estimating the posterior density of unseen data (expected log pointwise predictive density), therefore taking into account both the goodness of fit of each model and the flexibility of the model (excessive number of parameters leading to overfitting and therefore a poor out-of-sample predictions).

***Clustering***

To determine the optimal number of participants types (clusters) in our data, we generated 100 sets of participants, composed of a random selection of 50% of our participants, and computed the optimal number of clusters for each of these sets using the Calinski-Harabasz [3] criterion, optimizing the between-cluster over within-cluster variance. We then chose the most frequent number of clusters across all sets, in our case, 4 clusters. We computed the coordinates of each of the 4 clusters’ centroids (in the model parameter’s space, Fig 3a, S7a & d Fig) using the data from all participants of Experiments 2 and 3 and classified all participants (from Experiments 2, 3 and 4) in the category with the closest centroid (Euclidean distance). The clusters trained on data from Experiments 2 and 3 were therefore used to classify the out-of-sample participants of Experiment 4.

To validate our approach, we repeated the cluster training procedure using data from Experiment 4 only and compared the clusters obtained when training the algorithm on Experiments 2 and 3 vs Experiment 4. The optimal number of clusters obtained using Experiment 4 was 5. Nevertheless, we found that participants got largely clustered in the same way (S14 Fig, except for participants belonging to the *Efficiency-sensitive* cluster, which were split between two separate clusters. These two new clusters varied in their baseline preference (bias values) but did not produce drastically different choices (S14c Fig, average selfish action 45 and 46%). To keep a consistent analysis across experiments, and as the differences between the two replications were small, we used the clusters trained on Experiments 2 and 3 for all analyses reported in the main text.

***Modeling changes***

To evaluate the effects of new social environments on the action and judgment models employed by our participants, we extended the models previously described to include a *change* value for each parameter. Specifically, for all parameters of the utility models, we included a *baseline* parameter value, identical for the pre- and post-exposure actions, and a *change* parameter value, weighing a dummy variable taking the value 0 for the pre-exposure trials and 1 for the post-exposure trials. These *change* parameters therefore measure the difference in the parameter value between the pre- and post-exposure phases, in other words, the effects of the environments with different social norms on the decision processes. We used the winning model identified during the previous experiments (CR bias model, excluding the bonus discount factor, as participants only faced one context (*Not Destroying*) in Experiment 4), and obtained the following models:

For trials in the pre-exposure phase:

$$\Delta U = {bias}_{baseline}+ \gamma_{baseline}\mu_{baseline}\min\left( p_{A},p_{B} \right)$$

$+\gamma_{baseline}\left( 1-\mu_{baseline} \right)\left[ p_{B}-b \right]-\left( 1-\gamma_{baseline} \right)b$

And for trials in the post-exposure phase:

$${\Delta U = bias}_{baseline}+{bias}_{change}$$

$$+ {(\gamma}_{baseline}+\gamma_{change}) (\mu_{baseline}+\mu_{change})\min\left( p_{A},p_{B} \right)$$

$+{(\gamma}_{baseline}+\gamma_{change})\left( 1-\left( \mu_{baseline}+\mu_{change} \right) \right)\left[ p_{B}-b \right]$

$-\left( 1-\left( \gamma_{baseline}+\gamma_{change} \right) \right)b$

**Supplementary results**

***Exposure to different environments changes different choice mechanisms***

We first evaluated the parameter changes across all participants, to compare the effects of the different environments on the different motives. All motives were modified following exposure (S16 Fig, S14 Table), but the four environments had different effects on each motive. First, we confirmed that exposure to the selfish versus prosocial environments had the expected effects on parameter changes. Prosocial environments (A+ and J+) increased both baseline and outcome-based preferences for prosocial actions. Selfish environments (A- and J-) increased baseline preferences for selfish actions but surprisingly had opposite effects on outcome-based preferences: Observing frequent selfish actions (A-) increased outcome-based preferences for selfish actions, whereas observing lenient judgments (J-) increased prosocial outcome-based preferences. This pattern may explain the lack of behavior change following exposure to lenient judgments: The ensuing increase in baseline preference for selfish actions but increase in outcome-based preferences for prosocial actions may counteract each other. Concerns for efficiency increased post-exposure, regardless of the environment participants faced. This was particularly pronounced for participants who had a strong concern for efficiency in the first place (S17 Fig).

Second, we compared the effects of observing descriptive (actions) versus prescriptive (judgments) behaviors, and found that the two types of information also had differential effects on motive changes. Observing actions had a stronger effect on outcome-based preferences than observing judgments (S14 Table: regression of the type of exposure on parameters changes, environment type p <0.001), especially in the negative environments (interaction environment type and direction p < 0.001). These results show that exposure to the different environments affects participants’ prosocial actions via changes in distinct decision mechanisms: Observing actual behavior affects the valuation of decision outcomes, whereas observing judgments mostly shifts participants’ bias toward one option irrespective of the action outcomes.

These general differences were accompanied by individual variations in motive changes. First, the baseline values of the different parameters were correlated with the parameter changes: Subjects with baseline parameters far away from the global mean (relying strongly on one motive) further increased their reliance on these specific motives (S14 Table, effects of the baseline parameters on parameters change p < 0.001 for all parameters, See S17 Fig for details). That is, participants who relied strongly on outcome-based preferences further increased their outcome-based preferences when facing positive norms, whereas participants relying on self-benefiting outcomes became even more selfish when observing frequent selfish actions.

**References**

1. Liddell TM, Kruschke JK. Analyzing ordinal data with metric models: What could possibly go wrong? J Exp Soc Psychol. 2018;79: 328–348. doi:10.1016/j.jesp.2018.08.009
2. Vehtari A, Gelman A, Gabry J. Practical Bayesian model evaluation using leave-one-out cross-validation and WAIC. Stat Comput. 2017;27: 1413–1432. doi:10.1007/s11222-016-9696-4
3. Caliński T, Harabasz J. A dendrite method for cluster analysis. Communications in Statistics. 1974;3: 1–27. doi:10.1080/03610927408827101
