# Supplementary figures and images for: A computational account of multiple motives guiding context-dependent prosocial behavior
Source: PLoS Comput Biol. 2025 Apr 21;21(4):e1013032. doi: 10.1371/journal.pcbi.1013032 (PMC12112419; doi:10.1371/journal.pcbi.1013032)

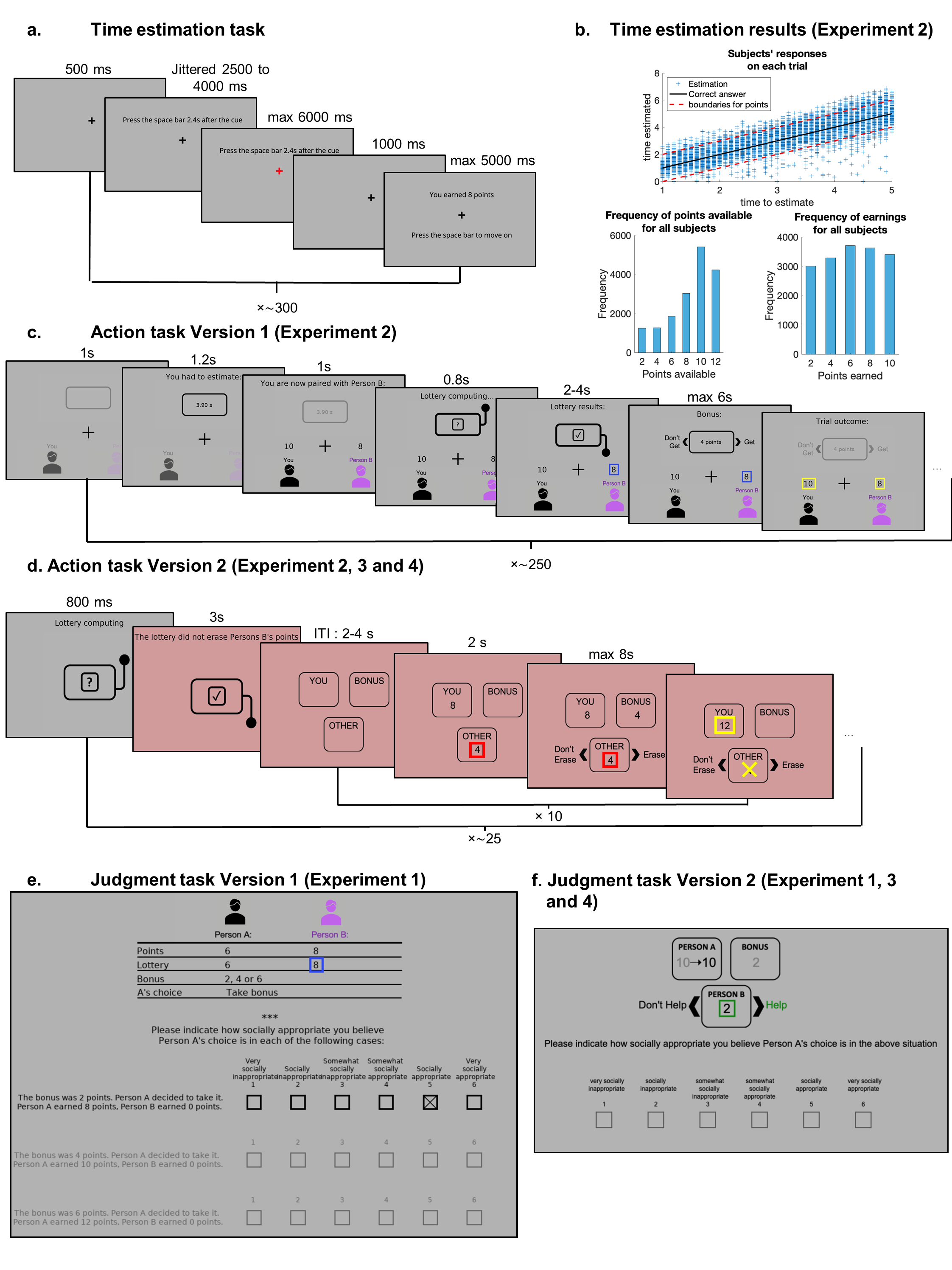

Supplement: S1 Fig — Supplement to Fig 1. (a) Time-estimation task: In the first part of Experiments 2–4, both players A and B collected points on 300 trials of this task. They were asked to estimate a specified amount of time by pressing the space bar on their keyboard at the accurate amount of time after a cue (central cross) turned red. (b) Time estimation task: Results of Experiment 2 and payoff scheme. In the top plot, each cross represents the estimation of a participant of Experiment 2, on a trial. The dashed red lines represent the boundaries outside of which the participants received the minimum number of points. The black line represents the perfect estimation. The closer the estimations were to this black line the more points participants collected. The number of points available on each trial varied across trials, the bottom left plot represents this points distribution across all trials. The participants collected a fraction of the number of points available, depending on their accuracy. The resulting distribution of points collected across participants and trials is displayed in the bottom right plot. (c) Version 1 of the action task (Experiment 2). Participants were reminded of the time estimation trial they faced and the points they collected. They were then shown the points player B collected. The lottery was computed on every trial and could erase the points of Player B. Player A was then offered some bonus points and decided whether to take or reject this bonus, with consequences for the outcome of Player B. (d) Version 2 of the action task (Experiment 2) as described in Fig 1, the trials were grouped in blocks of 10 trials between which the lottery was recomputed. Participants were not reminded of the time estimation trial that yielded their points. They decided to Erase/Not Erase (Destroying frame) or Help/Not Help (Helping frame), instead of deciding to Take/Not Take the bonus. The consequences of participants’ actions remained the same as in Version 1. In Experime [file pcbi.1013032.s002.tif]

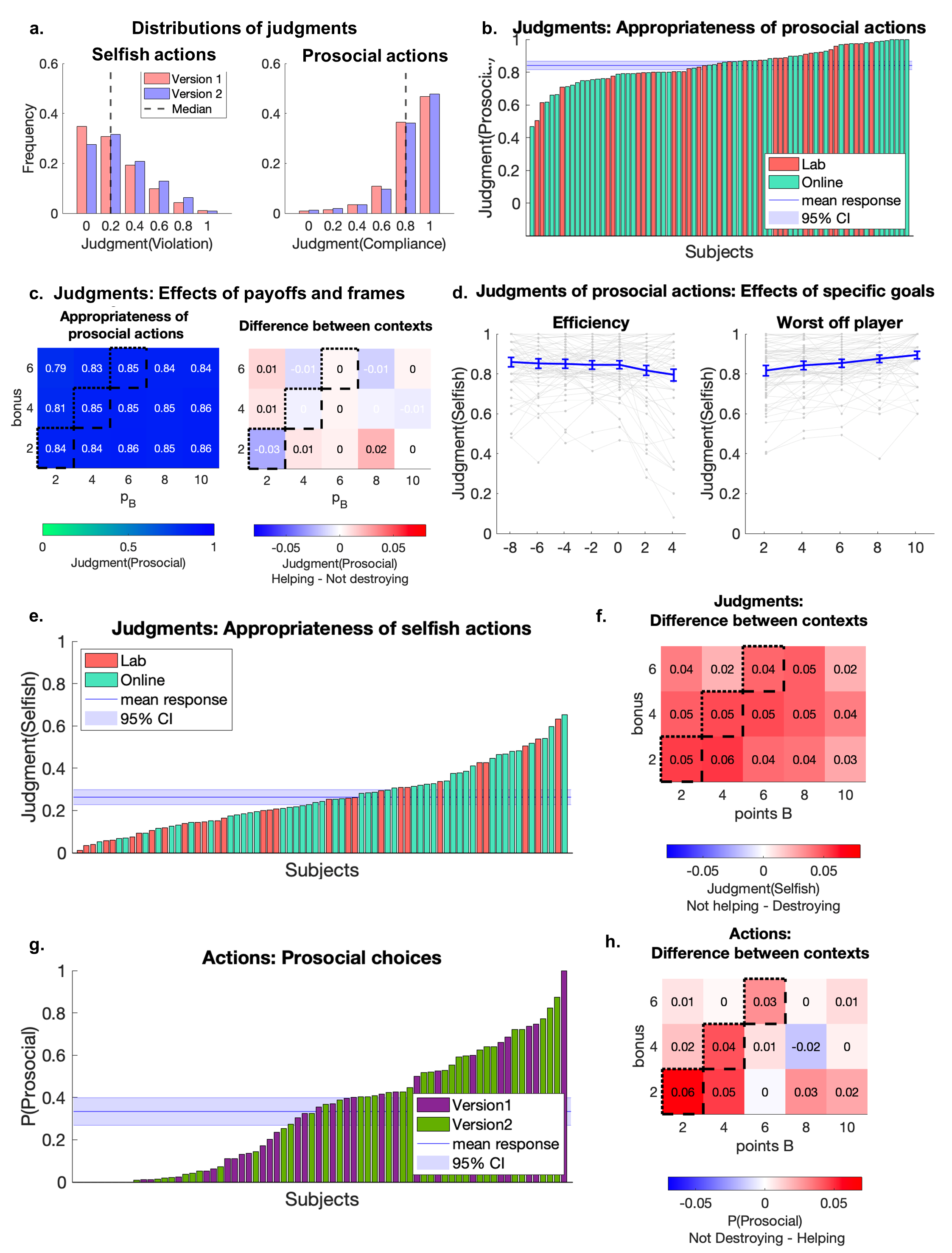

Supplement: S2 Fig — Supplement to Figs 1 and 2, judgment data from Experiment 1. (a) Distributions of ratings for selfish and prosocial actions, across all participants and trials of Experiment 1, showing low individual differences. All judgments were rescaled to 0–1 interval: 0 corresponds to very socially inappropriate and 1 to very socially appropriate. (b) Average appropriateness judgment of prosocial actions for each participant. The blue line and interval represent the mean and its confidence interval across participants. (c) Effect of payoff distributions on normative judgments of prosocial actions and on its difference between contexts. The color scales represent the average appropriateness judgment of trials during which A chose the prosocial action (left), or the difference in these ratings between the two contexts (right). The black lines represent efficiency thresholds: Selfish actions are efficient for trials above the dotted lines, and prosocial actions are efficient for trials below the dashed line. Trials between those two lines have equal efficiency for selfish and prosocial actions (pB = b). (d) Absence of effects of selfish action efficiency (b-pB) and score of the worst-off player (min (pA, pB)) on mean appropriateness judgment of prosocial actions. These results show the low inter-individual and inter-trial variations of judgments provided when Player A picked the prosocial action, showing that prosocial action is viewed as unanimously appropriate regardless of the specific situation. (e, g) Individual differences in judgments (rescaled to 0 (very socially inappropriate) - 1 (very socially appropriate)) for trials in which Player A picked the selfish action (e) or actions of Player A (g). Each bar represents the average rating of a participant of Experiment 1 (e) or the prosocial action rate of a participant of Experiment 2 (g); the different colors indicate which version of the experiment participants participated in (see S1 Text and S1 Fig). The blue line and int [file pcbi.1013032.s003.tif]

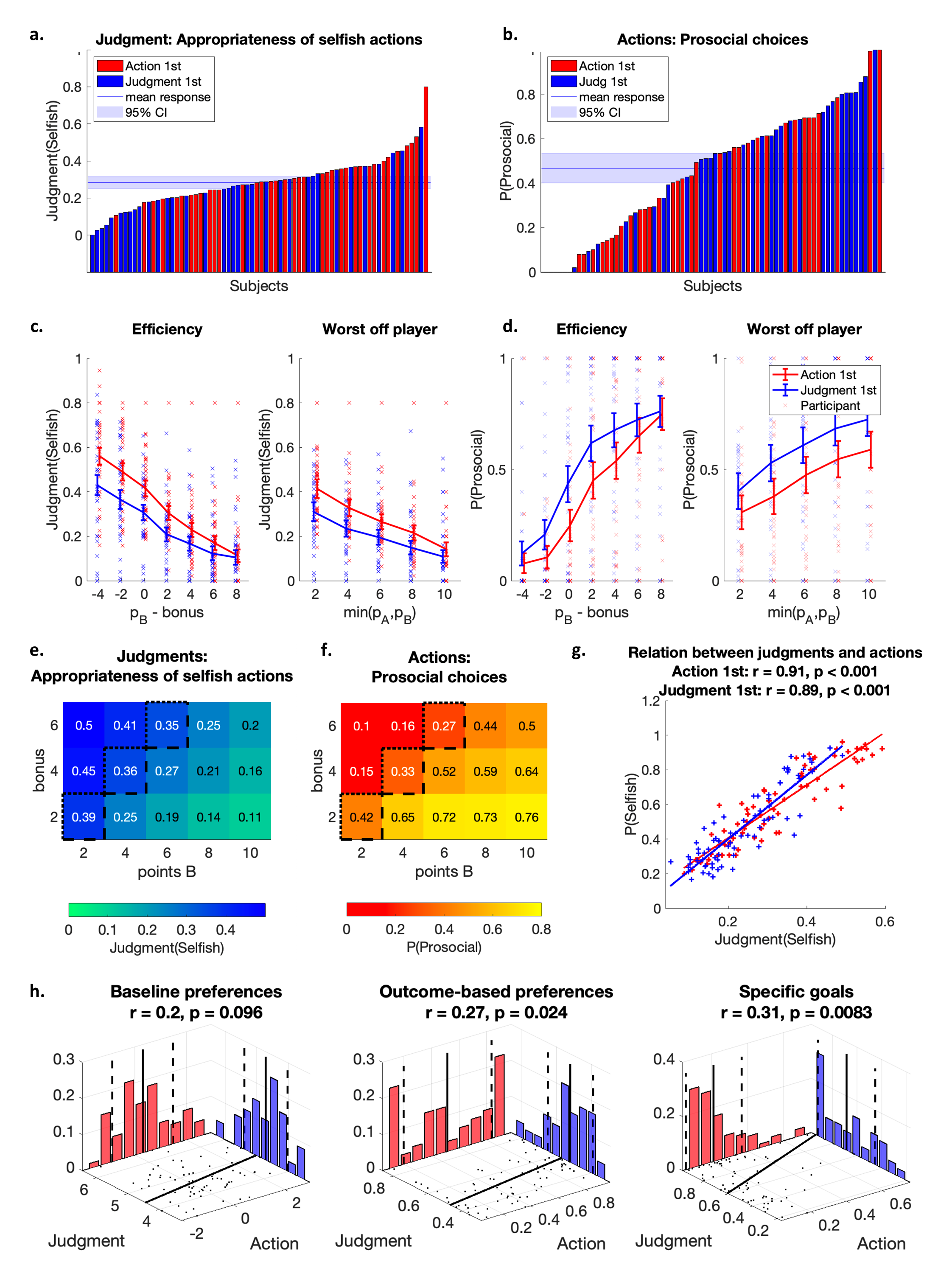

Supplement: S3 Fig — Supplement to Fig 2. (a) Individual differences in judgments of trials in which Player A picked the selfish action. Each bar represents the average rating of a participant (rescaled to 0 (very socially inappropriate) to 1 (very socially appropriate)). (b) Individual differences in prosocial actions. Each bar represents the prosocial action rate of a participant in the experiment. (a-b) The colors indicate whether participants played the action (red) or judgment (blue) task first. The blue line and interval represent the mean and its confidence interval across participants. (c, d) Effects of prosocial action Efficiency (pB - b) and score of the worst-off player (min (pA, pB)) on the mean appropriateness judgments of selfish actions rescaled to 0–1 interval (c) and prosocial action rate (d) for the two different task orders: Action 1st in red and judgment 1st in blue. Each cross represents a participant, the lines are the average across participants and error bars represent the standard error of the mean. Participants who participated in the judgment task 1st have an increased prosocial action rate, and stricter judgments compared to participants who started with the action task (S1 and S2 Tables), nonetheless, the effects of the different task parameters are comparable in these two groups. (e, f) Effect of payoff distributions (points of Player B and bonus) on average judgments (e) and prosocial action rate (f). The color scales represent the average behavior across participants. The black lines represent efficiency thresholds: Selfish actions are efficient for trials above the dotted lines, and prosocial actions are efficient for trials below the dashed line. Trials between those two lines have equal efficiency for selfish and prosocial actions (pB = b). selfish action increases with stakes (bonus) and decreases with potential harm caused (pB). Judgments become more lenient (towards appropriate) as stakes (bonus) increase and stricter (towards inappropriate) as the [file pcbi.1013032.s004.tif]

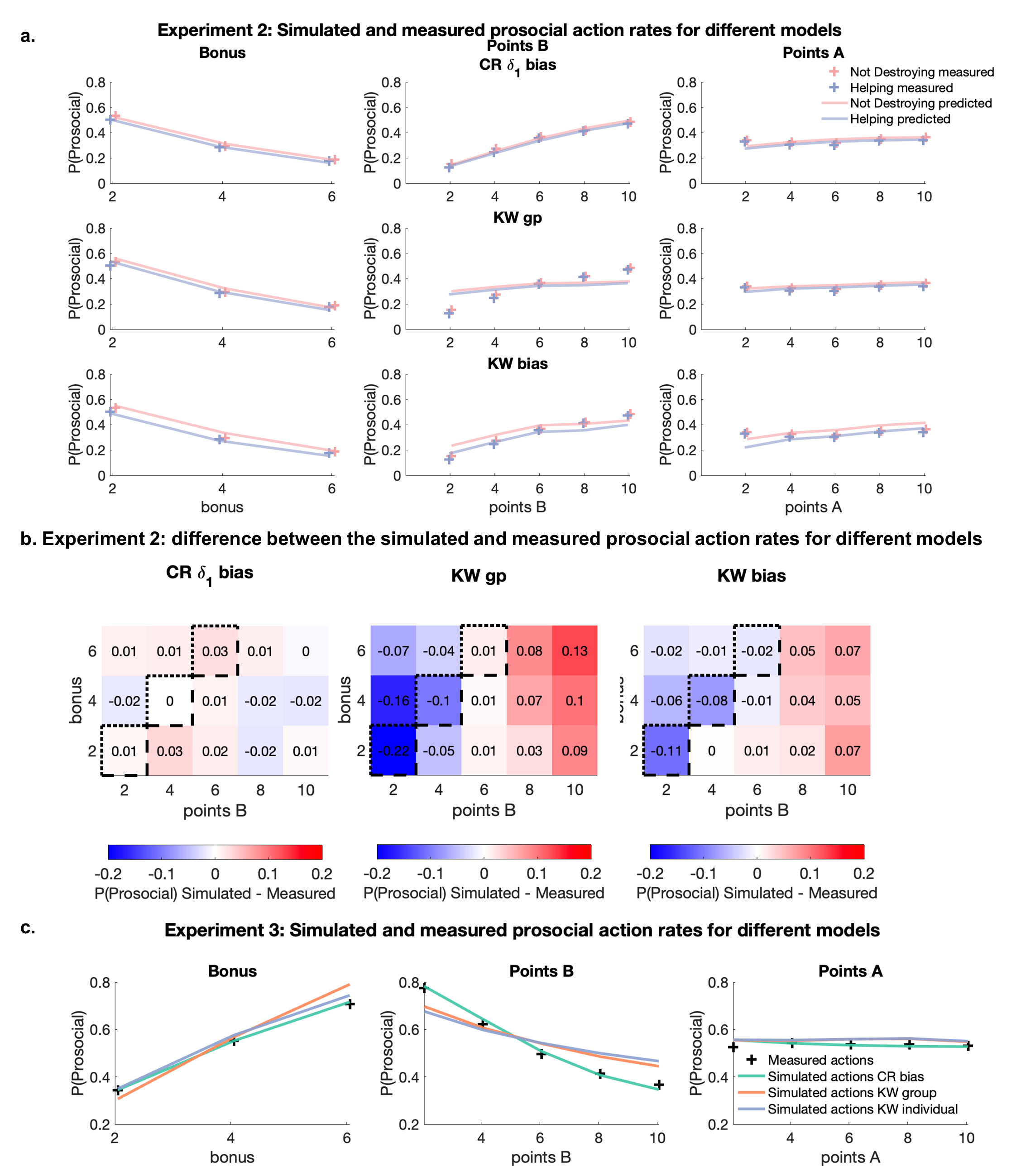

Supplement: S4 Fig — Supplement to Fig 2. (a) Experiment 2: Measured and predicted behavior using simulations of the different models (using the estimated parameters of each participant): Charness and Rabin model with a bias and discount factor (CR δ1 bias), Krupka and Weber model (KW), modeling actions using judgment data, and Krupka and Weber model including a bias (KW bias). The lines represent data simulated using the fitted model parameters of each participant. The crosses are the measured behavior. The colors correspond to the two contexts: Not destroying in pink and Helping in purple. The KW model failed to capture the effect of the points of Player B on prosocial actions, whereas the CR model best explained the participants’ choices. (b) Experiment 2: Difference between the behavior measured and simulated using the different models. The colors represent the difference in proportion of trials where Player A chose the prosocial actions. Blue colors represent a model underestimation of the prosocial decisions, and red colors a model overestimation of prosocial choices. Black lines represent efficiency thresholds: Selfish actions are efficient for trials above the dotted lines, and prosocial actions are efficient for trials below the dashed line. The Krupka and Weber models do not capture the effect of efficiency on the choices of Player A, while the Charness and Rabin model shows no systematic deviation from the measured behavior. (c) Experiment 3: Measured and predicted behavior using simulations of the different models: Charness and Rabin including a bias term (CR bias in green), Krupka and Weber using the average judgment across all participants (KW group in orange), or the judgment of the participant making the actions (KW individual in purple). The lines represent data simulated using the fitted model parameters of each participant and the crosses represent the measured behavior, showing again that the KW model failed to capture major trends in behavior. (TIF) [file pcbi.1013032.s005.tif]

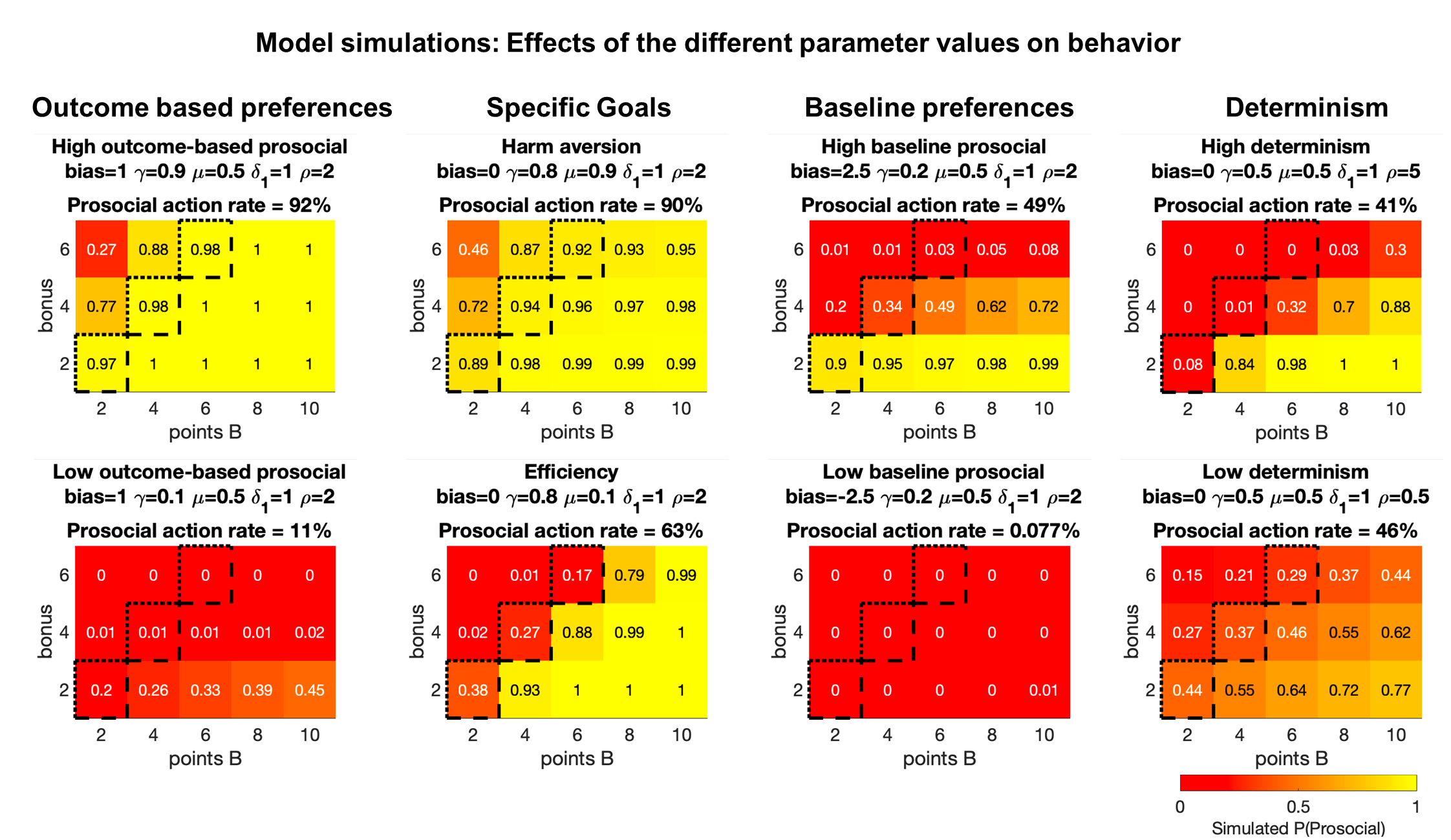

Supplement: S5 Fig — Simulations of the best model (CR δ1 bias) illustrate the effects of parameter variations on behavior. The color scales represent the predicted proportion of prosocial actions, the black lines represent efficiency thresholds: Selfish actions are efficient for trials above the dotted lines, and prosocial actions are efficient for trials below the dashed line. Trials between those two lines have equal efficiency for selfish and prosocial actions (pB = b). These simulations show that the different parameters model different aspects of behavior reflected in the influence of the payoff distributions on participants’ choices, which can be observed in the experimental population (see Fig 3d for the true behavior of different types of participants). (TIF) [file pcbi.1013032.s006.tif]

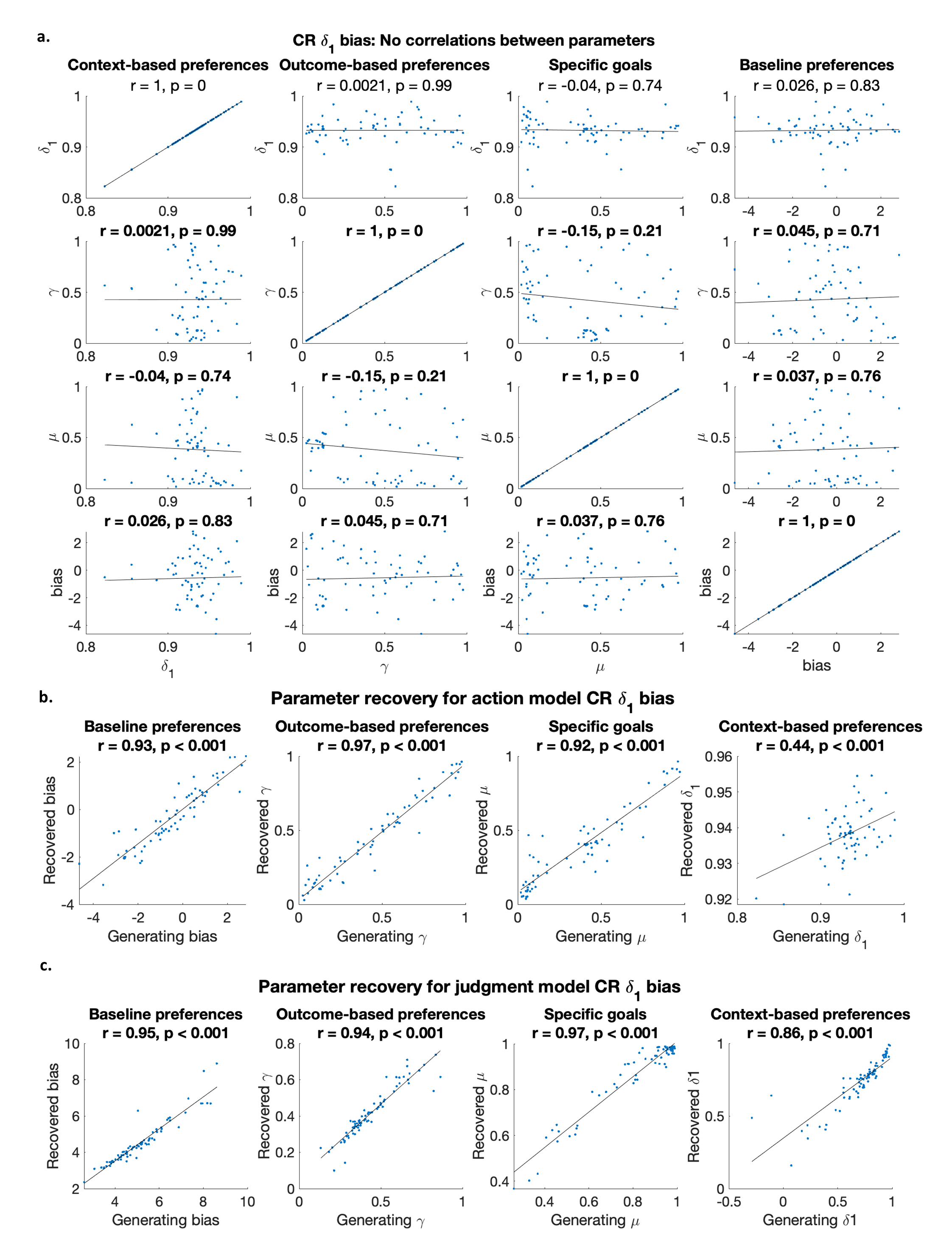

Supplement: S6 Fig — Supplement to Fig 2. (a) Relations between the four parameters of the winning model (CR δ1 bias): Context-based preferences (δ1), outcome-based preferences (γ), specific goals (μ), and baseline preferences (bias). Each dot represents the parameter of one participant. The black lines and coefficients represent regressions between the different parameters. None of the parameters are significantly related, showing that the model captures distinct aspects of behavior. (b-c). Parameter recovery action model (b) and judgment model (c). Generating parameters (empirical values fitted to the data of Experiments 2 (b) and 3 (c)) are used to simulate choices using the CR δ1 bias model. These choices are used to recover the generating parameters (using JAGS for hierarchical model fitting, as in the main analysis). Each dot plots the recovered parameter from the simulated behavior against the generating parameter, and the black lines represent the regression of the recovered on the generating parameters. All parameters recovered well, validating our modeling approach. (TIF) [file pcbi.1013032.s007.tif]

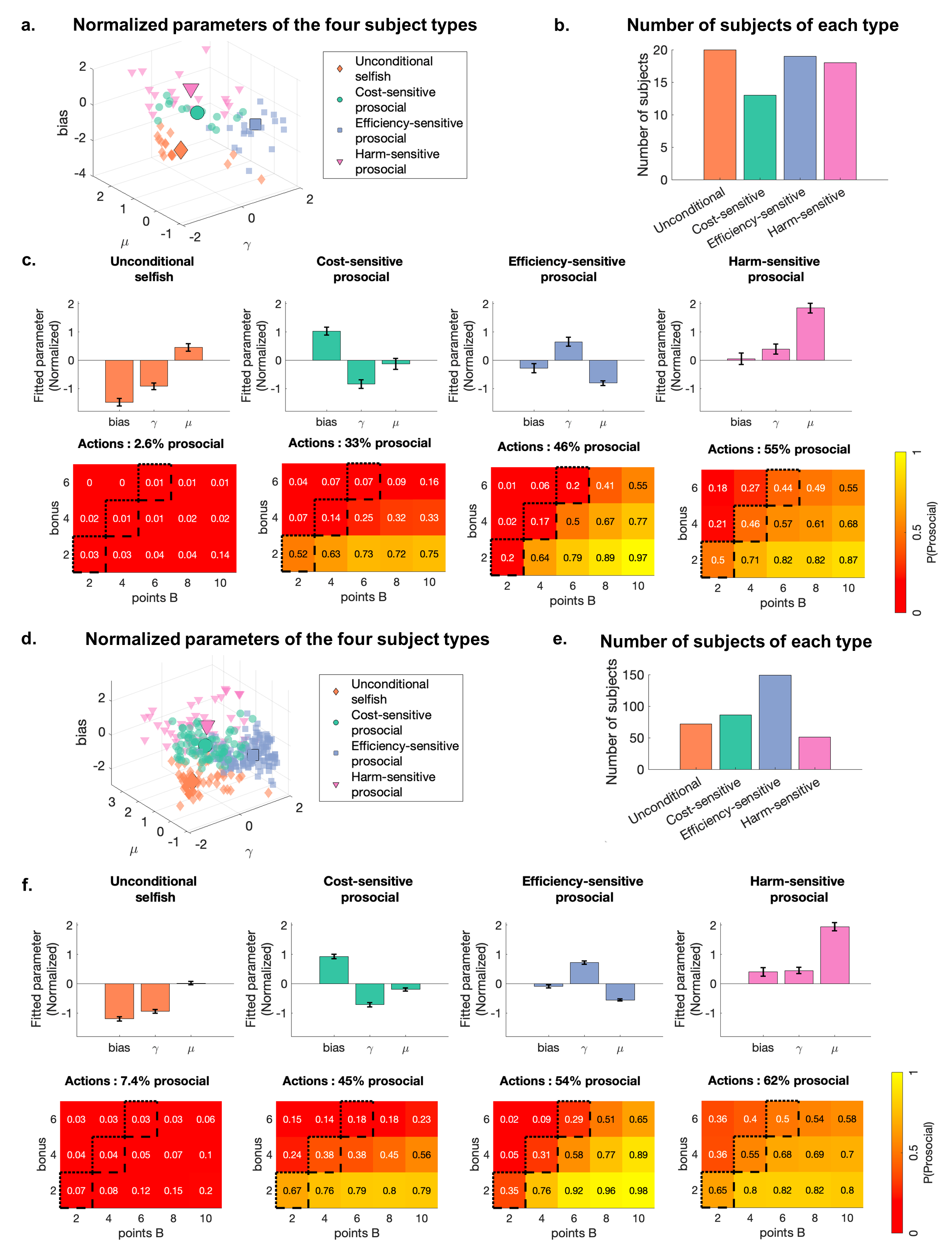

Supplement: S7 Fig — Supplement to Fig 3. (a and d) Parameter combinations of each participant of Experiment 2 (a) and Experiment 4 (d) and centroid of the 4 clusters resulting from the optimization procedure and training on Experiment 1 and 3 data. Four different types of participants are represented by different markers and colors. (b and e) Number of participants in each cluster, for Experiment 2 (b) and Experiment 4 (e). (c and f) Parameter distributions of the 4 clusters for participants of Experiment 2 (c) and Experiment 4 (f), effects of bonus and points of player B on prosocial actions averaged across participants in each of the four clusters. The colors represent the prosocial action rates. The black lines represent efficiency thresholds: Selfish actions are efficient for trials above the dotted lines, and prosocial actions are efficient for trials below the dashed line. Trials between those two lines have equal efficiency for selfish and prosocial actions (pB = b). Participants of the different clusters have different patterns of prosocial actions, reflecting the importance of parameters for their actions. The patterns of prosocial actions are similar across all experiments for the different clusters, confirming the relevance and stability of the clustering procedure. (TIF) [file pcbi.1013032.s008.tif]

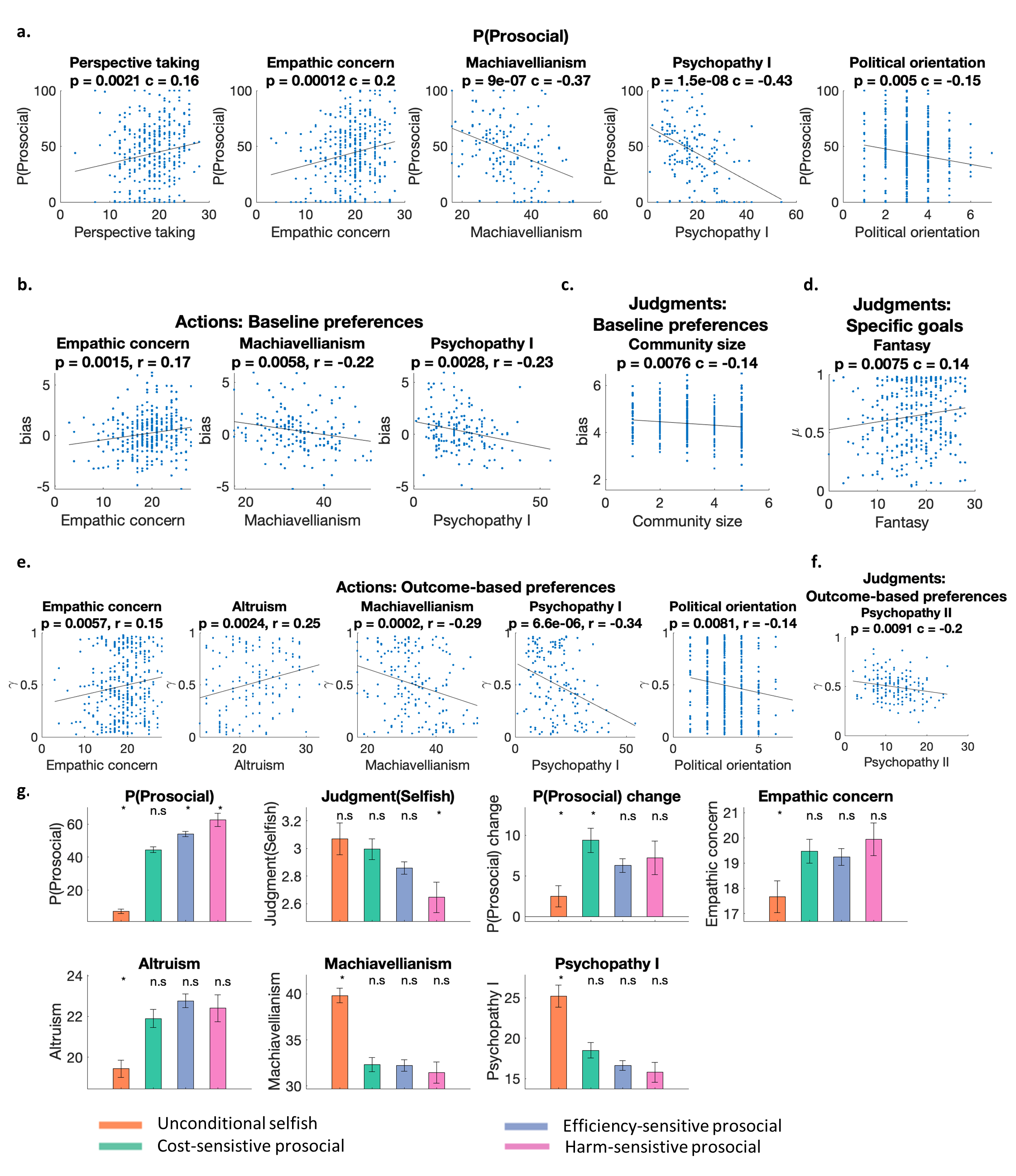

Supplement: S8 Fig — Supplement to the results section “Four types of individuals with distinct patterns of motives” (a) Statistically significant (using a Bonferroni correction P < 0.0083) Pearson’s correlations between self-reports and average behaviors in the action and judgment tasks. Each dot represents a participant of Experiments 3 and 4. The demographic and personality traits are measured using the questionnaires reported in S9 Table. No trait was significantly related to average judgments, while action rates correlated with perspective taking and empathic concern subscales of the IRI questionnaire, and were anticorrelated with primary psychopathy, Machiavellianism, and right-wing orientation. (b-f) Statistically significant (using a Bonferroni correction P < 0.0125) Pearson’s correlations between self-reports and fitted parameters of the action and judgment models. Each dot represents a participant of Experiments 3 and 4. (b) Action model, baseline preferences (bias). (c) Judgment model, baseline preferences (bias). (d) Judgment model, specific goals (μ). (e) Action model, outcome-based preferences (γ). (f) Judgment model, outcome-based preferences (γ). (g) Differences in task performance and self-reports of participants of the different clusters. Each bar is the mean score across participants of a cluster. The significance level corresponds to P -values < 0.125 (Bonferroni corrected) of post hoc two-tailed t-tests comparing the score of one versus all 3 other groups when the clusters have significant effects on the score (tested with ANOVA, P < 0.05). These results show that some traits such as psychopathy, Machiavellianism, or empathic concern are reflected in participants’ normative behaviors and related to specific actions and judgments concerns. The type of participant Unconditional Selfish has, in particular, high psychopathy and Machiavellianism and low empathy or altruistic scores. (TIF) [file pcbi.1013032.s009.tif]

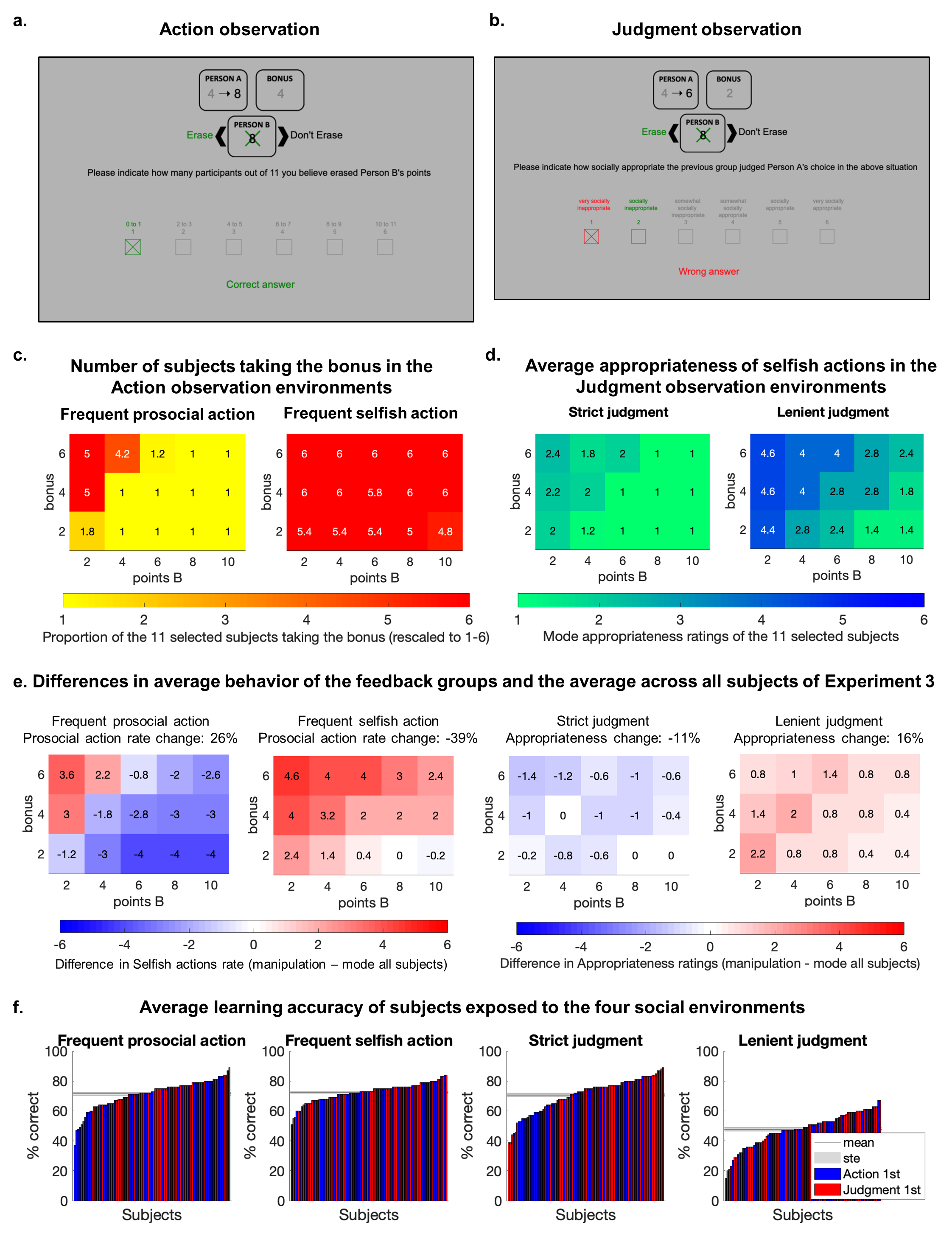

Supplement: S9 Fig — Supplement to Fig 4. (a) Exposure screen for the descriptive norms (observing actions). Participants must guess how many out of 11 previous participants decided to take the bonus and erase the points of Player B in the situation described. (b) Exposure screen for the prescriptive norms (observing judgments). Participants must guess the mode appropriateness rating of 11 previous participants judging the actions of a fictive Player A in the situation described. (c) Correct answers for the frequent prosocial action and frequent selfish action environments (descriptive norms). The color gradient shows the correct responses, corresponding to how many participants picked the selfish action in the different situations, as a function of the bonus and points of Player B. The correct responses were computed using the behavior of the most (resp. least) prosocial participants of Experiment 3 and rescaled to 1–6. (d) Correct answers for strict and lenient judgment environments (prescriptive norms). The color gradient shows the correct responses, corresponding to the mode appropriateness ratings of 11 previous participants in the different situations, as a function of the bonus and points of Player B. The correct response was computed using the mode behavior of the participants of Experiment 3 judging selfish action as the most (resp. least) inappropriate on average. (e) Percentage difference in the average actions or judgments between the four selected groups and the average of all participants of Experiment 3, for the different values of the bonus and points of Player B. The colors represent the difference between the group and overall mode behavior. (f) Learning performance. Percentage of correct answers during the exposure phase for each environment. Each bar represents a participant. The colors represent the task orders (action 1st in blue and judgment 1st in red). The grey line and shaded areas are the mean and standard error across participants. This figure shows the diffe [file pcbi.1013032.s010.tif]

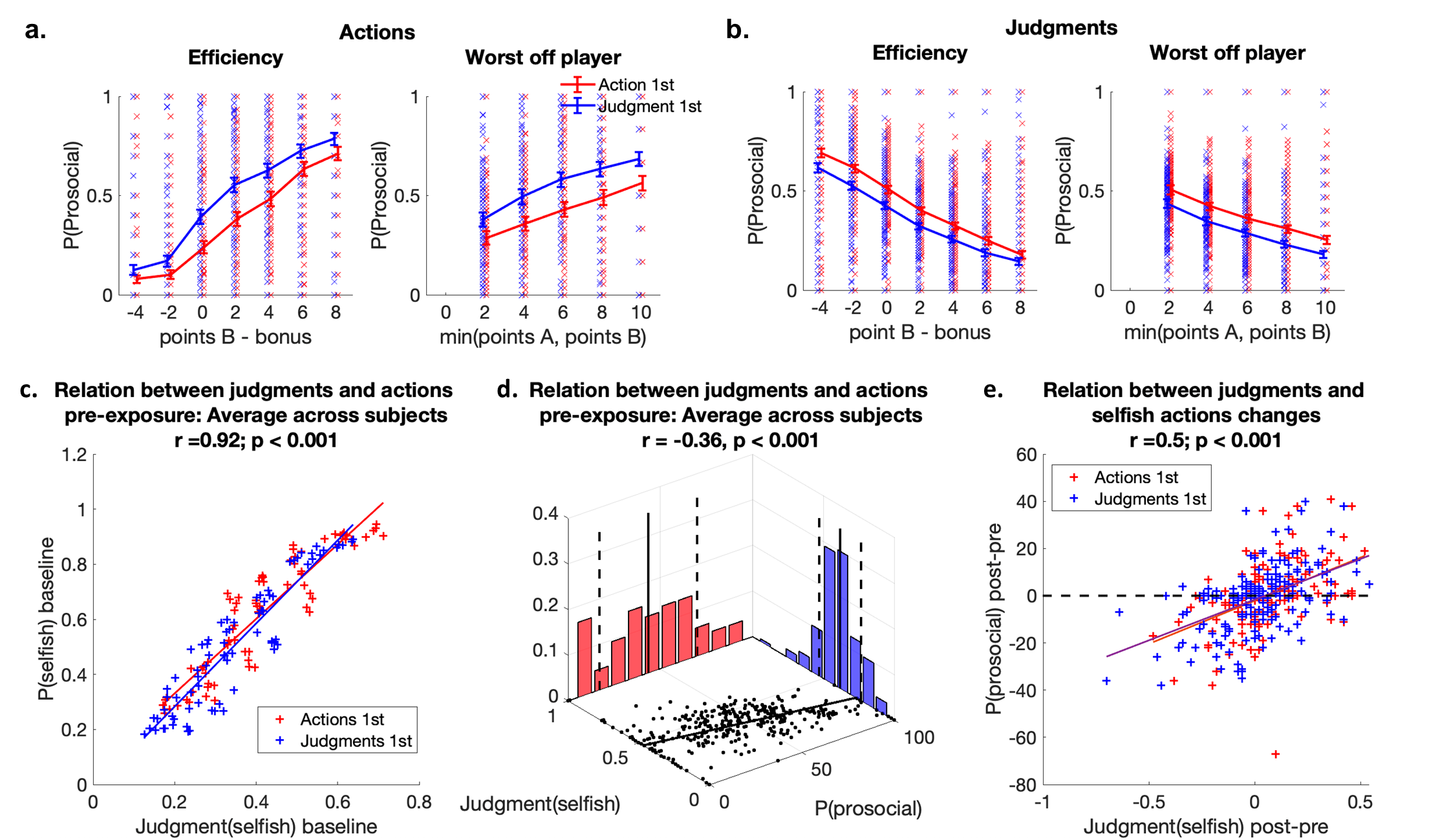

Supplement: S10 Fig — Supplement to Fig 4. (a and b) Effects of prosocial action efficiency (pB-b) and score of the worst-off player (min (pA, pB)) on the proportion of prosocial action trials (a) and mean appropriateness judgment of selfish action trials rescaled to 0–1 interval (b). For the two different task orders: Action 1st in red and judgment 1st in blue. Each cross represents a participant, the lines are the average across participants and error bars represent the standard error of the mean. (c) Correlation between selfish action rates and appropriateness judgments averaged across participants of Experiment 4, for the 75 different trials. The colors represent the task order: Action 1st in red and judgment 1st in blue. Each dot represents the results of one trial, averaged across all participants. The lines show regressions between selfish actions and judgments. Judgments averaged across participants are strongly correlated with the average selfish action rate, as reported in Experiments 1,2 and 3. (d) Bivariate distribution of the prosocial action rates (left), and mean appropriateness judgments (right), for each participant of Experiment 4. The mean and standard deviation across participants are represented in plain (resp. dotted) lines and show wide individual differences in prosocial action, and relative consensus of appropriateness judgments, as noted in Experiment 3. The black crosses represent each participant and show a small correlation between the average actions and judgments of the different participants. Although significant, this correlation is much smaller than the correlation at the group level (c), showing that average judgments have low predictive power on individuals’ prosocial actions. (e) Relation between prosocial action and judgment changes post- vs pre-exposure, showing a small effect. Participants who adapted their judgments the most after the exposure also had a higher change in their prosocial action rate. (TIF) [file pcbi.1013032.s011.tif]

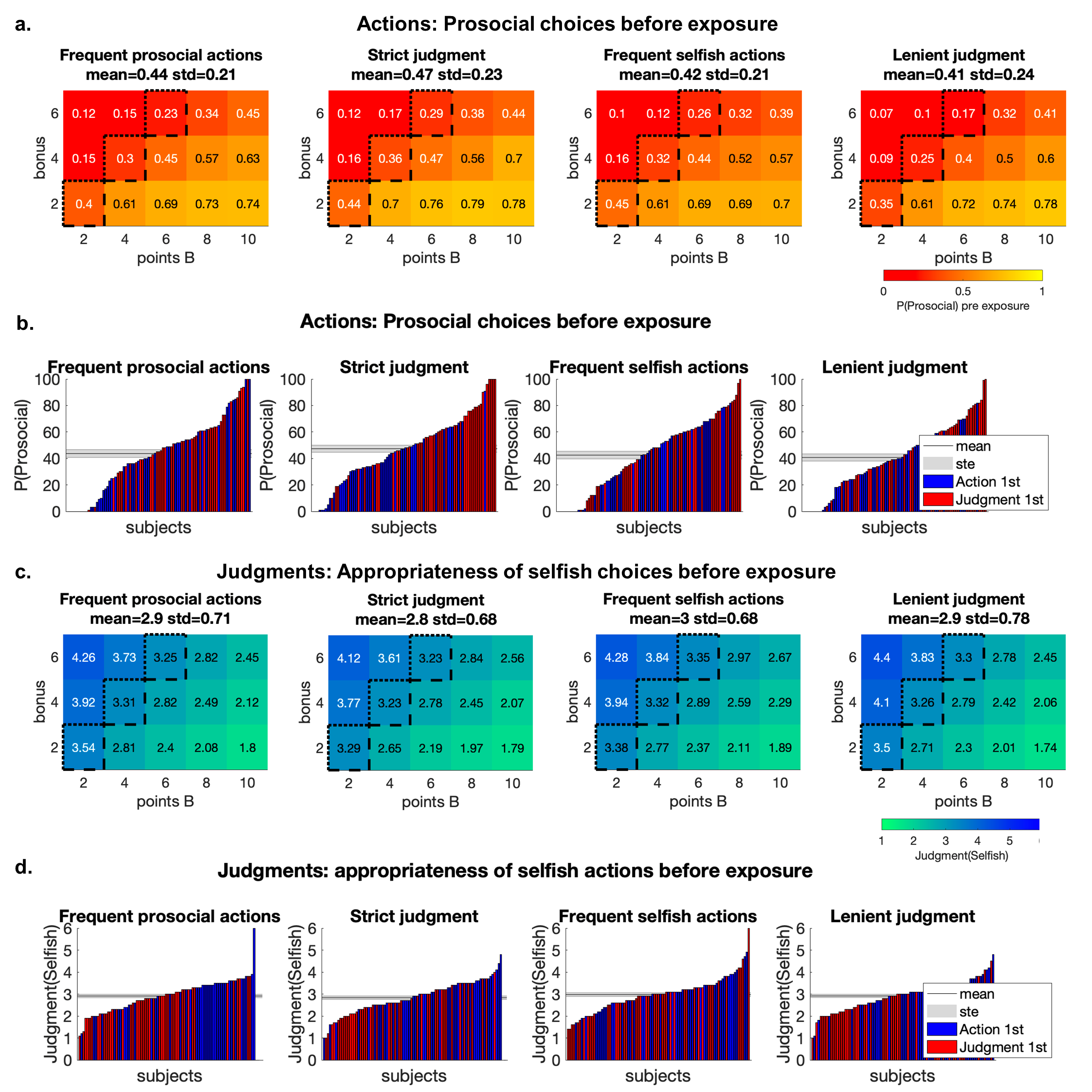

Supplement: S11 Fig — (a and c) Effect of payoff distributions (points of Player B and bonus) on prosocial action frequency (a) and average judgments (c) for the 4 groups, before the exposure phase. The color scales represent the average behavior across participants. (b) Individual differences in prosocial action for the 4 groups, before the exposure phase. Each bar represents the prosocial action rate of a participant before the exposure phase, the colors indicate whether participants played the action (red) or judgment (blue) task first. The blue line and interval represent the mean and its confidence interval across participants. (d) Individual differences in normative judgments for the 4 groups, before the exposure phase. Each bar represents the average rating of a participant (rescaled to 0 (very socially inappropriate) to 1 (very socially appropriate)). These results show that participants’ behavior in the four different experimental groups is comparable before the exposure phase, allowing us to compare the effects of the different types of exposure. (TIF) [file pcbi.1013032.s012.tif]

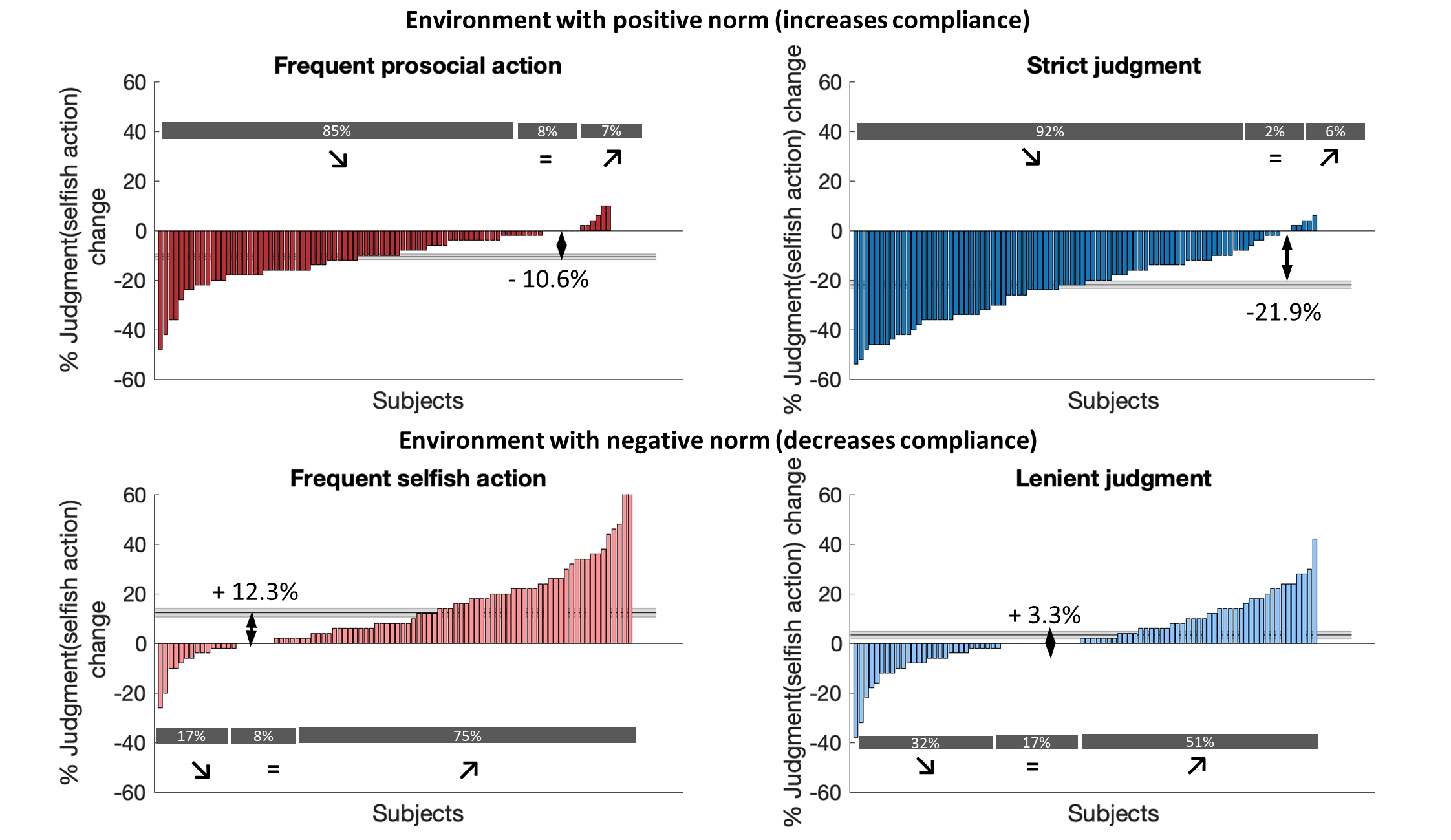

Supplement: S12 Fig — Supplement to Fig 4. Individual differences in responses to the four environments. The differences in judgments post- vs pre-exposure are displayed for each participant. The shaded grey line represents the mean and standard error across participants. The grey boxes show how many participants showed an increase, decrease, or no change in their judgments. Except for the lenient judgment exposure, most participants adapted their judgments in the predicted direction. (TIF) [file pcbi.1013032.s013.tif]

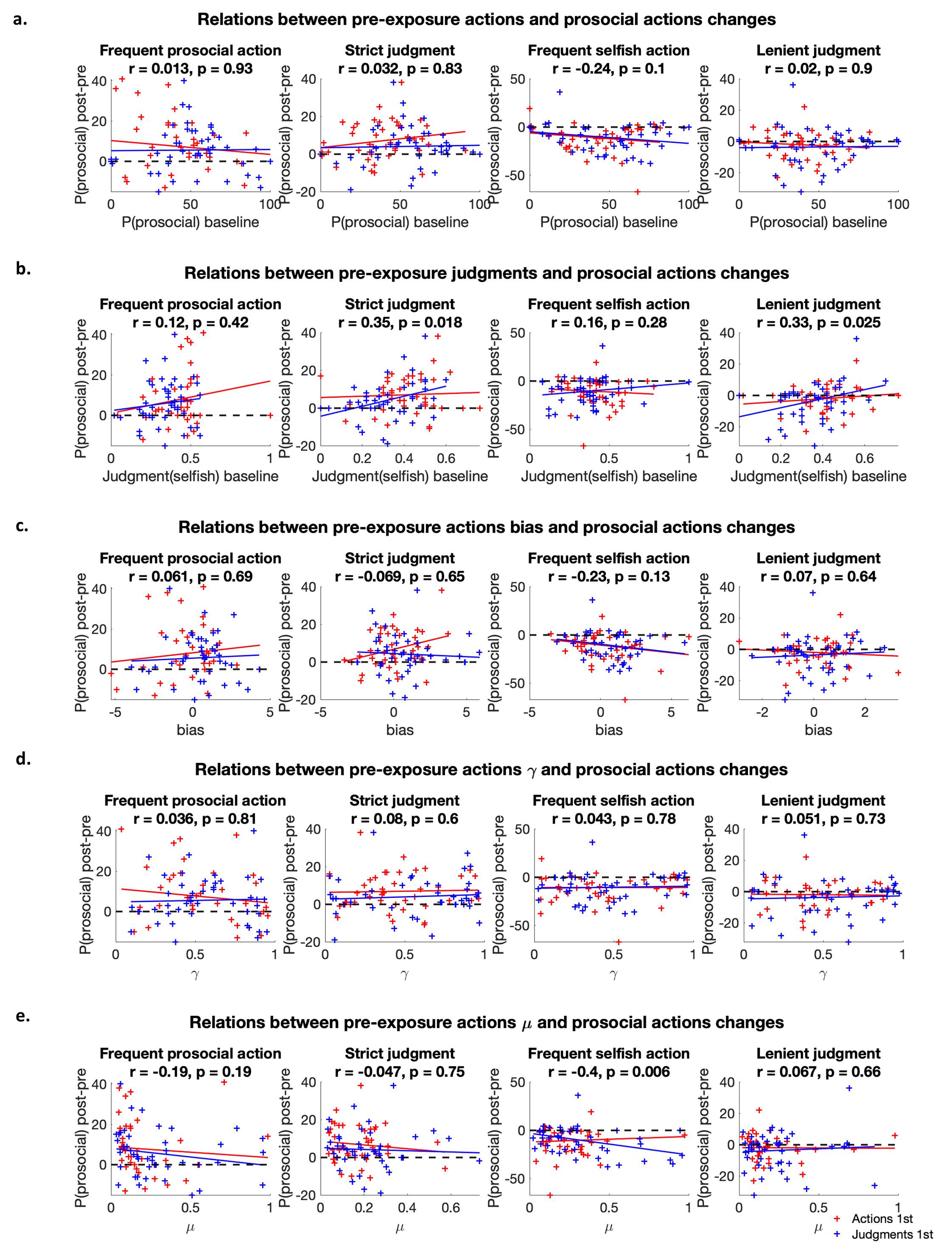

Supplement: S13 Fig — Supplement to Fig 4. (a and b): Relations between pre-exposure behavior and action change post vs pre-exposure. Each dot represents a participant and the colors represent the task orders. (a) Action: Absence of effects of the pre-exposure prosocial action rates on action changes post vs pre-exposure. (b) Judgments: Participants exposed to prescriptive norms (guessing the judgments of previous participants) had a significant correlation between their baseline judgments and prosocial action changes. For participants seeing strict judgments: The more lenient their baseline judgments were, the more their prosocial action increased after the exposure. Participants who were far off from the strict definition of the norm changed the most. In the lenient judgment environment, the stricter participants were in their baseline judgment (the further off from the lenient normative environment), the more their prosocial action decreased. Participants who had a perception of the prescriptive norm far from the one displayed during the exposure phase changed their behavior the most. These effects are however small, showing that the changes in prosocial action are mostly independent of the aggregate prosocial action or judgments pre-exposure. (c-e) Relations between the pre-exposure parameters of the CR bias model and action changes. Bonferroni-corrected correlations show no significant relation (all p > 0.004) between individual parameters and change in behavior. Each dot represents a participant, colors represent the order of the two tasks and the lines regressions between the two variables. (TIF) [file pcbi.1013032.s014.tif]

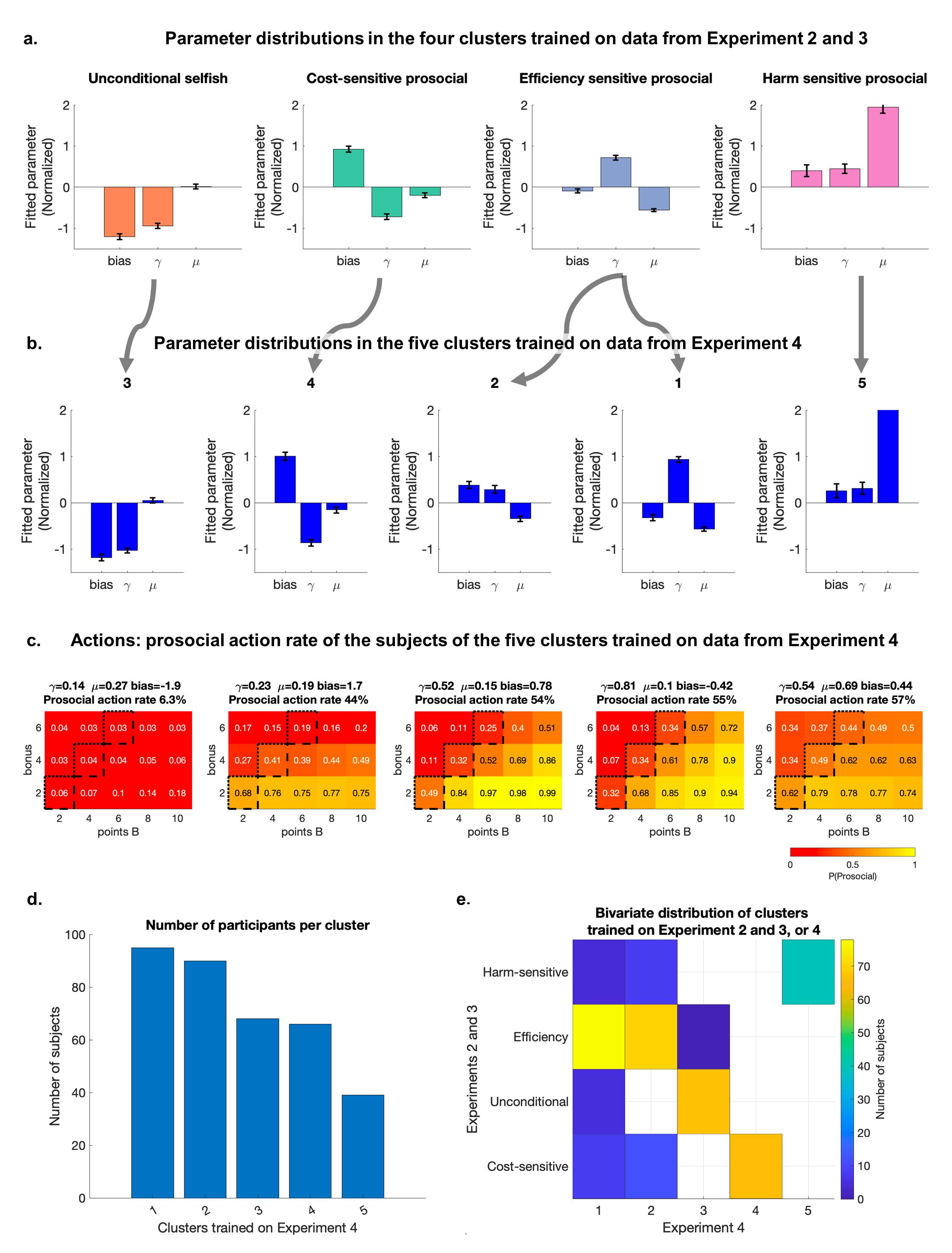

Supplement: S14 Fig — Supplement to Fig 3. The clustering algorithm training and optimization of the number of clusters was repeated using Experiment 4 only. (a) Parameter distributions of participants of Experiment 4 clustered using the clusters trained on data from Experiments 1 and 3. (b) Parameter values in the 5 new clusters found when repeating the clustering procedure using data from Experiment 4 only. Some clusters were equivalent to the previously defined clusters, while the Efficiency-sensitive cluster was separated into two clusters, that differed in their bias values. (c) Prosocial choices of the 5 newly defined clusters, with varying points of B and bonus. Most clusters had a similar pattern as the ones described in Fig 4. The two new sub-clusters containing the previous Efficiency-sensitive participants showed similar responses to variations of the bonus values and points of Player B, as well as a similar overall prosocial action rate. (d) Number of participants in each of the 5 newly defined clusters. (e) Bivariate distribution of participants of Experiment 4, across clusters defined in Experiments 1 and 3 (old clusters) and clusters defined in Experiment 4 (new clusters). Most participants that belonged to an « old cluster » are grouped in the same « new cluster », showing the robustness of the clustering procedure: Similar clusters are produced when training the algorithm using different sets of participants. As shown in (b) participants belonging to the Efficiency-sensitive cluster were separated into two distinct clusters (1 and 2) using this new data set. These results show the stability of our clustering approach, as replicating the procedure with a new data set generated similar clusters. (TIF) [file pcbi.1013032.s015.tif]

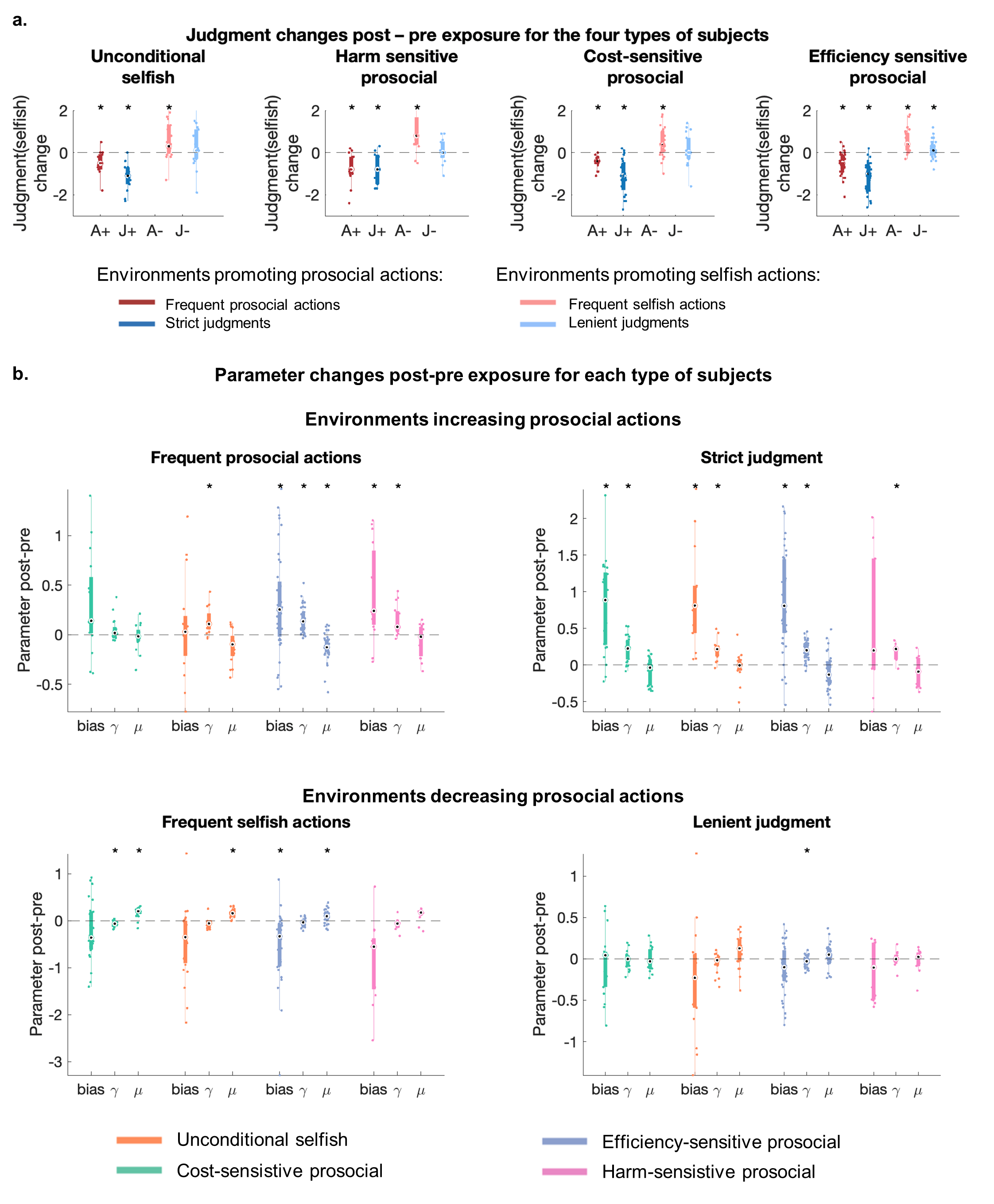

Supplement: S15 Fig — Supplement to Fig 4. (a) Post vs pre-appropriateness judgments of selfish actions for the 4 different types of environments. Participants of Experiment 4 were clustered into four groups, based on the baseline parameters of the action model. The difference in judgments before and after the exposure is displayed for each cluster. The colors represent the 4 different environments: A+ (Action + , frequent prosocial action) and J+ (Judgment + , strict judgment) are normative environments increasing prosocial actions, and A- (action -, frequent selfish action) and J- (Judgment -, lenient judgments) normative environments decreasing prosocial actions. Each dot represents a participant. The box overlay represents the median and 25% and 75% quartiles. Most individuals adapt their judgments after exposure. The changes in the four groups are comparable. (b) Judgment parameter changes following exposure. The CR model predicting judgments was modified to include parameter changes, that represent how much each parameter evolved after exposure. The colors represent the 4 different clusters, and each parameter change (baseline preferences (bias), outcome-based preferences (γ), and specific goals (μ)) is displayed for each cluster and environment. Each dot represents a participant. The box overlay represents the median and 25% and 75% quartiles. The patterns and magnitude of parameter changes are similar for the different clusters within each normative environment, although some parameter changes are below significance. (TIF) [file pcbi.1013032.s016.tif]

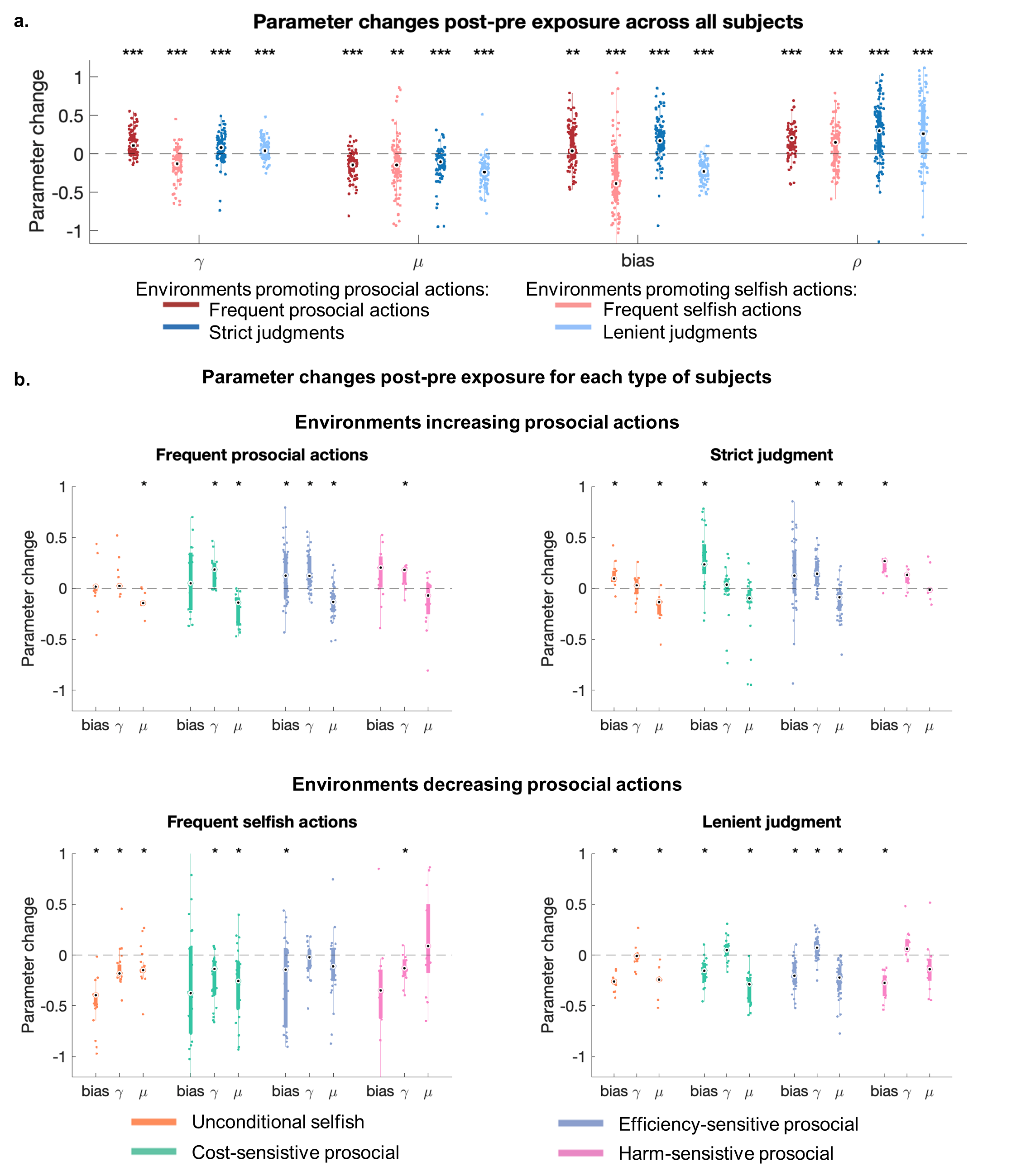

Supplement: S16 Fig — Supplement to Fig 4. Action model (CR bias) parameter changes following exposures. The CR bias model was modified to include a measure of parameter changes, that represent how much each parameter evolved after exposure. (a) Parameter changes across all participants, the colors represent the four environments and (b) parameter changes for each cluster, the colors represent the 4 different clusters. Each parameter change (baseline preferences (bias), outcome-based preferences (γ), and specific goals (μ)) is displayed for each cluster and environment. Each dot represents a participant. The box overlay represents the median and 25% and 75% quartiles. This figure shows that distinct types of participants show different patterns of change. See S15 Table for an Analysis of variance (ANOVA) of the effects of the types of participants (cluster), type of environment (descriptive versus prescriptive norms), and direction (positive versus negative changes) on the average changes in parameters. (TIF) [file pcbi.1013032.s017.tif]

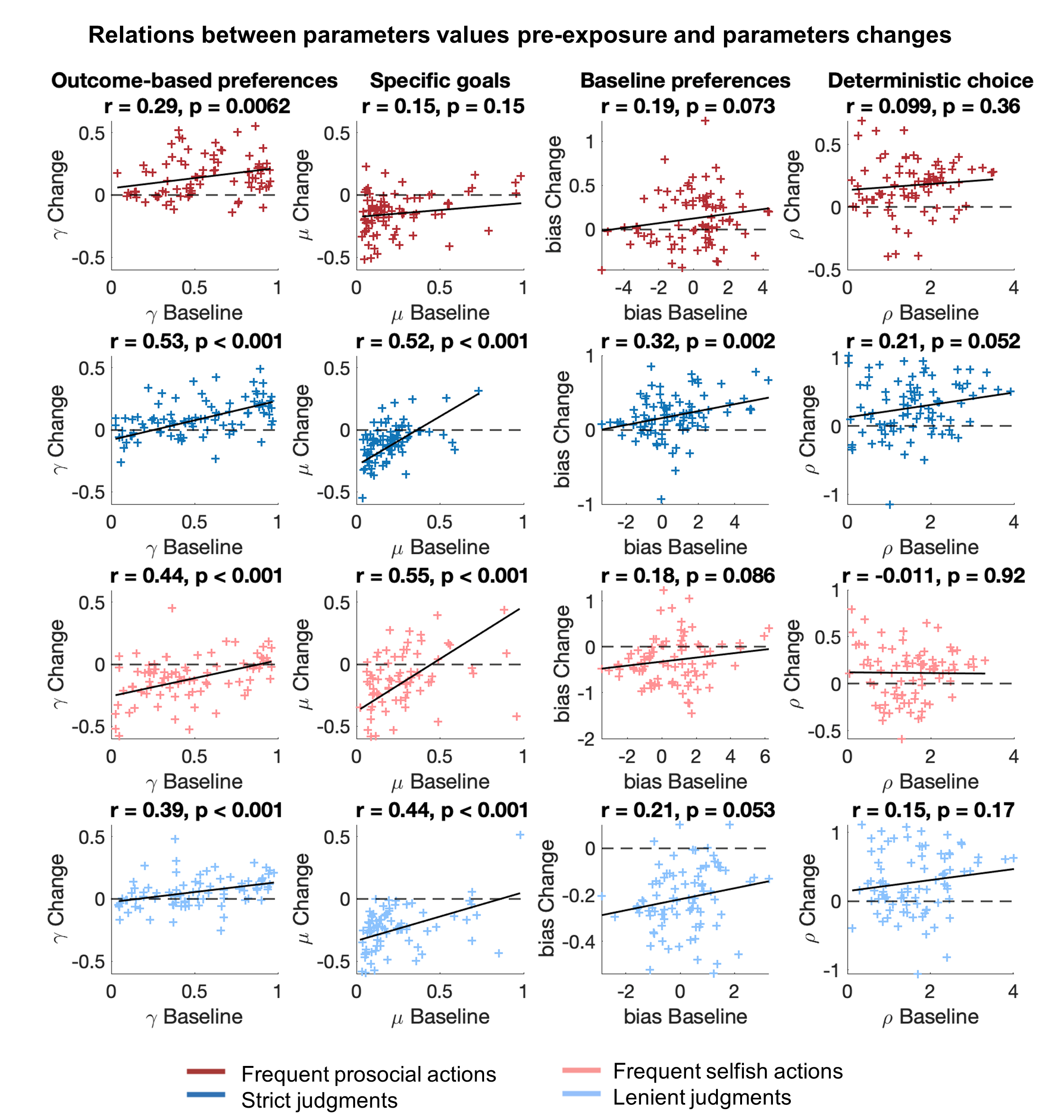

Supplement: S17 Fig — Supplement to Fig 4. Each dot represents the parameters of one participant. The colors represent the different normative environments. For environments increasing prosocial action, the higher the baseline outcome-based preferences (high γ), the more this parameter increases after the exposure. Participants sensitive to prosocial outcomes are inclined to become more sensitive to prosocial outcomes. Similarly, the lower the baseline outcome-based preferences, the more selfish participants become when observing frequent selfish actions. Participants observing lenient judgment mostly increase their outcome-based preferences, the higher the parameter, the more this parameter increases. For most environments (except observing frequent prosocial action), participants that have a baseline behavioral goal of efficiency (low μ) increased their efficiency concern even further. Pre-manipulation baseline preferences (bias) only significantly correlated with a bias increase when participants observed strict judgments: The more prosocially biased they were, the more this bias increased. The general trend shows that the more extreme participants’ preferences are, the more exposure to environments in agreement with these preferences reinforces them. (TIF) [file pcbi.1013032.s018.tif]

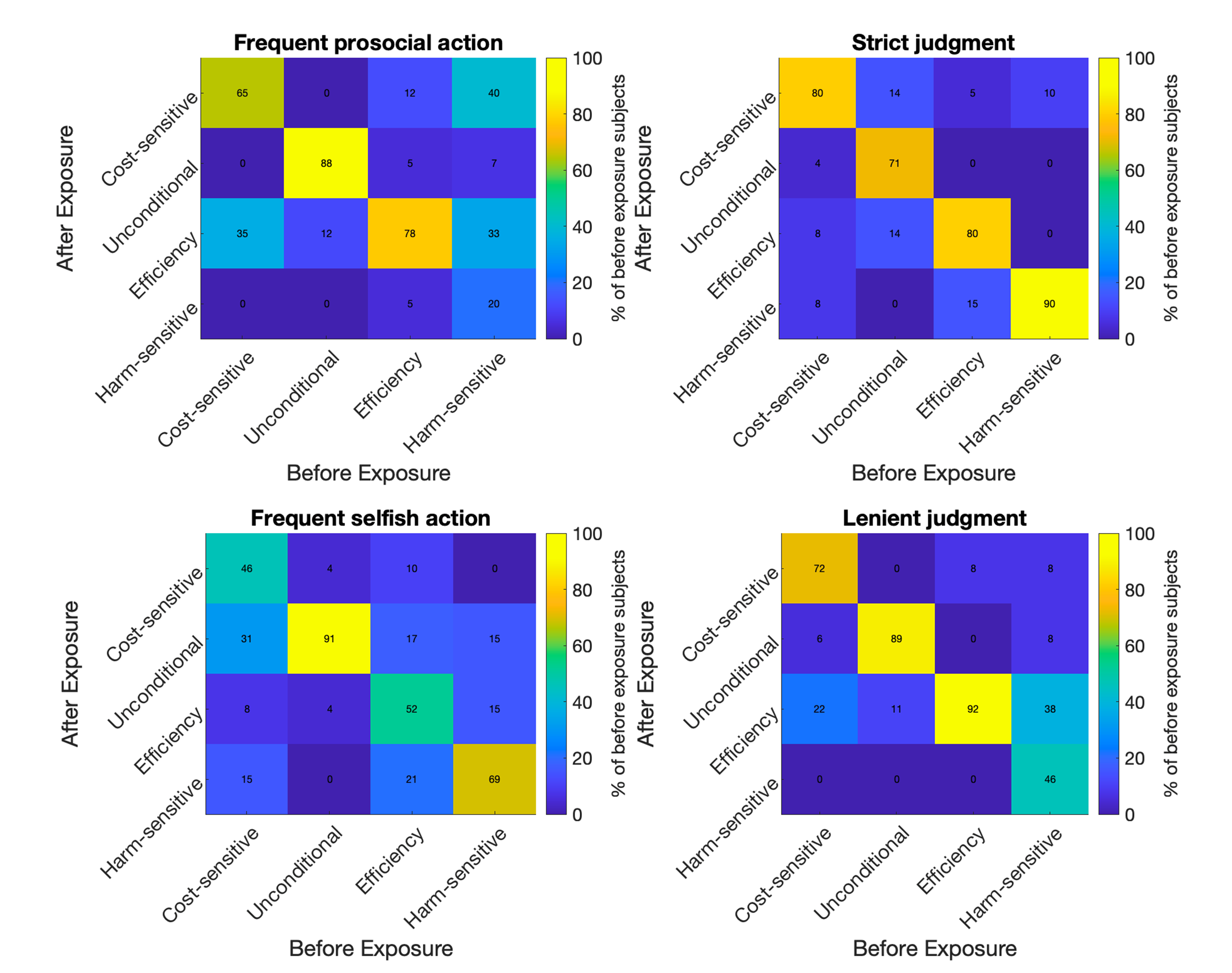

Supplement: S18 Fig — Participants of experiment 4 were clustered again based on the result of a CR bias model fitted to their post-exposure decisions. The pre- and post- exposure clusters were compared. The colors and numbers represent the percentage of participants of each pre-exposure cluster being clustered in each of the 4 types post exposure, for the 4 different environments. Most participants remained in the same cluster, showing relative stability of the motivational profiles. However, there were some systematic changes across the minority of people who shifted their motivational profile in response to the change in environment. Unconditional selfish mostly remained Unconditional selfish regardless of the environment; only a minority of them shifted towards a Cost-sensitive or Efficiency sensitive profile when observing strict judgments. A third of Cost-sensitive participants shifted their motivations towards efficiency when witnessing frequent prosocial actions, but also lenient judgments, while a third of them became Unconditional selfish when witnessing frequent selfish actions. The Efficiency-sensitive type was mostly stable, but did shift to Unconditional selfish or Harm-sensitive types when witnessing frequent selfish actions, showing that the response of these participants to this environment could strongly vary. Finally, Harm-sensitive participants changed types the most often and shifted towards more mitigated strategies (Cost- or Efficiency- sensitive) when witnessing prosocial actions, or even to Unconditional selfish when witnessing frequent selfish actions. (TIF) [file pcbi.1013032.s019.tif]
