# Supplementary material for: A computational account of multiple motives guiding context-dependent prosocial behavior
Source: PLoS Comput Biol. 2025 Apr 21;21(4):e1013032. doi: 10.1371/journal.pcbi.1013032 (PMC12112419; doi:10.1371/journal.pcbi.1013032)
Supplement: S1 Table — Fixed effects coefficient estimates, standard errors, and p-values of the judgment regressions mixed-effects models using participants as random effects. The judgment data were analyzed using a Cumulative Link Mixed Model. The judgments were analyzed separately for trials in which the fictive Player A chose the prosocial (a) versus selfish action (b). Due to model convergence issues, only the effect of the context, and bonus (for selfish trials) were included as a random slope for each participant. Models including any of the points of A, points of B, or bonus (for prosocial trials only), as random slopes failed to converge due to a sample size too small to estimate such effects for ordinal data. Judgments from Experiments 1 and 3 were used, showing no significant difference between experiments (P > 0.27). The last model (Experiment 1 and 3 demographics) includes self-reported demographic variables (See S9 Table) and shows a significant effect of family affluence and weekly spending money on judgments of selfish actions and of age and religion on judgments of prosocial action. Model comparison showed a significant improvement in model fits when adding the demographic variables for selfish actions (ANOVA: P = 0.005) but not prosocial actions (P = 0.32). However, the model improvement was small and did not alter the main coefficients. These statistics show that the judgments of selfish actions strongly depended on the specific situations (context, points of A and B and bonus, see Figs 2 and S3), while the judgments of prosocial actions only moderately varied across situations (S2 Fig). (DOCX) [file pcbi.1013032.s020.docx]

**S1 Table.** **Statistical analysis – Judgments Experiment 1 and 3 main effects.** Fixed effects coefficient estimates, standard errors, and p-values of the judgment regressions mixed-effects models using participants as random effects. The judgment data were analyzed using a Cumulative Link Mixed Model. The judgments were analyzed separately for trials in which the fictive player chose the prosocial (a) versus selfish action (b). Due to model convergence issues, only the effect of the context, and bonus (for selfish trials) were included as a random slope for each participant. Models including any of the points of A, points of B, or bonus (for prosocial trials only), as random slopes failed to converge due to a sample size too small to estimate such effects for ordinal data. Judgments from Experiments 1 and 3 were used, showing no significant difference between experiments (*P* > 0.27). The last model (Experiment 1 and 3 demographics) includes self-reported demographic variables (See S9 Table) and shows a significant effect of family affluence and weekly spending money on judgments of selfish actions and of age and religion on judgments of prosocial action. Model comparison showed a significant improvement in model fits when adding the demographic variables for selfish actions (ANOVA: *P* = 0.005) but not prosocial actions (*P* = 0.32). However, the model improvement was small and did not alter the main coefficients. These statistics show that the judgments of selfish actions strongly depended on the specific situations (context, points of A and B and bonus, see Fig 2 and S3 Fig), while the judgments of prosocial actions only moderately varied across situations (S2 Fig).

1. Trials in which A picked the prosocial action (refused the bonus)

$$Judgment\left( Prosocial action \right)\sim Context + Bonus + Points A + Points B + Version+Task order+Experiment+ \left( 1+Context \right| Subject)$$

|  | **Experiment 1** | **Experiment 3** | **Experiments 1 and 3** | **Experiments 1 and 3, demographics** | | |
| --- | --- | --- | --- | --- | --- | --- |
| Context | 0.06 |  | 0.05 | | 0.07 | |
|  | (0.06) |  | (0.07) | | (0.07) | |
| **Bonus** | **-0.47 ***** | **-0.29** | **-0.42 ***** | | **-0.42 ***** | |
|  | (0.13) | (0.20) | (0.11) | | (0.11) | |
| **Points A** | **0.42 ***** | **-0.05** | **0.29 ***** | | **0.29 ***** | |
|  | (0.07) | (0.12) | (0.06) | | (0.06) | |
| **Points B** | **0.71 ***** | **1.77 ***** | **0.99 ***** | | **0.99 ***** | |
|  | (0.07) | (0.12) | (0.06) | | (0.06) | |
| Version | 0.09 |  | 0.11 | | -0.85 | |
|  | (0.48) |  | (0.53) | | (0.71) | |
| Task order |  | -0.35 | -0.32 | | -0.58 | |
|  |  | (0.63) | (0.55) | | (0.54) | |
| Experiment |  |  | 0.55 | | -0.37 | |
|  |  |  | (0.52) | | (2.30) | |
| Age |  |  |  | | -0.12 | |
|  |  |  |  | | (0.08) | |
| Gender (Female) |  |  |  | | -1.63 | |
|  |  |  |  | | (2.19) | |
| Gender (Male) |  |  |  | | -1.95 | |
|  |  |  |  | | (2.26) | |
| Politics |  |  |  | | -1.59 | |
|  |  |  |  | | (1.21) | |
| Community size |  |  |  | | -0.11 | |
|  |  |  |  | | (0.75) | |
| Affluence |  |  |  | | 0.46 | |
|  |  |  |  | | (0.99) | |
| Religion (Catholic) |  |  |  | | -0.95 | |
|  |  |  |  | | (0.94) | |
| Religion (Evangelical/ protestant) |  |  |  | | -1.02 | |
|  |  |  |  | | (1.03) | |
| Religion (Jewish) |  |  |  | | -0.24 | |
|  |  |  |  | | (1.22) | |
| Religion (Islamic) |  |  |  | | 0.30 | |
|  |  |  |  | | (0.87) | |
| Religion (No affiliation) |  |  |  | | -1.20 | |
|  |  |  |  | | (0.91) | |
| Religion (Other) |  |  |  | | -1.84 | |
|  |  |  |  | | (1.15) | |
| Money |  |  |  | | 1.44 | |
|  |  |  |  | | (1.34) | |
| Log Likelihood | -10328.85 | -3766.02 | -14170.32 | | -14610.55 | |
| AIC | 20683.70 | 7552.03 | 28370.64 | | 29273.11 | |
| BIC | 20778.60 | 7617.77 | 28486.06 | | 29473.18 | |
| Num. obs. | 10942 | 5293 | 16235 | | 16235 | |
| Groups (subj_nb) | 74 | 71 | 145 | | 145 | |
| ***P<0.001, **P<0.01, *P<0.05. Standard errors in parentheses. AIC, Akaike information criterion; BIC, Bayesian information criterion. | | | | | |  |

1. Trials in which A picked the selfish action (took the bonus)

$$Judgment\left( Selfish action \right)\sim Context + Bonus + Points A + Points B + Version+Task order+Experiment+ \left( 1+Context+Bonus \right| Subject)$$

|  | **Experiment 1** | **Experiment 3** | **Experiments 1 and 3** | **Experiments 1 and 3, demographics** | |
| --- | --- | --- | --- | --- | --- |
| **Context** | **0.57 ***** |  | **0.57 ***** | **0.57 ***** | |
|  | (0.12) |  | (0.12) | (0.12) | |
| **Bonus** | **2.77 ***** | **3.94 ***** | **3.10 ***** | **3.10 ***** | |
|  | (0.12) | (0.17) | (0.10) | (0.10) | |
| **Points A** | **-1.08 ***** | **-0.24 *** | **-0.80 ***** | **-0.80 ***** | |
|  | (0.07) | (0.09) | (0.05) | (0.05) | |
| **Points B** | **-2.02 ***** | **-4.43 ***** | **-2.75 ***** | **-2.75 ***** | |
|  | (0.07) | (0.11) | (0.06) | (0.06) | |
| **Version** | **0.85 *** |  | **0.88 *** | **1.45 **** | |
|  | (0.43) |  | (0.40) | (0.52) | |
| **Task order** |  | **-1.18 **** | **-1.08 *** | -0.73 | |
|  |  | (0.41) | (0.42) | (0.40) | |
| Experiment |  |  | -0.29 | -0.94 | |
|  |  |  | (0.40) | (1.72) | |
| Age |  |  |  | 0.05 | |
|  |  |  |  | (0.06) | |
| Gender (Female) |  |  |  | -0.38 | |
|  |  |  |  | (1.64) | |
| Gender (Male) |  |  |  | -0.57 | |
|  |  |  |  | (1.69) | |
| Politics |  |  |  | 1.08 | |
|  |  |  |  | (0.89) | |
| Community size |  |  |  | 0.51 | |
|  |  |  |  | (0.54) | |
| **Affluence** |  |  |  | **-1.90 **** | |
|  |  |  |  | (0.72) | |
| Religion (Catholic) |  |  |  | 0.71 | |
|  |  |  |  | (0.67) | |
| Religion (Evangelical/ protestant) |  |  |  | 0.21 | |
|  |  |  |  | (0.75) | |
| Religion (Jewish) |  |  |  | -1.35 | |
|  |  |  |  | (0.90) | |
| Religion (Islamic) |  |  |  | -0.15 | |
|  |  |  |  | (0.60) | |
| Religion (No affiliation) |  |  |  | 0.35 | |
|  |  |  |  | (0.65) | |
| Religion (Other) |  |  |  | 1.21 | |
|  |  |  |  | (0.82) | |
| **Money** |  |  |  | -**2.45 *** | |
|  |  |  |  | (0.99) | |
| Log Likelihood | -12654.82 | -6080.86 | -18966.32 | -18951.56 | |
| AIC | 25335.64 | 12181.71 | 37962.63 | 37959.11 | |
| BIC | 25430.54 | 12247.45 | 38078.05 | 38174.56 | |
| Num. obs. | 10939 | 5290 | 16229 | 16229 | |
| Groups (subj_nb) | 74 | 71 | 145 | 145 | |
| ***P<0.001, **P<0.01, *P<0.05. Standard errors in parentheses. AIC, Akaike information criterion; BIC, Bayesian information criterion. | | | | |  |
