# Supplementary material for: A computational account of multiple motives guiding context-dependent prosocial behavior
Source: PLoS Comput Biol. 2025 Apr 21;21(4):e1013032. doi: 10.1371/journal.pcbi.1013032 (PMC12112419; doi:10.1371/journal.pcbi.1013032)
Supplement: S2 Table — Fixed effects coefficient estimates, standard errors, and p-values of the action regressions mixed-effects models, including participants as random effects. The action data were analyzed using a binomial probit model. All continuous independent variables were normalized. Actions from Experiments 2 and 3 were analyzed, showing no significant difference between experiments (P = 0.48), or a marginally significant effect (P = 0.027) when including all demographics. The last model (Experiment 2 and 3 demographics) includes self-reported demographic variables (See S9 Table) and shows a small effect of family affluence on prosocial actions. Adding the demographic variables only marginally increased the model fit (ANOVA, P = 0.048) and did not alter the main coefficients. These statistics show that decisions to act selfishly strongly depend on the specific situations, in particular the cost of prosociality (bonus), consequences of selfish choices (points B), and the specific context of the decisions (Figs 2 and S3). (DOCX) [file pcbi.1013032.s021.docx]

**S2 Table**. **Statistical analysis – Actions Experiment 2 and 3 main effects.** Fixed effects coefficient estimates, standard errors, and p-values of the action regressions mixed-effects models, including participants as random effects. The action data were analyzed using a binomial probit model. All continuous independent variables were normalized. Actions from Experiments 2 and 3 were analyzed, showing no significant difference between experiments (p = 0.48), or a marginally significant effect (p = 0.027) when including all demographics. The last model (Experiment 2 and 3 demographics) includes self-reported demographic variables (See S9 Table) and shows a small effect of family affluence on selfish actions. Adding the demographic variables only marginally increased the model fit (ANOVA, p = 0.048) and did not alter the main coefficients. These statistics show that decisions to act selfishly strongly depend on the specific situations, in particular the cost of prosociality (bonus), consequences of selfish choices (points B), and the specific context of the decisions (Fig 2 and S3 Fig).

$$Selfish action \sim Context + Bonus + Points A + Points B + Version+Task order+ \left( 1+Context + Points A + Points B+Bonus \right| Subject)$$

|  | **Experiment 2** | **Experiment 3** | **Experiments 2 and 3** | **Experiments 2 and 3, demographics** |
| --- | --- | --- | --- | --- |
| (Intercept) | 0.67 | -0.03 | 0.59 * | -1.90 |
|  | (0.36) | (0.24) | (0.29) | (1.32) |
| **Bonus** | **6.65 ***** | **7.87 ***** | **7.24 ***** | **7.25 ***** |
|  | (0.56) | (0.65) | (0.43) | (0.44) |
| **Context** | **0.19 ***** |  | **0.19 ***** | **0.20 ***** |
|  | (0.05) |  | (0.05) | (0.05) |
| Points A | -0.43 | -0.20 | -0.33 * | -0.33 * |
|  | (0.25) | (0.21) | (0.16) | (0.16) |
| **Points B** | **-2.95 ***** | **-4.45 ***** | **-3.65 ***** | **-3.66 ***** |
|  | (0.46) | (0.65) | (0.40) | (0.40) |
| Version | -0.25 |  | -0.23 | -0.12 |
|  | (0.41) |  | (0.37) | (0.36) |
| Task order |  | -0.45 | -0.62 | -0.66 |
|  |  | (0.33) | (0.37) | (0.35) |
| Experiment |  |  | -0.25 | -1.10 * |
|  |  |  | (0.36) | (0.50) |
| Age |  |  |  | 2.10 |
|  |  |  |  | (1.77) |
| Gender (male) |  |  |  | 0.34 |
|  |  |  |  | (0.25) |
| Politics (right) |  |  |  | -0.96 |
|  |  |  |  | (0.72) |
| Community size |  |  |  | 0.90 |
|  |  |  |  | (0.48) |
| Affluence |  |  |  | **1.44 *** |
|  |  |  |  | (0.66) |
| Religion (Evangelical/ protestant) |  |  |  | 0.89 |
|  |  |  |  | (0.55) |
| Religion (Jewish) |  |  |  | 0.21 |
|  |  |  |  | (0.68) |
| Religion (Islamic) |  |  |  | 0.30 |
|  |  |  |  | (0.79) |
| Religion  (No affiliation) |  |  |  | -0.56 |
|  |  |  |  | (0.41) |
| Religion (Other) |  |  |  | 0.48 |
|  |  |  |  | (0.52) |
| Money |  |  |  | 0.73 |
|  |  |  |  | (0.75) |
| AIC | 8514.62 | 5677.92 | 14214.68 | 14105.72 |
| BIC | 8676.97 | 5786.86 | 14403.67 | 14393.20 |
| Log Likelihood | -4236.31 | -2823.96 | -7084.34 | -7017.86 |
| Num. obs. | 16834 | 10537 | 27371 | 27281 |
| Num. groups: subj_nb | 70 | 72 | 142 | 141 |
| ***P<0.001, **P<0.01, *P<0.05. Standard errors in parentheses. AIC, Akaike information criterion; BIC, Bayesian information criterion. | | | | |
