# Supplementary material for: A computational account of multiple motives guiding context-dependent prosocial behavior
Source: PLoS Comput Biol. 2025 Apr 21;21(4):e1013032. doi: 10.1371/journal.pcbi.1013032 (PMC12112419; doi:10.1371/journal.pcbi.1013032)
Supplement: S3 Table — Fixed effects coefficient estimates, standard errors, and p-values of the judgments regressions mixed-effects models using participants as random effects. The judgment data were analyzed using a Cumulative Link Mixed Model. Trials in which Player A picked the selfish action (took the bonus) were used for the analysis. Due to model convergence issues, only the effect of the context (Helping vs Not destroying) was included as a random slope for each participant. Models including efficiency, or score of the worst-off player, as random slopes failed to converge. Judgments from Experiments 1 and 3 were used, showing no significant difference between experiments (P = 0.48). These statistics show that concerns for efficiency and worst-off player affect judgments of selfish actions (Figs 3 and S3). (DOCX) [file pcbi.1013032.s022.docx]

**S3 Table**. **Statistical analysis – Judgments Experiment 1 and 3 efficiency and worst-off player.** Fixed effects coefficient estimates, standard errors, and *p*-values of the judgments regressions mixed-effects models using participants as random effects. The judgment data were analyzed using a Cumulative Link Mixed Model. Trials in which A picked the selfish action (took the bonus) were used for the analysis. Due to model convergence issues, only the effect of the context (helping vs not destroying) was included as a random slope for each participant. Models including efficiency, or score of the worst-off player, as random slopes failed to converge. Judgments from Experiments 1 and 3 were used, showing no significant difference between experiments (*P* = 0.48). These statistics show that concerns for efficiency and worst-off player affect judgments of selfish actions (Fig 3, S3 Fig).

$$Judgment\left( Selfish action \right)\sim Context +Efficiency + Worstoff + Version + \left( 1+Context \right| Subject)$$

|  | **Experiment 1** | **Experiment 3** | **Experiments 1 and 3** |
| --- | --- | --- | --- |
| **Context** | **0.56 ***** |  | **0.57 ***** |
|  | (0.12) |  | (0.12) |
| **Efficiency** | **1.77 ***** | **4.15 ***** | **2.49 ***** |
|  | (0.07) | (0.11) | (0.06) |
| **Worst-off** | **-1.13 ***** | **-0.42 **** | **-0.90 ***** |
|  | (0.10) | (0.14) | (0.08) |
| **Version** | **0.85 *** |  | **0.88 *** |
|  | (0.43) |  | (0.40) |
| **Task order** |  | **-1.18 **** | **-1.07 *** |
|  |  | (0.41) | (0.42) |
| Experiment |  |  | -0.28 |
|  |  |  | (0.40) |
| Log Likelihood | -12733.23 | -6082.84 | -19014.85 |
| AIC | 25490.46 | 12183.68 | 38057.70 |
| BIC | 25578.07 | 12242.84 | 38165.42 |
| Num. obs. | 10939 | 5290 | 16229 |
| Groups (subj_nb) | 74 | 71 | 145 |
| ***P<0.001, **P<0.01, *P<0.05. Standard errors in parentheses. AIC, Akaike information criterion; BIC, Bayesian information criterion. | | | |
