# Supplementary material for: A computational account of multiple motives guiding context-dependent prosocial behavior
Source: PLoS Comput Biol. 2025 Apr 21;21(4):e1013032. doi: 10.1371/journal.pcbi.1013032 (PMC12112419; doi:10.1371/journal.pcbi.1013032)
Supplement: S4 Table — Fixed effects coefficient estimates, standard errors, and p-values of the action regressions mixed-effects models using participants as random effects. The action data were analyzed using a binomial probit model. All continuous independent variables were normalized. Actions from Experiments 2 and 3 were used, showing no significant difference between experiments (P = 0.52). These statistics show that concerns for efficiency but not for the worst-off player affect prosocial actions at the group level (Figs 3 and S3). (DOCX) [file pcbi.1013032.s023.docx]

**S4 Table**. **Statistical analysis - Actions Experiments 2 and 3 efficiency and worst-off player.** Fixed effects coefficient estimates, standard errors, and p-values of the action regressions mixed-effects models using participants as random effects. The action data were analyzed using a binomial probit model. All continuous independent variables were normalized. Actions from Experiments 2 and 3 were used, showing no significant difference between experiments (*P* = 0.52). These statistics show that concerns for efficiency but not for the worst-off player affect selfish actions at the group level (Fig 3, S3 Fig).

$$Selfish action \sim Context + Efficiency+Worstoff + Version+Task order+ \left( 1+Context +Efficiency+Worstoff \right|Subject)$$

|  | **Experiment 2** | **Experiment 3** | **Experiments 2 and 3** |
| --- | --- | --- | --- |
| (Intercept) | 1.72 *** | 0.96 *** | 1.60 *** |
|  | (0.30) | (0.25) | (0.28) |
| **Context** | **0.16 ***** |  | **0.16 ***** |
|  | (0.04) |  | (0.04) |
| Worst-off | 0.16 | **0.46 *** | 0.28 |
|  | (0.27) | (0.18) | (0.17) |
| **Efficiency** | **3.37 ***** | **4.54 ***** | **3.92 ***** |
|  | (0.39) | (0.51) | (0.32) |
| Version | -0.43 |  | -0.34 |
|  | (0.41) |  | (0.38) |
| Task order |  | -0.64 | -0.64 |
|  |  | (0.35) | (0.37) |
| Experiment |  |  | -0.24 |
|  |  |  | (0.36) |
| AIC | 9849.57 | 6563.76 | 16416.72 |
| BIC | 9965.54 | 6636.38 | 16556.41 |
| Log Likelihood | -4909.79 | -3271.88 | -8191.36 |
| Num. obs. | 16834 | 10537 | 27371 |
| Num. groups: subj_nb | 70 | 72 | 142 |
| ***P<0.001, **P<0.01, *P<0.05. Standard errors in parentheses. AIC, Akaike information criterion; BIC, Bayesian information criterion. | | | |
