# Supplementary material for: A computational account of multiple motives guiding context-dependent prosocial behavior
Source: PLoS Comput Biol. 2025 Apr 21;21(4):e1013032. doi: 10.1371/journal.pcbi.1013032 (PMC12112419; doi:10.1371/journal.pcbi.1013032)
Supplement: S5 Table — Utility equations and parameter description. The general utility equation for each action is described by \(U(a)\) where a is the term for a generic action, πA and πB represent the generic payoffs of Players A and B, respectively. The utilities of acting prosocially (\(U(P)\)) or selfishly (\(U(S)\)) are computed for each trial, with \(pA\) and \(pB\) representing the time estimation outcomes of players A and B, respectively, and \(b\) is the bonus offered to player A. \(ΔU = U(P)−U(S)\) represents the difference in utility for prosocial versus selfish actions. All parameters are common to the two contexts, except the bonus discount parameters, which take a different value for each context (δ1 for Destroying and δ2 for Helping). For parsimony, δ1 was estimated relative to a δ2 fixed to 1. The KW model uses data from the judgment session; \(N(a)\) is the (average) judgment emitted in the judgment task for a specific action \(a\). Each action \(a\) is the choice taken by player A (prosocial P or selfish S), in a specific situation (payoff distributions\( pA\), \(pB\), \(b\)) of the action task. N(a) is computed by rescaling the judgments (entered on a scale from 1 to 6) to -1 (very socially inappropriate) and 1 (very socially appropriate) for each situation. For the original version of this model (group model), the appropriateness judgments are averaged across all participants of the judgment task of the corresponding experiment. In an individual variant of the model used in Experiment 3, we used the rating provided by each individual (i.e.,estimating one’s action from their own judgment in each situation). (DOCX) [file pcbi.1013032.s024.docx]

**S5 Table**. **Computational models.** Utility equations and parameter description. The general utility equation for each action is described by $U(a)$ where a is the term for a generic action, π_A_ and π_B_ represent the generic payoffs of Players A and B, respectively. The utilities of acting prosocially ($U(P)$) or selfishly ($U(S)$) are computed for each trial, with $p_{A}$ and $p_{B}$ representing the time estimation outcomes of players A and B, respectively, and $b$ is the bonus offered to player A. $\Delta U = U\left( P \right)-U\left( S \right)$ represents the difference in utility for prosocial versus selfish actions. All parameters are common to the two contexts, except the bonus discount parameters, which take a different value for each context (𝛿1 for Destroying and 𝛿2 for Helping). For parsimony, 𝛿1 was estimated relative to a 𝛿2 fixed to 1. The KW model uses data from the judgment session; $N(a)$ is the (average) judgment emitted in the judgment task for a specific action $a$. Each action $a$ is the choice taken by player A (prosocial C or selfish S), in a specific situation (payoff distributions$p_{A}$, $p_{B}$, $b$) of the action task. N(a) is computed by rescaling the judgments (entered on a scale from 1 to 6) to -1 (very socially inappropriate) and 1 (very socially appropriate) for each situation. For the original version of this model (group model), the appropriateness judgments are averaged across all participants of the judgment task of the corresponding experiment. In an individual variant of the model used in Experiment 3, we used the rating provided by each individual (i.e., predicting one’s action from their own judgment on each situation).

| **Model 1: Fehr-Schmidt (FS)** | **Parameters affecting ΔU** |
| --- | --- |
| $U\left( a \right)=\pi_{A}-\alpha\max\left( \pi_{B}-\pi_{A},0 \right)-\beta max\left( \pi_{A}-\pi_{B},0 \right)$  $U\left( P \right)= p_{A}-\alpha\max\left( p_{B}-p_{A},0 \right)-\beta\max\left( p_{A}-p_{B},0 \right)$  $U\left( S \right)= p_{A}+b - \beta{(p}_{A}+b)$  $\boldsymbol{\Delta}\boldsymbol{U}\mathbf{=}\boldsymbol{\beta}\left[ \boldsymbol{p}_{\boldsymbol{A}}\mathbf{-}\max\left( \boldsymbol{p}_{\boldsymbol{A}}\mathbf{-}\boldsymbol{p}_{\boldsymbol{B}}\mathbf{,0} \right) \right]\mathbf{-}\boldsymbol{\alpha}\max\left( \boldsymbol{p}_{\boldsymbol{B}}\mathbf{-}\boldsymbol{p}_{\boldsymbol{A}}\mathbf{,0} \right)\mathbf{-(1-}\boldsymbol{\beta}\mathbf{)}\boldsymbol{b}$ | Inequity aversion:  - Advantageous (𝛼)  - Disadvantageous (𝛽) |
| **Model 2: Fehr-Schmidt, extended with bonus discount factors (FS 𝛿1)** | **Parameters affecting ΔU** |
| $U\left( a \right)=\pi_{A}-\alpha\max\left( \pi_{B}-\pi_{A},0 \right)-\beta max\left( \pi_{A}-\pi_{B},0 \right)$  $U\left( P \right)= p_{A}-\alpha\max\left( p_{B}-p_{A},0 \right)-\beta\max\left( p_{A}-p_{B},0 \right)$  $U\left( S \right)= p_{A}+\delta_{i}b - \beta(p_{A}+\delta_{i}b )$  $\boldsymbol{\Delta}\boldsymbol{U}\mathbf{=}\boldsymbol{\beta}\left[ \boldsymbol{p}_{\boldsymbol{A}}\mathbf{-}\max\left( \boldsymbol{p}_{\boldsymbol{A}}\mathbf{-}\boldsymbol{p}_{\boldsymbol{B}}\mathbf{,0} \right) \right]\mathbf{-}\boldsymbol{\alpha}\max\left( \boldsymbol{p}_{\boldsymbol{B}}\mathbf{-}\boldsymbol{p}_{\boldsymbol{A}}\mathbf{,0} \right)\mathbf{-(1-}\boldsymbol{\beta}\mathbf{)}\boldsymbol{\delta}_{\boldsymbol{i}}\boldsymbol{b}$ | Inequity aversion:  - Advantageous (𝛼)  - Disadvantageous (𝛽)  Bonus discount (𝛿_1_) |
| **Model 3: Fehr-Schmidt, extended with bias term (FS bias)** | **Parameters affecting ΔU** |
| $U\left( a \right)=\pi_{A}-\alpha\max\left( \pi_{B}-\pi_{A},0 \right)-\beta max\left( \pi_{A}-\pi_{B},0 \right)$  $U\left( P \right)= p_{A}-\alpha\max\left( p_{B}-p_{A},0 \right)-\beta\max\left( p_{A}-p_{B},0 \right)$  $U\left( S \right)= p_{A}+b - \beta(p_{A}+b )$  $\boldsymbol{\Delta}\boldsymbol{U}\mathbf{=}\boldsymbol{bias}\mathbf{+}\boldsymbol{\beta}\left[ \boldsymbol{p}_{\boldsymbol{A}}\mathbf{-}\max\left( \boldsymbol{p}_{\boldsymbol{A}}\mathbf{-}\boldsymbol{p}_{\boldsymbol{B}}\mathbf{,0} \right) \right]\mathbf{-}\boldsymbol{\alpha}\max\left( \boldsymbol{p}_{\boldsymbol{B}}\mathbf{-}\boldsymbol{p}_{\boldsymbol{A}}\mathbf{,0} \right)\mathbf{-(1-}\boldsymbol{\beta}\mathbf{)}\boldsymbol{b}$ | Inequity aversion:  - Advantageous (𝛼)  - Disadvantageous (𝛽)  Baseline preferences (bias) |
| **Model 4: Fehr-Schmidt, extended with bias term and discount factor (FS 𝛿1 bias)** | **Parameters affecting ΔU** |
| $U\left( a \right)=\pi_{A}-\alpha\max\left( \pi_{B}-\pi_{A},0 \right)-\beta max\left( \pi_{A}-\pi_{B},0 \right)$  $U\left( P \right)= p_{A}-\alpha\max\left( p_{B}-p_{A},0 \right)-\beta\max\left( p_{A}-p_{B},0 \right)$  $U\left( S \right)= p_{A}+b - \beta(p_{A}+b )$  $\boldsymbol{\Delta}\boldsymbol{U}\mathbf{=}\boldsymbol{bias}\mathbf{+}\boldsymbol{\beta}\left[ \boldsymbol{p}_{\boldsymbol{A}}\mathbf{-}\max\left( \boldsymbol{p}_{\boldsymbol{A}}\mathbf{-}\boldsymbol{p}_{\boldsymbol{B}}\mathbf{,0} \right) \right]\mathbf{-}\boldsymbol{\alpha}\max\left( \boldsymbol{p}_{\boldsymbol{B}}\mathbf{-}\boldsymbol{p}_{\boldsymbol{A}}\mathbf{,0} \right)\mathbf{-(1-}\boldsymbol{\beta}\mathbf{)}\boldsymbol{\delta}_{\boldsymbol{i}}\boldsymbol{b}$ | Inequity aversion:  - Advantageous (𝛼)  - Disadvantageous (𝛽)  Baseline preferences (bias)  Bonus discount (𝛿_1_) |
| **Model 5: Charness and Rabin (CR)** | **Parameters affecting ΔU** |
| $U\left( a \right)=\left( 1-\gamma\right)\pi_{A}+\gamma[\mu\min\left( \pi_{A},\pi_{B} \right)+(1-\mu)(\pi_{A}+\pi_{B})]$  $U\left( P \right)=\left( 1-\gamma\right)p_{A}+\gamma[\mu\min\left( p_{A},p_{B} \right)+(1-\mu)(p_{A}+p_{B})]$  $U\left( S \right)=\left( 1-\gamma\right){(p}_{A}+b)+\gamma(1-\mu)(p_{A}+b)$  $\boldsymbol{\Delta}\boldsymbol{U}\mathbf{=}\boldsymbol{\gamma\mu}\min\left( \boldsymbol{p}_{\boldsymbol{A}}\mathbf{,}\boldsymbol{p}_{\boldsymbol{B}} \right)\mathbf{+}\boldsymbol{\gamma}\left( \mathbf{1-}\boldsymbol{\mu} \right)\left[ \boldsymbol{p}_{\boldsymbol{B}}\mathbf{-}\boldsymbol{b} \right]\mathbf{-}\left( \mathbf{1-}\boldsymbol{\gamma} \right)\boldsymbol{b}$ | Self-interest (1-𝛾) vs prosocial interest  Efficiency (1-𝜇) vs worst-off player |
| **Model 6: Charness and Rabin, extended with bonus discount factors (CR 𝛿1)** | **Parameters affecting ΔU** |
| $U\left( a \right)=\left( 1-\gamma\right)\pi_{A}+\gamma[\mu\min\left( \pi_{A},\pi_{B} \right)+(1-\mu)(\pi_{A}+\pi_{B})]$  $U\left( P \right)=\left( 1-\gamma\right)p_{A}+\gamma[\mu\min\left( p_{A},p_{B} \right)+(1-\mu)(p_{A}+p_{B})]$  $U\left( S \right)=\left( 1-\gamma\right){(p}_{A}+\delta_{i}b)+\gamma(1-\mu)(p_{A}+\delta_{i}b)$  $\boldsymbol{\Delta}\boldsymbol{U}\mathbf{=}\boldsymbol{\gamma\mu}\min\left( \boldsymbol{p}_{\boldsymbol{A}}\mathbf{,}\boldsymbol{p}_{\boldsymbol{B}} \right)\mathbf{+}\boldsymbol{\gamma}\left( \mathbf{1-}\boldsymbol{\mu} \right)\left[ \boldsymbol{p}_{\boldsymbol{B}}\mathbf{-}\boldsymbol{\delta}_{\boldsymbol{i}}\boldsymbol{b} \right]\mathbf{-}\left( \mathbf{1-}\boldsymbol{\gamma} \right)\boldsymbol{\delta}_{\boldsymbol{i}}\boldsymbol{b}$ | Self-interest (1-𝛾) vs prosocial interest  Efficiency (1-𝜇) vs worst-off player  Bonus discount (𝛿_1_) |
| **Model 7: Charness and Rabin, extended with bias (CR bias)** | **Parameters affecting ΔU** |
| $U\left( a \right)=\left( 1-\gamma\right)\pi_{A}+\gamma[\mu\min\left( \pi_{A},\pi_{B} \right)+(1-\mu)(\pi_{A}+\pi_{B})]$  $U\left( P \right)=\left( 1-\gamma\right)p_{A}+\gamma[\mu\min\left( p_{A},p_{B} \right)+(1-\mu)(p_{A}+p_{B})]$  $U\left( S \right)=\left( 1-\gamma\right){(p}_{A}+b)+\gamma(1-\mu)(p_{A}+b)$  $\boldsymbol{\Delta}\boldsymbol{U}\mathbf{=}\boldsymbol{bias}\mathbf{+}\boldsymbol{\gamma\mu}\min\left( \boldsymbol{p}_{\boldsymbol{A}}\mathbf{,}\boldsymbol{p}_{\boldsymbol{B}} \right)\mathbf{+}\boldsymbol{\gamma}\left( \mathbf{1-}\boldsymbol{\mu} \right)\left[ \boldsymbol{p}_{\boldsymbol{B}}\mathbf{-}\boldsymbol{b} \right]\mathbf{-}\left( \mathbf{1-}\boldsymbol{\gamma} \right)\boldsymbol{b}$ | Self-interest (1-𝛾) vs prosocial interest  Efficiency (1-𝜇) vs worst-off player  Bonus discount (𝛿_1_) |
| **Model 8: Charness and Rabin, extended with bias and discount factor (CR 𝛿1 bias)** | **Parameters affecting ΔU** |
| $U\left( a \right)=\left( 1-\gamma\right)\pi_{A}+\gamma[\mu\min\left( \pi_{A},\pi_{B} \right)+(1-\mu)(\pi_{A}+\pi_{B})]$  $U\left( P \right)=\left( 1-\gamma\right)p_{A}+\gamma[\mu\min\left( p_{A},p_{B} \right)+(1-\mu)(p_{A}+p_{B})]$  $U\left( S \right)=\left( 1-\gamma\right){(p}_{A}+\delta_{i}b)+\gamma(1-\mu)(p_{A}+\delta_{i}b)$  $\boldsymbol{\Delta}\boldsymbol{U}\mathbf{=}\boldsymbol{bias}\mathbf{+}\boldsymbol{\gamma\mu}\min\left( \boldsymbol{p}_{\boldsymbol{A}}\mathbf{,}\boldsymbol{p}_{\boldsymbol{B}} \right)\mathbf{+}\boldsymbol{\gamma}\left( \mathbf{1-}\boldsymbol{\mu} \right)\left[ \boldsymbol{p}_{\boldsymbol{B}}\mathbf{-}\boldsymbol{\delta}_{\boldsymbol{i}}\boldsymbol{b} \right]\mathbf{-}\left( \mathbf{1-}\boldsymbol{\gamma} \right)\boldsymbol{\delta}_{\boldsymbol{i}}\boldsymbol{b}$ | Self-interest (1-𝛾) vs prosocial interest  Efficiency (1-𝜇) vs worst-off player  Bonus discount (𝛿_1_)  Baseline preferences (bias) |
| **Model 9: Tradeoff** | **Parameters affecting ΔU** |
| $\boldsymbol{\Delta}\boldsymbol{U}\mathbf{=}{\left( \mathbf{1-}\boldsymbol{\delta}_{\boldsymbol{i}} \right)\boldsymbol{p}_{\boldsymbol{B}}\mathbf{-}\boldsymbol{\delta}}_{\boldsymbol{i}}\boldsymbol{b}$ | Tradeoff between bonus and Player B’s loss |
| **Model 10: Tradeoff, with a bias term (tradeoff bias)** | **Parameters affecting ΔU** |
| $\boldsymbol{\Delta}\boldsymbol{U}\mathbf{=}\boldsymbol{bias}\mathbf{+}{\left( \mathbf{1-}\boldsymbol{\delta}_{\boldsymbol{i}} \right)\boldsymbol{p}_{\boldsymbol{B}}\mathbf{-}\boldsymbol{\delta}}_{\boldsymbol{i}}\boldsymbol{b}$ | Tradeoff between bonus and Player B’s loss  Bias |
| **Models 11: Krupka and Weber (KW)** | **Parameters affecting ΔU** |
| $U\left( a \right)=\pi_{A}+\delta N(a)$  $U\left( P \right)=p_{A}+\delta N(P,p_{A},p_{B},b)$  $U\left( S \right)={(p}_{A}+b)+\delta N(S,p_{A},p_{B},b)$  $\boldsymbol{\Delta}\boldsymbol{U}\mathbf{=}\boldsymbol{\delta N}\mathbf{(}\boldsymbol{P}\mathbf{,}\boldsymbol{p}_{\boldsymbol{A}}\mathbf{,}\boldsymbol{p}_{\boldsymbol{B}}\mathbf{,}\boldsymbol{b}\mathbf{)-}\boldsymbol{\delta N}\mathbf{(}\boldsymbol{S}\mathbf{,}\boldsymbol{p}_{\boldsymbol{A}}\mathbf{,}\boldsymbol{p}_{\boldsymbol{B}}\mathbf{,}\boldsymbol{b}\mathbf{)-}\boldsymbol{b}$ | Sensitivity to one own’s payoff (σ)  Propensity to follow norms (𝛿)  Appropriateness judgment of action a (N(a)) |
| **Models 12: Krupka and Weber extended with a bias term (KW bias)** | **Parameters affecting ΔU** |
| $U\left( a \right)=\pi_{A}+\delta N(a)$  $U\left( P \right)=p_{A}+\delta N(P,p_{A},p_{B},b)$  $U\left( S \right)={(p}_{A}+b)+\delta N(S,p_{A},p_{B},b)$  $\boldsymbol{\Delta}\boldsymbol{U}\mathbf{=}\boldsymbol{bias}\mathbf{+}\boldsymbol{\delta N}\mathbf{(}\boldsymbol{P}\mathbf{,}\boldsymbol{p}_{\boldsymbol{A}}\mathbf{,}\boldsymbol{p}_{\boldsymbol{B}}\mathbf{,}\boldsymbol{b}\mathbf{)-}\boldsymbol{\delta N}\mathbf{(}\boldsymbol{S}\mathbf{,}\boldsymbol{p}_{\boldsymbol{A}}\mathbf{,}\boldsymbol{p}_{\boldsymbol{B}}\mathbf{,}\boldsymbol{b}\mathbf{)-}\boldsymbol{b}$ | Sensitivity to one own’s payoff (σ)  Propensity to follow norms (𝛿)  Appropriateness judgment of action a (N(a))  Baseline preferences (bias) |
