# Supplementary material for: A computational account of multiple motives guiding context-dependent prosocial behavior
Source: PLoS Comput Biol. 2025 Apr 21;21(4):e1013032. doi: 10.1371/journal.pcbi.1013032 (PMC12112419; doi:10.1371/journal.pcbi.1013032)
Supplement: S6 Table — Estimated elpd (expected log pointwise predictive density) differences between all models fitted to Experiment 1, reflecting the goodness of fits of the different models using an approximate leave-one-out (LOO) cross-validation. (a) Models fitted to action data (SoftMax choice function) (b) Models fitted to judgment data (ordered-probit choice function). Model comparison shows that both actions and judgments are best explained by our extended version of the Charness and Rabin model, including a bias term and a context-specific bonus discount factor. (DOCX) [file pcbi.1013032.s025.docx]

**S6 Table**. **Model comparison Experiments 1 and 2.** Estimated elpd (expected log pointwise predictive density) differences between all models fitted to Experiment 1, reflecting the goodness of fits of the different models using an approximate leave-one-out (LOO) cross-validation [4]. **a.** Models fitted to action data (softmax choice function) **b.** Models fitted to judgment data (ordered-probit choice function). Model comparison shows that both actions and judgments are best explained by our extended version of the Charness and Rabin model, including a bias term and a context-specific bonus discount factor.

1. **Action models**

| **rank** | **Elpd difference** |  | **model** | **Model Number in  S5 Table** |
| --- | --- | --- | --- | --- |
| 1 | 0.00 |  | CR 𝛿1 bias | Model 8 |
| 2 | -23 |  | CR bias | Model 7 |
| 3 | -235 |  | Tradeoff bias | Model 10 |
| 4 | -251 |  | CR 𝛿1 | Model 6 |
| 5 | -275 |  | CR | Model 5 |
| 6 | -494 |  | Tradeoff | Model 9 |
| 7 | -942 |  | KW bias | Model 12 |
| 8 | -1082 |  | FS 𝛿1 bias | Model 4 |
| 9 | -1091 |  | FS bias | Model 3 |
| 10 | -1369 |  | FS 𝛿1 | Model 2 |
| 11 | -1380 |  | FS | Model 1 |
| 12 | -1533 |  | KW | Model 11 |

1. **Judgment models**

| **rank** | **Elpd difference** | **model** | **Model Number in  S5 Table** |
| --- | --- | --- | --- |
| 1 | 0.00 | CR 𝛿1 bias | Model 8 |
| 2 | -322 | FS 𝛿1 bias | Model 4 |
| 3 | -358 | Tradeoff bias | Model 10 |
| 4 | -467 | CR bias | Model 7 |
| 5 | -760 | FS bias | Model 3 |
| 6 | -4382 | CR 𝛿1 | Model 6 |
| 7 | -4482 | Tradeoff | Model 9 |
| 8 | -4862 | FS 𝛿1 | Model 2 |
| 9 | -4904 | CR | Model 5 |
| 10 | -5052 | FS | Model 1 |
