# Supplementary material for: A computational account of multiple motives guiding context-dependent prosocial behavior
Source: PLoS Comput Biol. 2025 Apr 21;21(4):e1013032. doi: 10.1371/journal.pcbi.1013032 (PMC12112419; doi:10.1371/journal.pcbi.1013032)
Supplement: S7 Table — Estimated elpd (expected log pointwise predictive density) differences between all models fitted to Experiment 3, reflecting the goodness of fits of the different models using an approximate leave-one-out (LOO) cross-validation. (a) Models fitted to action data (SoftMax choice function). (b) Models fitted to judgment data (ordered-probit choice function). The KW group models use the average appropriateness ratings of all participants of the experiment for the N(a) term, whereas the KW individual models use the participant-specific appropriateness ratings. For this experiment, participants faced only the Destroying context, the bonus discount factor capturing differences between contexts was therefore not informative, and models including this parameter were excluded for this analysis. This model comparison shows that, for Experiment 3, both actions and judgments are best explained by a trade-off model. However, the difference between the trade-off model and the version of the Charness and Rabin model including a bias term (which best explains behavior in the previous experiment) is very small. (DOCX) [file pcbi.1013032.s026.docx]

**S7 Table**. **Model comparison Experiment 3.** Estimated elpd (expected log pointwise predictive density) differences between all models fitted to Experiment 3, reflecting the goodness of fits of the different models using an approximate leave-one-out (LOO) cross-validation [4]. (**a**) Models fitted to action data (softmax choice function). (**b**) Models fitted to judgment data (ordered-probit choice function). The KW group models use the average appropriateness ratings of all participants of the experiment for the N(a) term, whereas the KW individual models use the participant-specific appropriateness ratings. For this experiment, participants faced only the Destroying context, the bonus discount factor capturing differences between contexts was therefore not informative, and models including this parameter were excluded for this analysis. This model comparison shows that, for Experiment 3, both actions and judgments are best explained by a tradeoff model. However, the difference between the tradeoff model and the version of the Charness and Rabin model including a bias term (which best explains behavior in the previous experiment) is very small.

1. **Action models**

| **rank** | **Elpd difference** | **model** | **Model Number in  S5 Table** |  |  |  |
| --- | --- | --- | --- | --- | --- | --- |
| 1 | 0 | Tradeoff bias | Model 10 |  |  |  |
| 2 | -12 | CR bias | Model 7 |  |  |  |
| 3 | -120 | CR | Model 5 |  |  |  |
| 4 | -144 | Tradeoff | Model 9 |  |  |  |
| 5 | -222 | KW group bias | Model 12 |  |  |  |
| 6 | -772 | KW group | Model 11 |  |  |  |
| 7 | -895 | KW individual bias | Model 12 |  |  |  |
| 8 | -1050 | FS bias | Model 3 |  |  |  |
| 9 | -1217 | FS | Model 1 |  |  |  |

1. **Judgment models**

| **rank** | **Elpd difference** | **model** | **Model Number in  S5 Table** |  |
| --- | --- | --- | --- | --- |
| 1 | 0.0 | Tradeoff bias | Model 10 |  |
| 2 | -446 | CR bias | Model 7 |  |
| 3 | -837 | FS bias | Model 3 |  |
| 4 | -1791 | Tradeoff | Model 9 |  |
| 5 | -2557 | CR | Model 5 |  |
| 6 | -2804 | FS | Model 1 |  |
|  |  |  |  |  |
