# Supplementary material for: A computational account of multiple motives guiding context-dependent prosocial behavior
Source: PLoS Comput Biol. 2025 Apr 21;21(4):e1013032. doi: 10.1371/journal.pcbi.1013032 (PMC12112419; doi:10.1371/journal.pcbi.1013032)
Supplement: S8 Table — Coefficient estimates, standard errors, and p-values of the mean action (a) or judgment (b) regressions fixed effect model using estimated parameters as independent variables. Average behavior and fitted parameters for each participant of Experiments 1 and 3 (judgments) and 2 and 3 (actions) are used. There are no significant differences between the two experiments for either actions (P = 0.45) or judgments (P = 0.18). These statistics show that the four parameters used in the extended version of the Charness and Rabin model (CR bias) are all relevant to explain participants' actions and judgments. (DOCX) [file pcbi.1013032.s027.docx]

**S8 Table**. **Effects of the different model parameters on actions and judgments of Experiments 1 to 3.** Coefficient estimates, standard errors, and p-values of the mean action **(a)** or judgment **(b)** regressions fixed effect model using estimated parameters as independent variables. Average behavior and fitted parameters for each participant of Experiments 1 and 3 (judgments) and 2 and 3 (actions) are used. There are no significant differences between the two experiments for either actions (*P* = 0.45) or judgments (*P* = 0.18). These statistics show that the four parameters used in the extended version of the Charness and Rabin model (CR bias) are all relevant to explain participants’ actions and judgments.

$$mean\left( Choice \right) \sim\gamma+\mu+bias+rho+Experiment$$

|  | **a. Actions** | **b. Judgments** |
| --- | --- | --- |
| (Intercept) | **0.92 ***** | **6.52 ***** |
|  | (0.02) | (0.21) |
| Outcome-based preferences ($\gamma$) | **-0.72 ***** | **-3.20 ***** |
|  | (0.02) | (0.23) |
| Specific goals ($\mu$) | **-0.13 ***** | **-0.66 ***** |
|  | (0.03) | (0.16) |
| Baseline preferences (bias) | **-0.09 ***** | **-0.50 ***** |
|  | (0.00) | (0.03) |
| Deterministic choices ($\rho$ or $\sigma$) | **0.03 ***** | **0.22 ***** |
|  | (0.01) | (0.06) |
| Experiment | -0.01 | 0.08 |
|  | (0.01) | (0.06) |
| R^2^ | 0.94 | 0.81 |
| Adj. R^2^ | 0.94 | 0.80 |
| Num. obs. | 142 | 147 |
| ***P<0.001, **P<0.01, *P<0.05. Standard errors in parentheses. | | |
