# Supplementary material for: A computational account of multiple motives guiding context-dependent prosocial behavior
Source: PLoS Comput Biol. 2025 Apr 21;21(4):e1013032. doi: 10.1371/journal.pcbi.1013032 (PMC12112419; doi:10.1371/journal.pcbi.1013032)
Supplement: S9 Table — Demographic and personality trait questionnaires were collected: Demographic questionnaire, Altruism components of the NEO-PI-R [103] test, Levenson Self-Report Psychopathy Scale [104], Subthreshold Autism Trait Questionnaire [105], Mach-IV [106] Machiavellianism questionnaire, and 28 items of the Interpersonal Reactivity Index (IRI) [107], measuring four aspects of empathy: perspective taking (PT), fantasy (FS), empathic concern (EC), and personal distress (PD). The table reports the formulations of the questions, and the possible answers participants could choose from. Open fields indicate that participants were asked to type in their answer. (DOCX) [file pcbi.1013032.s028.docx]

**S9 Table**. **List of questions of the final survey on demographic and personality traits:** Demographic and personality trait questionnaires were collected: Demographic questionnaire, Altruism components of the NEO-PI-R [103] test, Levenson Self-Report Psychopathy Scale [104], Subthreshold Autism Trait Questionnaire [105], Mach-IV [106] Machiavellianism questionnaire, and 28 items of the Interpersonal Reactivity Index (IRI) [107], measuring four aspects of empathy: perspective taking (PT), fantasy (FS), empathic concern (EC), and personal distress (PD). The table reports the formulations of the questions, and the possible answers participants could choose from. Open fields indicate that participants were asked to type in their answer.

| **Questionnaire** | **Question** | **Possible answers** |
| --- | --- | --- |
| Demographics | Please specify your gender | Open field |
|  | Please enter your year of birth (yyyy) | Open field |
|  | Are you right-handed (enter R), left-handed (enter L), Ambidextrous (enter A) | R, L, A |
|  | Please enter your nationality | Open field |
|  | What is your first language? | Open field |
|  | What is your marital status? (Married, single, in a long-term partnership...) | Open field |
|  | How much money do you have available for your own use per month (minus costs for housing)? | Open field |
|  | Please select the religious affiliation that best describes you | Roman Catholic, Evangelical or Protestant, Jewish, Islamic, No religious affiliation, Other |
|  | Please indicate the direction of your studies | Law, Economics, Medicine or Veterinary Studies, Other Social Sciences, Mathematics and Natural Sciences, Other |
|  | How large was the community in which you lived for the longest period of time? | <2000 to >100 000 Residents |
|  | How affluent is your family relative to other families in Switzerland (or in your home country)? (Affluent is a synonym of wealthy) | 6 points Likert from Below average affluence to Above average affluence |
|  | Where do you stand politically in the left/right spectrum? | 6 points Likert from Left to Right |
| Altruism | I go out of my way to help others if I can. | Strongly Disagree (0), Disagree (1), Neutral (2), Agree (3), or Strongly Agree (4) |
|  | I think of myself as a charitable person. |  |
|  | Most people I know like me. |  |
|  | I’m not known for my generosity. |  |
|  | I generally try to be thoughtful and considerate. |  |
|  | Some people think of me as cold and calculating. |  |
|  | I try to be courteous to everyone I meet. |  |
|  | Some people think I’m selfish and egotistical. |  |
| Psychopathy | Success is based on survival of the fittest; I am not concerned about the losers. | Strongly disagree(0), Disagree (1), Neither agree nor disagree (2), Agree (3) Strongly agree (4) |
|  | I find myself in the same kinds of trouble‚ time after time. |  |
|  | For me‚ what’s right is whatever I can get away with. |  |
|  | I am often bored. |  |
|  | In today’s world‚ I feel justified in doing anything I can get away with to succeed. |  |
|  | I find that I am able to pursue one goal for a long time. |  |
|  | My main purpose in life is getting as many goodies as I can. |  |
|  | I don’t plan anything very far in advance. |  |
|  | Making a lot of money is my most important goal. |  |
|  | I quickly lose interest in tasks I start. |  |
|  | I let others worry about higher values; my main concern is with the bottom line. |  |
|  | Most of my problems are due to the fact that other people just don’t understand me. |  |
|  | People who are stupid enough to get ripped off usually deserve it. |  |
|  | Before I do anything‚ I carefully consider the possible consequences. |  |
|  | Looking out for myself is my top priority. |  |
|  | I have been in a lot of shouting matches with other people. |  |
|  | I tell other people what they want to hear so that they will do what I want them to do. |  |
|  | When I get frustrated‚ I often "let off steam" by blowing my top. |  |
|  | I would be upset if my success came at someone else’s expense. |  |
|  | Love is overrated. |  |
|  | I often admire a really clever scam. |  |
|  | I make a point of trying not to hurt others in pursuit of my goals. |  |
|  | I enjoy manipulating other people’s feelings. |  |
|  | I feel bad if my words or actions cause someone else to feel emotional pain. |  |
|  | Even if I were trying very hard to sell something‚ I wouldn’t lie about it. |  |
|  | Cheating is not justified because it is unfair to others. |  |
| Autism | I like being around other people | False, not at all true, Slightly true, Mainly true, Very true |
|  | I enjoy social situations where I can meet new people and chat (i.e. parties, dances, sports, games) |  |
|  | I seek out and approach others for social interactions |  |
|  | I like to share my enjoyment with others |  |
|  | Others consider me warm, caring, and/or friendly |  |
|  | I respond appropriately to other people’s emotions (for example, comforting someone who is upset) |  |
|  | I can have a back-and-forth conversation (listen well and change topics appropriately) |  |
|  | I use many gestures when speaking with others such as shrugging, ‘‘talking with my hands,’’ nodding my head, etc. |  |
|  | Others think that I am strange or bizarre |  |
|  | I have some behaviors that others consider strange or odd |  |
|  | I sometimes say things that others tell me are rude or inappropriate |  |
|  | I use odd phrases or tend to repeat certain words or phrases over and over again |  |
|  | I am very interested in things related to numbers (i.e. dates, phone numbers, etc.) |  |
|  | I am good at knowing what others are feeling by watching their facial expressions or listening to the tone of their voice |  |
|  | I can sense that someone is not interested in what I’m saying by reading their facial expressions |  |
|  | I make eye contact when talking with others |  |
|  | I am good at using words to express my thoughts and ideas |  |
|  | I have difficulty getting my ideas across to others in a conversation |  |
|  | I have a good imagination |  |
|  | I am comfortable with spontaneity, such as going to new places and trying new things |  |
|  | I tend to stick to routines in my day-to-day life, preferring to do things the same way |  |
|  | I am considered ‘‘laid back’’ and am able to ‘‘go with the flow’’ |  |
|  | I sometimes take things too literally, such as missing the point of a joke or having trouble understanding sarcasm |  |
|  | I tend to focus on individual parts and details more than the big picture |  |
| Machiavellianism | Never tell anyone the real reason you did something unless it is useful to do so. | Strongly Disagree (0), Disagree (1), Neutral (2), Agree (3), or Strongly Agree (4) |
|  | The best way to handle people is to tell them what they want to hear. |  |
|  | One should take action only when sure it is morally right. |  |
|  | Most people are basically good and kind. |  |
|  | It is safest to assume that all people have a vicious streak and it will come out when they are given a chance. |  |
|  | Honesty is the best policy in all cases. |  |
|  | There is no excuse for lying to someone else. |  |
|  | Generally speaking‚ people won’t work hard unless they’re forced to do so. |  |
|  | All in all‚ it is better to be humble and honest than to be important and dishonest. |  |
|  | When you ask someone to do something for you‚ it is best to give the real reasons for wanting it rather than giving reasons which carry more weight. |  |
|  | Most people who get ahead in the world lead clean‚ moral lives. |  |
|  | Anyone who completely trusts anyone else is asking for trouble. |  |
|  | The biggest difference between most criminals and other people is that the criminals are stupid enough to get caught. |  |
|  | Most people are brave. |  |
|  | It is wise to flatter important people. |  |
|  | It is possible to be good in all respects. |  |
|  | P.T. Barnum was wrong when he said that there’s a sucker born every minute. |  |
|  | It is hard to get ahead without cutting corners here and there. |  |
|  | People suffering from incurable diseases should have the choice of being put painlessly to death. |  |
|  | Most people forget more easily the death of their parents than the loss of their property. |  |
| IRI Fantasy scale | I daydream and fantasize, with some regularity, about things that might happen to me. | 5 points Likert scale from 'Does not describe me well' to 'Describes me very well' |
|  | I really get involved with the feelings of the characters in a novel. |  |
|  | I am usually objective when I watch a movie or play, and I don't often get completely caught up in it. |  |
|  | Becoming extremely involved in a good book or movie is somewhat rare for me. |  |
|  | When I watch a good movie, I can very easily put myself in the place of a leading character. |  |
|  | After seeing a play or movie, I have felt as though I were one of the characters. |  |
|  | When I am reading an interesting story or novel, I imagine how I would feel if the events in the story were happening to me. |  |
| IRI Perspective taking | I sometimes find it difficult to see things from the "other guy's" point of view. |  |
|  | I try to look at everybody's side of a disagreement before I make a decision. |  |
|  | I sometimes try to understand my friends better by imagining how things look from their perspective. |  |
|  | If I'm sure I'm right about something, I don't waste much time listening to other people's arguments. |  |
|  | I believe that there are two sides to every question and try to look at them both. |  |
|  | When I'm upset at someone, I usually try to "put myself in his shoes" for a while. |  |
|  | Before criticizing somebody, I try to imagine how I would feel if I were in their place. |  |
| IRI Empathy concern | I often have tender, concerned feelings for people less fortunate than me. |  |
|  | Sometimes I don't feel very sorry for other people when they are having problems. |  |
|  | When I see someone being taken advantage of, I feel kind of protective towards them. |  |
|  | Other people's misfortunes do not usually disturb me a great deal. |  |
|  | When I see someone being treated unfairly, I sometimes don't feel very much pity for them. |  |
|  | I am often quite touched by things that I see happen. |  |
|  | I would describe myself as a pretty soft-hearted person. |  |
| IRI Personal Distress | In emergency situations, I feel apprehensive and ill-at-ease. |  |
|  | I sometimes feel helpless when I am in the middle of a very emotional situation. |  |
|  | When I see someone get hurt, I tend to remain calm. |  |
|  | Being in a tense emotional situation scares me. |  |
|  | I am usually pretty effective in dealing with emergencies. |  |
|  | I tend to lose control during emergencies. |  |
|  | When I see someone who badly needs help in an emergency, I go to pieces. |  |
