# Supplementary material for: A computational account of multiple motives guiding context-dependent prosocial behavior
Source: PLoS Comput Biol. 2025 Apr 21;21(4):e1013032. doi: 10.1371/journal.pcbi.1013032 (PMC12112419; doi:10.1371/journal.pcbi.1013032)
Supplement: S10 Table — Fixed effects coefficient estimates, standard errors, and p-values of mixed-effects regressions models of actions and judgments using participants as random effects. The action data were analyzed using a binomial probit model and judgment data using a Cumulative Link Mixed Model. Due to convergence issues, only random intercepts were used in the judgments analysis, while random slopes were included in the actions analysis. (a) Logistic regression of actions. (b) Logistic regression of actions including demographic variables. (c) Ordinal regression of judgments. (d) Ordinal regression of judgments including demographic variables. Including control demographic variables (See S9 Table) slightly improved the model fits (ANOVA P = 0.049 for actions and P < 0.001 for judgments), but did not alter the main effects coefficients. These statistics show that the behavior of the participants in the pre-exposure tasks replicates the behavior measured in Experiments 1–3, and that the choices of participants of the four exposure groups were mostly comparable (S9 Fig). There were some small differences between the pre-exposure actions of the different groups (significant effects of environment type and direction). However, they were very small in comparison to the effects of the payoffs. (DOCX) [file pcbi.1013032.s029.docx]

**S10 Table**. **Statistical analysis - Experiment 4: Actions and Judgments pre-exposure.** Fixed effects coefficient estimates, standard errors, and p-values of mixed-effects regressions models of actions and judgments using participants as random effects. The action data were analyzed using a binomial probit model and judgment data using a Cumulative Link Mixed Model. Due to convergence issues, only random intercepts were used in the judgments analysis, while random slopes were included in the actions analysis. **(a)** Logistic regression of actions. **(b)** Logistic regression of actions including demographic variables. **(c)** Ordinal regression of judgments. **(d)** Ordinal regression of judgments including demographic variables. Including control demographic variables (See S9 Table) slightly improved the model fits (ANOVA *P* = 0.028 for actions and *P* < 0.001 for judgments), but did not alter the main effects coefficients. These statistics show that the behavior of the participants in the pre-exposure tasks replicates the behavior measured in Experiments 1–3, and that the choices of participants of the four exposure groups were mostly comparable (S9 Fig). There were some small differences between the pre-exposure actions of the different groups (significant effects of environment type and direction). However, they were very small in comparison to the effects of the payoffs.

$$Choice (action or judgment)\sim Bonus + Points A + Points B + Norm type+Direction+Task order+ \left( 1+ Bonus+ Points A + Points B \right| Subject)$$

|  | **a. Actions** | **b. Actions**  **demographics** | **c. Judgments** | | **d. Judgments**  **demographics** | |
| --- | --- | --- | --- | --- | --- | --- |
| (Intercept) | -0.11 | -0.40 |  | |  | |
|  | (0.14) | (0.65) |  | |  | |
| **Bonus** | **6.82 ***** | **6.81 ***** | **4.59 ***** | | **4.59 ***** | |
|  | (0.27) | (0.27) | (0.00) | | (0.08) | |
| **Points B** | **-3.32 ***** | **-3.32 ***** | **-4.36 ***** | | **-4.36 ***** | |
|  | (0.22) | (0.22) | (0.00) | | (0.05) | |
| Task order | **-0.56 ***** | **-0.57 ***** | **-0.70 ***** | **-0.78 ***** | |  |
|  | (0.15) | (0.15) | (0.14) | (0.17) | |  |
| Environment type | -0.02 | -0.01 | -0.19 | -0.22 | |  |
|  | (0.15) | (0.15) | (0.14) | (0.16) | |  |
| Direction | -0.06 | -0.07 | **-0.06 ***** | -0.08 | |  |
|  | (0.08) | (0.08) | (0.00) | (0.08) | |  |
| age |  | -0.00 |  | -0.01 | |  |
|  |  | (0.02) |  | (0.02) | |  |
| Gender (Male) |  | 0.47 ** |  | -0.70 *** | |  |
|  |  | (0.15) |  | (0.17) | |  |
| Gender (Other) |  | 1.35 |  | -0.95 | |  |
|  |  | (0.87) |  | (0.91) | |  |
| Politics (right) |  | 0.75 |  | 0.85 | |  |
|  |  | (0.49) |  | (0.53) | |  |
| Community size |  | 0.43 |  | 0.48 | |  |
|  |  | (0.28) |  | (0.31) | |  |
| Affluence |  | -0.45 |  | -0.79 | |  |
|  |  | (0.39) |  | (0.42) | |  |
| Religion (Evangelical / protestant) |  | -0.06 |  | 0.28 | |  |
|  |  | (0.28) |  | (0.31) | |  |
| Religion (Jewish) |  | 0.56 |  | -0.28 | |  |
|  |  | (1.15) |  | (1.11) | |  |
| Religion (Islamic) |  | -0.26 |  | 1.11 | |  |
|  |  | (0.49) |  | (0.58) | |  |
| Religion (None) |  | -0.10 |  | 0.07 | |  |
|  |  | (0.20) |  | (0.22) | |  |
| Religion (Other) |  | -0.67 * |  | -0.75 * | |  |
|  |  | (0.33) |  | (0.36) | |  |
| Money |  | -0.91 |  | -1.31 | |  |
|  |  | (1.14) |  | (1.34) | |  |
| AIC | 22469.52 | 22470.60 | 69725.41 | 69715.39 | |  |
| BIC | 22571.22 | 22674.00 | 69815.33 | 69903.40 | |  |
| Log Likelihood | -11222.76 | -11211.30 | -34851.71 | -34834.69 | |  |
| Num. obs. | 35420 | 35420 | 26226 | 26226 | |  |
| Num. groups: subj_nb | 357 | 357 | 352 | 352 | |  |
| ***P<0.001, **P<0.01, *P<0.05. Standard errors in parentheses.  AIC, Akaike information criterion; BIC, Bayesian information criterion. | | | | | |  |
