# Supplementary material for: A computational account of multiple motives guiding context-dependent prosocial behavior
Source: PLoS Comput Biol. 2025 Apr 21;21(4):e1013032. doi: 10.1371/journal.pcbi.1013032 (PMC12112419; doi:10.1371/journal.pcbi.1013032)
Supplement: S11 Table — Fixed effects coefficient estimates, standard errors, and p-values of the mixed-effects regression of actions using participants as random effects. The action data were analyzed using a binomial probit model, including random intercept and slopes for each participant, separately for each direction (a: positive and d: negative) to test for the effects of the normative environment type (prescriptive versus descriptive), and separately for the four environments (b, c, e, f) to estimate the effects of each treatment. The dummy variable Phase represents the pre- vs post-exposure choices and is significant for all environments. (a) Positive environments (A + and J+). (b) Frequent prosocial action (A+). (c) Strict judgment (J+). (d) Negative environments (A- and J-). (e) Frequent selfish action. (f) Lenient judgment. These statistics show that participants significantly adapted their actions following exposure, and that the two types of normative environment have a significantly different effect on prosociality erosion (direction -, stronger effect of descriptive norm) but not enhancing prosocial action (direction + , no effect of the Environment type, Fig 4). (DOCX) [file pcbi.1013032.s030.docx]

**S11 Table**. **Statistical analysis - Experiment 4 Effects of exposures to normative environments on participant’s Actions:** Fixed effects coefficient estimates, standard errors, and p-values of the mixed-effects regression of actions using participants as random effects. The action data were analyzed using a binomial probit model, including random intercept and slopes for each participant, separately for each direction (a: positive and d: negative) to test for the effects of the normative environment type (prescriptive versus descriptive), and separately for the four environments (b, c, e, f) to estimate the effects of each treatment. The dummy variable Phase represents the pre- vs post-exposure choices and is significant for all environments. (**a**) Positive environments (A+ and J+). (**b**) Frequent prosocial action (A+). (**c**) Strict judgment (J+). (**d**) Negative environments (A- and J-). (**e**) Frequent selfish action. (**f**) Lenient judgment. These statistics show that participants significantly adapted their actions following exposure, and that the two types of normative environment have a significantly different effect on prosociality erosion (direction -, stronger effect of descriptive norm) but not enhancing prosocial action (direction +, no effect of the Environment type, Fig 4).

$$Selfish action \sim Bonus + PointB +Phase*Norm type + \left( 1+ Bonus + PointB + Phase \right| Subject)$$

|  | **a. Direction +** | **b. A+** | **c. J+** | **d. Direction -** | **e. A-** | **f. J-** |
| --- | --- | --- | --- | --- | --- | --- |
| (Intercept) | 0.02 | 0.19 | -0.27 | -0.66 *** | -0.44 * | -0.11 |
|  | (0.17) | (0.21) | (0.17) | (0.16) | (0.19) | (0.15) |
| **Bonus** | **6.77 ***** | **5.12 ***** | **5.14 ***** | **6.59 ***** | **5.14 ***** | **5.92 ***** |
|  | (0.38) | (0.10) | (0.10) | (0.34) | (0.10) | (0.11) |
| **Points B** | **-3.91 ***** | **-3.00 ***** | **-3.09 ***** | **-2.98 ***** | **-2.01 ***** | **-2.96 ***** |
|  | (0.30) | (0.24) | (0.33) | (0.29) | (0.31) | (0.33) |
| **Environment type** | -0.40 |  |  | 0.52 * |  |  |
|  | (0.22) |  |  | (0.21) |  |  |
| **Phase** | **-0.43 ***** | **-0.34 ***** | **-0.30 ***** | **0.79 ***** | **0.74 ***** | **0.24 **** |
|  | (0.08) | (0.08) | (0.06) | (0.09) | (0.10) | (0.08) |
| **Phase * Environment type** | 0.11 |  |  | -**0.49 ***** |  |  |
|  | (0.11) |  |  | (0.12) |  |  |
| AIC | 21015.29 | 11309.24 | 11957.33 | 20083.38 | 11159.56 | 10465.67 |
| BIC | 21150.99 | 11387.09 | 12035.24 | 20218.95 | 11237.45 | 10543.37 |
| Log Likelihood | -10491.65 | -5644.62 | -5968.66 | -10025.69 | -5569.78 | -5222.83 |
| Num. obs. | 35635 | 17761 | 17874 | 35348 | 17840 | 17508 |
| Num. groups: subj_nb | 179 | 89 | 90 | 178 | 90 | 88 |
| ***P<0.001, **P<0.01, *P<0.05. Standard errors in parentheses. AIC, Akaike information criterion; BIC, Bayesian information criterion. | | | | | | |
