# Supplementary material for: A computational account of multiple motives guiding context-dependent prosocial behavior
Source: PLoS Comput Biol. 2025 Apr 21;21(4):e1013032. doi: 10.1371/journal.pcbi.1013032 (PMC12112419; doi:10.1371/journal.pcbi.1013032)
Supplement: S12 Table — Fixed effects coefficient estimates, standard errors, and p-values of the mixed-effects regression models of judgments using participants as random effects. The judgment data were analyzed using a Cumulative Link Mixed Model including a random intercept for each participant. Trials in which Player A picked the selfish action (took the bonus) were used in this analysis, separately for each direction (a: positive and d: negative) to test for the effects of the normative environment type (prescriptive versus descriptive), and separately for the four environments (b, c, e, f) to estimate the effects of each treatment. The variable Phase represents the pre- vs post-exposure choices and is significant for all environments. (a) Positive environments (A + and J+). (b) Frequent prosocial action (A+). (c) Strict judgment (J+). (d) Negative environments (A- and J-). (e) Frequent selfish action. (f) Lenient judgment. These statistics show that participants significantly adapted their judgments following exposure, and that the two types of normative environments have a significantly different effect on judgments changes, in both positive and negative environments (S10 Fig). (DOCX) [file pcbi.1013032.s031.docx]

**S12 Table.** **Statistical analysis – Experiment 4 Effects of exposures to normative environments on participant’s Judgments.** Fixed effects coefficient estimates, standard errors, and p-values of the mixed-effects regression models of judgments using participants as random effects. The judgment data were analyzed using a Cumulative Link Mixed Model including a random intercept for each participant. Trials in which A picked the selfish action (took the bonus) were used in this analysis, separately for each direction (a: positive and d: negative) to test for the effects of the normative environment type (prescriptive versus descriptive), and separately for the four environments (b, c, e, f) to estimate the effects of each treatment. The variable Phase represents the pre- vs post-exposure choices and is significant for all environments. (**a**) Positive environments (A+ and J+). (**b**) Frequent prosocial action (A+). (**c**) Strict judgment (J+). (**d**) Negative environments (A- and J-). (**e**) Frequent selfish action. (**f**) Lenient judgment. These statistics show that participants significantly adapted their judgments following exposure, and that the two types of normative environments have a significantly different effect on judgments changes, in both positive and negative environments (S10 Fig).

$$Judgment\left( Selfish action \right)\sim Bonus + PointB +Phase*Norm type + \left( 1 \right| Subject)$$

|  | **a. Direction +** | **b. A+** | **c. J+** | **d. Direction -** | **e. A-** | **f. J-** |
| --- | --- | --- | --- | --- | --- | --- |
| **Bonus** | **4.35 ***** | **4.34 ***** | **4.39 ***** | **4.52 ***** | **4.06 ***** | **5.13 ***** |
|  | (0.00) | (0.11) | (0.11) | (0.07) | (0.10) | (0.11) |
| Points B | **-4.80 ***** | **-4.98 ***** | **-4.62 ***** | **-4.26 ***** | **-3.34 ***** | **-5.40 ***** |
|  | (0.00) | (0.07) | (0.07) | (0.05) | (0.06) | (0.07) |
| **Environment type** | **-0.18 ***** |  |  | **-0.06** |  |  |
|  | (0.00) |  |  | (0.19) |  |  |
| **Phase** | **-1.12 ***** | **-1.10 ***** | **-2.59 ***** | **1.16 ***** | **1.06 ***** | **0.38 ***** |
|  | (0.00) | (0.03) | (0.04) | (0.03) | (0.03) | (0.03) |
| **Phase * Environment type** | **-1.42 ***** |  |  | **-0.82 ***** |  |  |
|  | (0.00) |  |  | (0.05) |  |  |
| Log Likelihood | -36149.12 | -16428.50 | -14636.42 | -36341.11 | -19014.74 | -17016.71 |
| AIC | 72318.24 | 32875.00 | 29290.84 | 72704.23 | 38047.47 | 34051.41 |
| BIC | 72400.03 | 32942.28 | 29358.30 | 72794.31 | 38115.04 | 34118.77 |
| Num. obs. | 26345 | 13038 | 13307 | 26609 | 13454 | 13155 |
| Groups (subj_nb) | 179 | 89 | 90 | 178 | 90 | 88 |
| ***P<0.001, **P<0.01, *P<0.05. Standard errors in parentheses. AIC, Akaike information criterion; BIC, Bayesian information criterion. | | | | | | |
