# Supplementary material for: A computational account of multiple motives guiding context-dependent prosocial behavior
Source: PLoS Comput Biol. 2025 Apr 21;21(4):e1013032. doi: 10.1371/journal.pcbi.1013032 (PMC12112419; doi:10.1371/journal.pcbi.1013032)
Supplement: S13 Table — Analysis of variance (ANOVA) of the effects of the normative environment (Environment type and direction) as well as the types of participants (Cluster) on the average changes in judgments and prosocial actions post- versus pre-exposure for each participant. Degrees of freedom (Df), F-value, and p-value are reported for each factor. (a) Judgments. (b) Actions. These statistics show that participants of all clusters adapted their judgments similarly following exposure, but that participants of different clusters changed their actions to a different extent after exposure to the different normative environments. (DOCX) [file pcbi.1013032.s032.docx]

**S13 Table**. **Effects of the different normative environments and clusters on actions and judgments changes.** Analysis of variance (ANOVA) of the effects of the normative environment (Environment type and direction) as well as the types of participants (Cluster) on the average changes in judgments and selfish actions post- versus pre-exposure for each participant. Degrees of freedom (Df), F-value, and p-value are reported for each factor. (**a**) Judgments. (**b**) Actions. These statistics show that participants of all clusters adapted their judgments similarly following exposure, but that participants of different clusters changed their actions to a different extent after exposure to the different normative environments.

|  | **a. Judgments** | | | **b. Actions** | | |
| --- | --- | --- | --- | --- | --- | --- |
|  | **Df** | **F-value** | **p-value** | **Df** | **F-value** | **p-value** |
| Environment type | 1 | 49.17 | **< 0.001** | 1 | 8.08 | **0.005** |
| Direction | 1 | 264.07 | **< 0.001** | 1 | 108.64 | **<0.001** |
| Cluster | 3 | 0.41 | 0.749 | 3 | 1.49 | 0.216 |
| Environment type * Direction | 1 | 0.47 | 0.495 | 1 | 13.62 | **<0.001** |
| Environment type * Cluster | 3 | 0.03 | 0.993 | 3 | 3.04 | **0.029** |
| Direction * Cluster | 3 | 0.36 | 0.781 | 3 | 4.14 | **0.007** |
