# Supplementary material for: A computational account of multiple motives guiding context-dependent prosocial behavior
Source: PLoS Comput Biol. 2025 Apr 21;21(4):e1013032. doi: 10.1371/journal.pcbi.1013032 (PMC12112419; doi:10.1371/journal.pcbi.1013032)
Supplement: S14 Table — Coefficient estimates, standard errors, and p-values of fixed effects regressions of fitted parameters changes (CR bias model), using as independent variables the type of environment (descriptive versus prescriptive environment types) and whether the environment was positive or negative (direction). The order of the tasks was used as a control variable. Participants’ fitted parameters from experiment 4 were used. (a) Baseline preferences (Bias). (b) Outcome-based preferences (γ). (c) Specific goals (μ). These statistics show that the different normative environments had different effects on the parameter changes following exposure. (DOCX) [file pcbi.1013032.s033.docx]

**S14 Table**. **Effects of the different normative environments on parameter change.** Coefficient estimates, standard errors, and p-values of fixed effects regressions of fitted parameters changes (CR bias model), using as independent variables the type of environment (descriptive versus prescriptive environment types) and whether the environment was positive or negative (direction). The order of the tasks was used as a control variable. Participants’ fitted parameters from experiment 4 were used. (a) Baseline preferences (Bias). (b) Outcome-based preferences (γ). (c) Specific goals (μ). These statistics show that the different normative environments had different effects on the parameter changes following exposure.

$$Fitted parameter change \sim Environment type*Direction+Task order+Baseline parameter$$

|  | **a. Baseline**  **preferences** | **b. Outcome-based**  **preferences** | **c. Specific goals** |
| --- | --- | --- | --- |
| (Intercept) | 0.50 ** | 0.70 *** | -0.31 * |
|  | (0.18) | (0.09) | (0.13) |
| **Direction** | **-0.46 ***** | **-0.52 ***** | 0.14 |
|  | (0.11) | (0.06) | (0.08) |
| **Environment type** | 0.04 | **-0.30 ***** | 0.15 |
|  | (0.11) | (0.06) | (0.08) |
| Task order | 0.00 | 0.01 | 0.00 |
|  | (0.03) | (0.02) | (0.02) |
| **Direction * Environment type** | 0.03 | **0.24 ***** | **-0.13 **** |
|  | (0.07) | (0.04) | (0.05) |
| **Baseline parameter** | **0.04 ***** | **0.24 ***** | **0.50 ***** |
|  | (0.01) | (0.03) | (0.06) |
| R^2^ | 0.30 | 0.28 | 0.04 |
| Adj. R^2^ | 0.29 | 0.27 | 0.03 |
| Num. obs. | 358 | 358 | 358 |
| ***P<0.001, **P<0.01, *P<0.05. Standard errors in parentheses. | | | |
