# Supplementary material for: A computational account of multiple motives guiding context-dependent prosocial behavior
Source: PLoS Comput Biol. 2025 Apr 21;21(4):e1013032. doi: 10.1371/journal.pcbi.1013032 (PMC12112419; doi:10.1371/journal.pcbi.1013032)
Supplement: S15 Table — Analysis of variance (ANOVA) of the effects of the types of participants (cluster), type of environment (Environment type: descriptive versus prescriptive behavior), and direction (positive versus negative changes) on the average changes in parameters of the CR bias model post- versus pre- exposure for each participant. Degrees of freedom (Df), F-value, and p-value are reported for each factor. (a) Baseline preferences (Bias). (b) Outcome-based preferences (γ). (c) Specific goals (μ). These statistics showed that changes in the model parameters outcome-based prosocial action and specific goals were significantly different for distinct types of participants (cluster). (DOCX) [file pcbi.1013032.s034.docx]

**S15 Table**. **Effects of the different clusters on parameters change post- versus pre-exposure.** Analysis of variance (ANOVA) of the effects of the types of participants (cluster), type of environment (Environment type: descriptive versus prescriptive behavior), and direction (positive versus negative changes) on the average changes in parameters of the CR bias model post- versus pre- exposure for each participant. Degrees of freedom (Df), F-value, and p-value are reported for each factor. (a) Baseline preferences (Bias). (b) Outcome-based preferences (γ). (c) Specific goals (μ). These statistics showed that changes in the model parameters outcome-based prosocial action and specific goals were significantly different for distinct types of participants (cluster).

$$Fitted parameter change \sim Environment type*Direction*Cluster$$

|  | **a. Baseline preferences** | | | **b. Outcome-based preferences** | | | | **c. Specific Goals** | | |
| --- | --- | --- | --- | --- | --- | --- | --- | --- | --- | --- |
|  | **Df** | **F-value** | **p-value** | **Df** | **F-value** | **p-value** | **Df** | | **F-value** | **p-value** |
| Environment type | 1 | 5.76 | 0.02 | 1 | 13.98 | **< 0.001** | 1 | | 3.62 | 0.06 |
| Direction | 1 | 146.61 | **< 0.001** | 1 | 77.73 | **< 0.001** | 1 | | 4.79 | **0.03** |
| Cluster | 3 | 1.99 | 0.12 | 3 | 11.63 | **< 0.001** | 3 | | 8.59 | **< 0.001** |
| Environment type * Direction | 1 | 0.21 | 0.65 | 1 | 44.88 | **< 0.001** | 1 | | 9.89 | **< 0.001** |
| Environment type * Cluster | 3 | 0.84 | 0.47 | 3 | 0.63 | 0.60 | 3 | | 0.63 | 0.59 |
| Direction * Cluster | 3 | 0.81 | 0.49 | 3 | 0.33 | 0.81 | 3 | | 1.58 | 0.19 |
