# Supplementary material for: A computational account of multiple motives guiding context-dependent prosocial behavior
Source: PLoS Comput Biol. 2025 Apr 21;21(4):e1013032. doi: 10.1371/journal.pcbi.1013032 (PMC12112419; doi:10.1371/journal.pcbi.1013032)
Supplement: S16 Table — Details of the participants (Player A) gender and age for each study, number of participants playing the role of Player B, details of the tasks and experimental sessions. See S1d and S1e Fig and S1 Text for the differences between versions. (DOCX) [file pcbi.1013032.s035.docx]

**S16 Table. Overview of the four experiments**. Details of the participants (Player A) gender and age for each study, number of participants playing the role of Player B, details of the tasks and experimental sessions. See S1d-e Fig and S1 Text for the differences between versions.

| **Experiment** | **Number of participants** | **Gender** | **Mean age** | **Number of B** | **Tasks played** | **Number of trials** | **Session** |
| --- | --- | --- | --- | --- | --- | --- | --- |
| 1 | 75  (Version 1: 29  Version 2: 46) | 30 Females  35 Males | 24.1 | - | Time estimation  Judgment | 25  300 | 1  1 |
| 2 | 70  (Version 1: 33  Version 2: 37) | 35 Females  30 Males | 22.6 | 34 | Time estimation  Action | 181-300  181-300 | 1  1 |
| 3 | 72 | 36 Females  36 Males | 23.8 | 12 | Time estimation  Judgment  Action | 300  150  150 | 1  2  2 |
| 4 | 358 | 206 Females  149 Males  3 Non-binary |  | 50 | Time estimation  Judgment  Action  Exposure  Judgment  Action | 300  75  100  75  75  100 | 1  2  2  2  2  2 |
