# Supplementary material for: A computational account of multiple motives guiding context-dependent prosocial behavior
Source: PLoS Comput Biol. 2025 Apr 21;21(4):e1013032. doi: 10.1371/journal.pcbi.1013032 (PMC12112419; doi:10.1371/journal.pcbi.1013032)
Supplement: Attachment — Submitted filename: plos_com_bio_response_v6_submit.docx [file pcbi.1013032.s037.docx]

Dear editors,

We thank you and the two reviewers for reading our paper and providing valuable comments. We were happy to see that the reviewers were very positive about our paper, and we thank them for the constructive suggestions concerning our scope and analytical approach. In the following sections, we give point-to-point responses to each suggestion and outline the corresponding changes in the revised manuscript (printed in blue font color). We hope you agree that we have successfully addressed all the issues the reviewers raised and that the manuscript is now ready for publication in *PLoS Computational Biology*.

**Comments to the Authors:**

Reviewer #1: I have reviewed an earlier version of this manuscript. The authors have addressed all my comments and I don't have any further suggestion. I think this article can be published as is.

*Response:*

*We thank the reviewer for their previous suggestions and the current positive review!*

Reviewer #2: Review for PCOMPBIOL-D-24-01710
In four behavioral experiments, the authors tested human participants in two tasks: an action task where participants decide whether to sacrifice their own payoff to help others (prosocial action) or not to help (selfish action), and a judgment task where participants judge how socially appropriate the selfish action in a specific scenario is. On the group level, participants’ actions and judgments conformed to similar social norms (Experiments 1 and 2). However, the actions and judgments of the same individual had low (Experiment 4) or even null (Experiment 3) correlations. The authors developed an adapted Charness and Rabin model to fit participants’ action and judgment behaviors, and further identified four clusters of participants based on the fitted parameters, with the clustering pattern robust across different experiments. Different types of participants exhibited different extents of behavioral changes when exposed to information on descriptive social norms (i.e., statistics of other participants’ actions or judgments).

This work has impressed me in three aspects of its findings. First, the same individual may strikingly diverge in their actions and norm judgments. Second, individuals’ actions and judgments may change with their knowledge about descriptive social norms. Third, according to an individual’s computational phenotype, the population falls into four clusters, with different types of individuals subject to the influence of descriptive social norms in different ways. I think these findings are potentially important contributions to the field, uncovering the cognitive computations behind the individual differences in prosocial behaviors. The technical quality of the work is also high. The series of experiments were carefully and cleverly designed. The data analyses and computational modeling were rigorous.

*Response:*

*We thank the reviewer for this very positive evaluation of our work!*

My major comments are mostly about the choice of computational models and the writing, as specified below.

1. Presentation of modeling results.

1.1. Model comparison results. In the Supplement, it says LOO was used as the metric for model comparison. But I did not find any of these results being reported in the paper, either in the main text or in the Supplement. Did I miss anything?

*Response:*

*The LOO ELPD was reported in the supplements in Supplementary Table 6 for experiments 1 and 2, and Supplementary Table 7 for experiment 3. We appreciate that this inclusion late in the supplement may have caused the reviewer to miss these results. We have now added this information also in the manuscript on page 16 so that future readers find the table more easily:*

*“see Supplementary Table 6 and 7 for a report of the expected log pointwise predictive density (elpd) reflecting out-of-sample predictive fit of the models”*

*Vehtari, A., Gelman, A. & Gabry, J. Practical Bayesian model evaluation using leave-one-out cross-validation and WAIC. Stat Comput 27, 1413–1432 (2017). https://doi.org/10.1007/s11222-016-9696-4*

1.2. Model predictions versus data. Supplementary Figure S3 shows model predictions versus data for three models, but only for the main effects of single independent variables. How did the model predictions align with the interaction effects in the data, if any? Could that be visualized?

*Response:*

*We thank the reviewer for this suggestion, which helps the reader to get a better grasp of the interaction effects and their fit by the different models. We have now visualized the interaction between the effects of the bonus and points of player B with colormaps, as a new figure panel in the supplementary material (Supplementary Figure 4). This figure shows the differences between subjects’ behavior and the predictions of the different models, highlighting that the Charness and Rabin model can capture these interactions, while the Krupka and Weber models (1) underestimate the frequency of prosocial actions for low points of B and in cases where the bonus exceeds points B (efficient selfish action), but (2) overestimate prosocial actions for high points of B and in cases where the bonus is smaller than the points B of (efficient prosocial action).*

*(****b****) Experiment 2: Difference between the behavior measured and simulated using the different models. The colors represent the difference in proportion of trials where Player A chose the prosocial actions. Blue colors represent a model underestimation of the prosocial decisions, and red colors a model overestimation of prosocial choices. Black lines represent efficiency thresholds: selfish actions are efficient for trials above the dotted lines, and prosocial actions are efficient for trials below the dashed line. The Krupka and Weber models do not capture the effect of efficiency on the choices of Player A, while the Charness and Rabin model shows no systematic deviation from the measured behavior.*

*We added the reference to this figure in the main manuscript on page 17:
“Simulations of behavior using fitted models illustrate how the CR 𝛿1 model faithfully predicts the average subjects’ behavior regardless of the choice situation (Supplementary Fig 4), while flexibly capturing the behavioral patterns of different subjects (Supplementary Fig 5).”*

2. Alternative models to test. According to Supplementary Table S5, a total of 11 models had been tested. This looks like a comprehensive list, which includes variants of previous models. However, I noticed that some readily conceived model variants were not included. For example, why not enhance the Fehr-Schmidt inequality aversion model with an efficiency term (as several previous studies did)? Besides, the Charness and Rabin model but not the Fehr-Schmidt or the Krupka and Weber model was enhanced by both the bonus discount factor and the bias term, which seems to be unfair. Testing these additional models and comparing the performances of all models may allow the authors not only to find an even better model for human behaviors, but also to identify the specific assumptions that contribute to the success of the winning model.

*Response:*

*We thank the reviewer for their suggestions concerning alternative models! Some of these models are indeed valuable additions and we have included them, for example the*

*Fehr-Schmidt model with an additional bonus discount factor and bias term (Supplementary Tables 5 and 6). However, other suggestions are conceptually and/or methodically problematic; we therefore decided not to implement them and explain why we did not do this.*

*For instance, the bonus discount factor in the Charness-Rabin and Fehr-Schmid models captures systematic choice differences between the two contexts (helping vs not destroying). In the Krupka and Weber model, this difference is already accounted for by the N(.) term, which is the average rating provided in the judgment task in the two situations. Since these ratings differ between contexts, they already capture the context effect, which therefore cannot be modelled with a colinear second term. We therefore only enhanced the Krupka and Weber model by a bias term; we explain this choice in the manuscript (see page 38) so that future readers understand why adding a bonus discount factor to a model that already contains context effects is not appropriate: “Note that the average appropriateness ratings vary across choice situations, capturing differences between contexts that may influence Player A’s decision. We therefore did not enrich the KW model with a bonus discount factor, as this parameter would be colinear and could not capture additional unique influences of contexts.”*

*A similar rationale led us to not include a Fehr-Schmidt model enhanced by an efficiency term. As pointed out in previous research (Engelmann, Dirk, (2012),* [*How not to extend models of inequality aversion*](https://EconPapers.repec.org/RePEc:eee:jeborg:v:81:y:2012:i:2:p:599-605)*, Journal of Economic Behavior & Organization, 81, issue 2, p. 599-605), the parameters of this extended model cannot be identified, and the model in essence reduces to a variant of the Charness and Rabin model (the authors of the article cited above more pointedly describe this model variant as “theoretically flawed and empirically useless”). We therefore decided not to include this model variant in our analyses and again provide the rationale for this choice in the manuscript on page 38: “A model enriching the Fehr-Schmidt model with efficiency has been used in previous research but was later shown to be equivalent to simpler models and not identifiable [110]. We therefore did not include this model variant in our analysis.”*

*Thanks again for the valuable suggestions to check which additional models may be tested and compared - this has strengthened our paper and gives future readers more detailed explanations of the different alternatives.*

3. The writing of the Introduction.
3.1. Lines 58–67: it is a little abrupt to jump from the significance of studying prosocial behaviors to “normative concerns”. What is social norm has not been defined so far. At this point in the paper, the term “normative concerns” also sounds ambiguous to me, which is reminiscent of “normative theories” in decision-making. It is introduced only later that there are two kinds of social norms: prescriptive norm and descriptive norm.

*Response:*

*We thank the reviewer for this feedback about the conceptual ambiguities in our Introduction, and for the suggestions to how these could be improved. We now use more consistent terminology throughout the manuscript and have reordered the Introduction to make sure that the relevant definitions and distinctions are introduced in the beginning. Moreover, we now make it clear that while there are usually several norms that in principle apply to a given situation, different people have different motivations to follow specific norms rather than others. We previously referred to these as “normative concerns” but agree that this may be ambiguous. We have now changed this to “motives” for clarity, which should prevent confusion in future readers.*

3.2. The Introduction may need rewriting to clarify the connection of the current work to previous theories and empirical findings. For example, inequality aversion can be considered as related to a specific norm – the egalitarianism norm.

*Response:*

*Thanks also for this suggestion. We rewrote the introduction so that it first introduces what “norms” are. It then clarifies that while usually several norms may in principle apply to a given situation, behavior is likely guided by the motivation to follow one of these norms more than all others that may also apply. This clarifies that, as suggested by the reviewer, inequality aversion is the motivation to follow the egalitarianism norm rather than other possible norms, and we provide this helpful example in the text on pages 5-6: “Moreover, even when people agree on the (in)appropriateness of actions in a given situation, their actual actions may be affected by multiple motives that can conflict with each other. For example, even though the egalitarian norm for splitting money between two people is widely accepted, other motives such as harm aversion (i.e., preventing that others suffer drastic losses) or efficiency preferences (i.e., preventing that the total benefit across all individuals becomes smaller) have been shown to counteract inequality aversion and can deter people from choosing equal monetary distributions in specific contexts [33–35]. Thus, even though people in a group may hold similar beliefs about what constitutes appropriate behavior (e.g., representation of social norms), the motives determining whether they will take the corresponding prosocial actions in a specific situation may differ strongly across individuals.”*

3.3. In fact, the use of norm or normative concern is a little confusing throughout the paper. For example, all the terms in participants’ utility function are considered some kinds of norms. Meanwhile, what was manipulated in Experiment 4 is the knowledge of the descriptive norm, which is apparently not part of participants’ utility function.

*Response:*

*We agree with the reviewer that the distinction between norms (the expectation of what one ought to do) and the model parameters (the individual motives to follow this norm) is important, and we have now made sure to be very explicit about this.*

*First, we have now changed the term “normative concerns” to “motives” for clarity throughout the whole paper. Second, we have also made it clear that in experiment 4, we manipulate the descriptive norm in order to see how this changes the individual motivation to implement this norm, as captured by the model parameters. For example, on page 8, we state that “Our proposed framework allows us to characterize such individual differences and to compare how individuals of different motivational profiles change their behavior when exposed to environments where people follow norms more or less than before.”. On pages 26-27, we state that “Further analysis of the changes in motives of the different individuals revealed that exposure to different environments did not affect the same decision mechanisms for all contexts and subject types (see Supplementary Results, Supplementary Fig s 16, 17, and Supplementary Tables 14 and 15 for details), highlighting the importance of individual differences in these mechanisms.”*

*We think that these revisions have clarified this important point for the future reader.*

3.4. The proposal of the four normative concerns in the Introduction may also need some rewriting. I could not understand most of them until coming to the modeling part of the Results. For example, please unpack “context-dependent preferences” using concrete examples.

*Response:*

*We now provide such examples for all the different motives considered in the current study on pages 7-8: “More specifically, our framework focuses on four general motives that have been proposed to underlie prosocial actions. First, individuals may have some baseline preferences for prosocial actions [49] that are independent of the specific choice outcomes [51,52] or contexts. At the extremes of such unconditional motivation are people who always behave prosocially or selfishly, regardless of the specific situation [52]. Second, people may show outcome-based prosocial motivation and judge actions differently depending on the stakes (costs and consequences) of the decisions [53–55]. That is, people may weigh the trade-off between their own cost (or benefit) and others’ benefit (or cost) when they make prosocial decisions [56,57]. These stakes can be imbalanced, in that the harm caused by selfish actions may outweigh their benefits. For example, a person stealing a wallet gains the money inside the wallet, while the victim loses the money and must in addition replace personal documents. Third, the specific nature of this outcome-based prosocial motivation may depend on different behavioral goals: Some individuals may be concerned with the efficiency [54,58,59] of different actions, favoring actions that maximize wealth regardless of who benefits from it. Others may instead focus on the harm caused by their actions and try to avoid that others receive bad outcomes, either actively (i.e., helping others out of adversity) or passively (i.e., not causing others adversity when this would benefit oneself) [54,60,61]. Finally, individuals may show context-dependent prosocial motivation and may prefer prosocial actions only in some situations but not in others, even though these actions would have the same consequences. This follows from numerous findings that decisions producing the same outcome may be interpreted and implemented differently, depending on the language used to describe choices [62–64]. For example, the same prisoner’s dilemma games framed as a “stock market” versus a “community” game can elicit less cooperation [65,66]. Framing the same decisions as active versus passive harm also has different effects on behavior and brain activations [64], showing that decisions producing the same outcome may be interpreted and implemented differently depending on the contexts.”*

*We hope that these examples can help readers understand these motives more easily.*

3.5. The use of “harm” also puzzled me. When one decides not to help others getting rewards (in the help versus not help scenario), why should it be considered as “harming” others? Not helping does not alter the status quo. Is such use of “harm” consistent with the literature?

*Response:*

*We agree that the term “harm” may be understood to refer only to the choice to actively do bad things to others, as opposed to accepting that these bad things happen to others even though one could do something about it (helping). However, there is the alternative definition of “harm” as choosing bad rather than good outcomes for others, no matter how this is framed (not helping or destroying). As an example, you clearly harm a friend and can damage your relation with her if you do not speak up and assist her when she is being insulted by others. In order to clarify this, we now speak of harming someone “actively” (taking) or “passively” (not helping), and how the difference between the motivation for avoiding these two forms of harm is captured by the corresponding bonus discount parameter. See page 7: “Others may instead focus on the harm caused by their actions and try to avoid that others receive bad outcomes, either actively (i.e., helping others out of adversity) or passively (i.e., not causing others adversity when this would benefit oneself) [54,60,61]……* *Framing the same decisions as active versus passive harm also has different effects on behavior and brain activations [64], showing that decisions producing the same outcome may be interpreted and implemented differently depending on the contexts.”*

4. Behavioral change after exposure to descriptive norms.

4.1. The authors have shown that the different types of participants classified from model parameters differed in their change in the percentage of choosing prosocial actions. In parallel to this categorical view of the population, is there any correlation between pre-exposure parameters and the percentage change of prosocial choices? Figure 11d only shows an absence of correlation between pre-exposure parameters and the change of the same parameter.

*Response:*

*Thank you for this interesting question. We ran these additional analyses (please see supplementary Figure 13) but found no significant correlations. We have added these results to the main text on page 25: “Behavioral changes associated with the four normative environments differed strongly between subjects (Figure 4d). These individual differences were neither explained by the pre-exposure behavior of the different subjects (Supplementary Figure 13a, correlation between prosocial action rate pre-exposure and prosocial action rate change p (Bonferroni corrected) > 0.4 for all groups) nor by the pre-exposure values of the individual motives (Supplementary Figure 13 c-e, correlation between pre-exposure parameters bias, γ and μ: p (Bonferroni corrected) > 0.07 for all groups and parameters).”*

4.2. Another interesting question is the change of participants across clusters after the exposure. A 4 by 4 matrix would be informative.

*Response:*

*We thank the reviewer for this interesting suggestion, which led us to uncover more interesting behavioral patterns. We have now added the analysis to the supplementary material (Supplementary Figure 18) and have added the following sentence to the results on page 27: “Participants were additionally clustered based on their post-exposure motives. A four-by-four analysis of the pre- vs post- manipulation profiles revealed that most individual kept the same motivational type (73% of participants overall, 67% in the A+, 63% in the A-, 80% in the J+ and 81% J- environments), showing that motivational profiles are generally stable and that most changes in behavior reflect altered strength of individual strategies rather than shifts between strategies. However, some participants adopted different strategies, but most of these changes were heterogeneous and did not systematically switch between specific strategies (see Supplementary Fig 18 for a detailed description of these changes). One exception to this were Harm-sensitive participants, who shifted strategies more than other types: They frequently shifted to Efficiency- (33% of participants in the A+, 15% in the A-, and 38% in the J- environments) or Cost-sensitive (40% of participants in the A+ environment) strategies, showing that they adopted a less prosocial, more situation-dependent strategy when learning about the behavior of others.”*

***Supplementary Figure 18. Change in cluster post exposure: confusion matrix.*** *Participants of experiment 4 were clustered again based on the result of a CR bias model fitted to their post-exposure decisions, The pre- and post- exposure clusters were compared. The colors and numbers represent the percentage of subjects of each pre-exposure cluster being clustered in each of the 4 types post exposure, for the 4 different environments. Most participants remained in the same cluster, showing relative stability of the motivational profiles. However, there were some systematic changes across the minority of people who shifted their motivational profile in response to the change in environment. Unconditional selfish mostly remained unconditional selfish regardless of the environment; only a minority of them shifted towards a cost-sensitive or efficiency sensitive profile when observing strict judgments. A third of cost-sensitive participants shifted their motivations towards efficiency when witnessing frequent prosocial actions, but also lenient judgments, while a third of them became unconditional violators when witnessing frequently selfish actions. The efficiency-sensitive type was mostly stable, but did shift to Unconditional selfish or Harm-sensitive types when witnessing frequent selfish actions, showing that the response of these participants to this environment could strongly vary. Finally Harm-sensitive participants changed types the most often and shifted towards more mitigated strategies (cost- or efficiency- sensitive) when witnessing prosocial actions, or even to Unconditional selfish when witnessing frequent selfish actions.*

5. The across-individual correlation between judgments and actions. No across-individual correlation was found in Experiment 3 between judgments and actions, but a small yet significant correlation (r = -0.36, p < 0.001, Figure S11b) was found for Experiment 4. The lack of correlation in Experiment 3 was highlighted in the main text, while the significant correlation in Experiment 4 was hidden in the Supplement, without a word in the main text. This can be misleading. Had I not read the Supplement, I would have been convinced by Experiment 3 that the same individual’s judgments and actions have no correlation at all. The inconsistency between the two results should be disclosed and discussed in the main text.

*Response:*

*We thank the reviewer for this suggestion – we had indeed forgotten to include a reference to this result in the main text, but we agree that this could be misleading. We now mention and discuss these results in the main text, on page 22:* *“Subjects’ pre-exposure actions and judgments largely replicated the results of Experiments 1 and 3 (Supplementary Figure 10). In this larger sample, the correlation between participants’ behavior in the action and judgment task was again small but now statistically significant (Supplementary Figure 10, Pearson’s correlation r = -0.36, p < 0.001). However, as in the previous experiment, a two-sample Kolmogorov-Smirnov test confirmed a significant difference in the distributions of the actions and judgments (D = 0.86, p < 0.001). Moreover, when comparing the correlations measured in Experiment 3 and 4 using a Fishers’ transformation of the correlation coefficient, we found no significant difference between the two correlations (z-test: z = 1.17, p = 0.084). Thus, actions and judgments were only weakly related, in a similar fashion for both experiments.”*

*As well as in the Discussion section, on page 28: “Focusing on individual behavior, our study shows that even though most subjects agreed on what constitutes a prescriptive norm, prosocial behavior was not universal. In particular, although group judgments were strongly correlated with group actions as described previously [39], subjects’ own judgments were only weakly correlated with their own prosocial choices, and distinct motives influenced judgments versus actions, in line with new research suggesting a distinction between personal and social norms[73].”*

Minor issues:

Abstract: “changing normative environments” is a little ambiguous.

*Response:*

*Thanks, we have changed this to other phrases to avoid potential misleading. For example, in the Abstract, Discussion on page 27, and Materials and Methods on page 34: “contexts with changing social norms”; in the Author summary: “changes in other’s norm compliance”; in the Introduction on page 8: “when exposed to environments where people follow norms more or less than before”, and on page 9 : “changes in others’ behavior* *”.*

Figure 4c. The percentage of participants who did not change their actions is not properly shown in the figure. It looks like a bug in figure rendering.

*Response:*

*Thank you for spotting this bug, we have fixed the display.*

Figure 4d. It might be better to order the four clusters in the same order as Figure 3d.

*Response:*

*We thank the reviewer for noticing this inconsistency in the figure presentation; we have changed the ordering of the clusters accordingly.*

Supplementary Table S5: How was the appropriateness judgment N(.) in the Krupka and Weber model defined?

*Response:*

*We modified the legend of supplementary Table 5 to be more explicit about the definition of N: “*$N(a)$ *is the (average) judgment emitted in the judgment task for a specific action* $a$*. Each action* $a$ *is the choice taken by player A (prosocial C or selfish S), in a specific situation (payoff distributions*$p_{A}$*,* $p_{B}$*,* $b$*) of the action task. N(a) is computed by rescaling the judgments (entered on a scale from 1 to 6) to -1 (very socially inappropriate) and 1 (very socially appropriate) for each situation. For the original version of this model (group model), the appropriateness judgments are averaged across all participants of the judgment task of the corresponding experiment. In an individual variant of the model used in Experiment 3, we used the rating provided by each individual (i.e., predicting one’s action from their own judgment on each situation)”*
